# Targeted KRAS^G12V^ Degradation *in vivo* Elicits Lung Adenocarcinoma Regression with Subsequent Relapse from Dysregulated Proteolysis

**DOI:** 10.1158/0008-5472.CAN-25-5172

**Published:** 2026-08-14

**Authors:** Alberto Martín, Inés M. García-Pérez, Sonia San José, Pep Rojo, Carlos Riego-Mejías, Cristina Teodosio, Bárbara MG. Barbosa, Carolina Sánchez-Zarzalejo, Ignasi Folch-I-Casanovas, Antonia Odena Caballol, Sònia Jarió, Sara Hijazo-Pechero, Silvia M. Rodríguez-López, Rodrigo Entrialgo-Cadierno, Marie-Julie Nokin, José M. Muñoz-Félix, Diana Loa, Elizabeth Guruceaga, Camille Stephan-Otto Attolini, Chiara Ambrogio, Alberto Villanueva, Silvestre Vicent, Antoni Riera, David Santamaría, Cristina Mayor-Ruiz

**Affiliations:** 1 Molecular Mechanisms of Cancer Program, Centro de Investigación del Cáncer (CIC), CSIC- Universidad de Salamanca, Campus Miguel de Unamuno 37007, Salamanca, Spain; 2 Institute for Research in Biomedicine (IRB Barcelona), the Barcelona Institute of Science and Technology (BIST), Bailderi Reixac 10, 08028, Barcelona, Spain; 3 Translational and Clinical Research Program, Cancer Research Center (IBMCC, CSIC- University of Salamanca); Cytometry Service, NUCLEUS; Department of Medicine, University of Salamanca (Universidad de Salamanca), Salamanca, Spain; Institute of Biomedical Research of Salamanca (IBSAL), Salamanca, Spain; Biomedical Research Networking Centre Consortium of Oncology (CIBERONC), Instituto de Salud Carlos III, Madrid, Spain; 4 University of Navarra, Center for Applied Medical Research, Program in Solid Tumors, Pamplona, Spain; 5 Laboratory of Biology of Tumor and Development (LBTD), GIGA-Cancer, University of Liege, Liege, Belgium; 6 Departamento de Bioquímica y Biología Molecular, Universidad de Salamanca, Instituto de Investigación Biomédica de Salamanca (IBSAL), Salamanca, Spain; 7 Servicio de Experimentación Animal, Universidad de Salamanca, Salamanca, Spain; 8 Department of Molecular Biotechnology and Health Sciences, Molecular Biotechnology Center, University of Torino, Torino, Italy; 9 Procure Program, Catalan Institute of Oncology (ICO), L’Hospitalet de Llobregat, Barcelona, Spain; 10 Centro de Investigación Biomédica en Red de Cáncer (CIBERONC), Madrid, Spain; 11 Navarra Health Institute (IDISNA), Pamplona, Spain; 12 University of Navarra, Department of Pathology, Anatomy and Physiology, Pamplona, Spain; 13 Departament de Química Inorgànica i Orgànica, Universitat de Barcelona, Barcelona, Spain

## Abstract

Recent drug discovery breakthroughs led to the approval of KRAS^G12C^ inhibitors in lung adenocarcinoma (LUAD). Unfortunately, clinical responses are often hampered by the rapid resistance onset. Proteolysis-targeting chimeras (PROTACs) have emerged as promising alternatives to traditional inhibition. However, there is limited mechanistic understanding of KRAS degradation *in vivo*. Here, we developed a preclinical LUAD mouse model and demonstrated that targeted oncogenic KRAS degradation induces rapid tumor regression primarily due to cancer cell-autonomous mechanisms. Yet, transcriptional, histological, and immunophenotypic analyses revealed a substantial remodeling of the tumor microenvironment. Notably, disease relapse observed during prolonged PROTAC treatment stemmed mostly from proteolysis machinery dysregulation, indicating resistance mechanisms distinct from those reported upon KRAS inhibition. Collectively, these findings highlight the therapeutic potential of KRAS degradation in LUAD, providing insights into both cell-intrinsic and -extrinsic mechanisms that accompany antitumor responses and support the ongoing clinical exploration of this approach.

## Introduction

RAS GTPases are among the most frequently mutated oncoproteins in cancer with ~30% of all human tumors harboring a mutation in either HRAS, NRAS, or KRAS isoforms (1). The most common activating mutations occur at codons 12, 13, and 61 (1,2). Functionally, these mutations stabilize the active, GTP-bound (ON) state of RAS proteins, thereby increasing oncogenic flux through downstream effectors such as the MAPK pathway (3). KRAS mutations are also found in nearly a quarter of all lung adenocarcinomas (LUAD), the deadliest type of cancer worldwide (1). Although KRAS was one of the first oncogenes identified in human tumors, efforts at developing efficient inhibitors stalled for around four decades (4,5). However, recent drug development breakthroughs led to the approval of the first KRAS^G12C^ inhibitors for LUAD patients, namely sotorasib (AMG510) and adagrasib (MRTX849), greatly renewing the hope of inhibiting oncogenic KRAS through pharmacological tools (6–11). Unfortunately, the clinical efficacy of KRAS^G12C^ inhibitors rapidly declines due to the development of resistance (12–14). Furthermore, these drugs spare non-G12C mutants, which constitute most KRAS alterations in cancer. As such, many drug discovery programs are ongoing to identify inhibitors targeting other KRAS mutants. For instance, a non-covalent KRAS^G12D^ inhibitor (MRTX1133) is already undergoing early clinical testing (15,16). In addition, multi-KRAS inhibitors such as BI-2865, capable of engaging a broad spectrum of KRAS alleles have been reported (17). As observed with sotorasib and adagrasib, preclinical data with additional mutant-KRAS inhibitors suggest extent and rate of response are far from complete (18,19).

Intense efforts are devoted to exploit on- and off-target drug combinations. The combination of KRAS inhibitors with immunotherapy, with inhibitors of upstream and downstream effectors, or with other actionable vulnerabilities are expected to increase the efficacy of anti-KRAS therapies and are currently at different stages of validation including numerous clinical trials (20). Likewise, the development of RAS therapeutics that operate via different mechanisms of action compared to inhibitors could help realize the full potential of direct RAS targeting for cancer treatment. In particular, hijacking cellular circuits with drugs that induce protein-protein proximity offers interesting therapeutic opportunities, as exemplified by several tri-complex inhibitors currently in clinical trials (e.g., RMC-6291, RMC-6236) (21).

Another promising RAS therapeutic concept enabled by proximity-inducing pharmacology is “targeted protein degradation” (TPD). TPD depends on small molecules called ‘*degraders*’ that prompt the destruction of disease-relevant proteins by inducing proximity to effectors of the cellular proteolytic machinery, often an E3 ubiquitin ligase (22–24). While inhibitors typically target a specific enzymatic function, degraders affect protein abundance comparably to a genetic perturbation. The event-driven mechanism of action of degraders compared with occupancy-based traditional inhibitors imparts several unique advantages related to target scope, target fate, potency, and selectivity (25). For instance, degraders do not require high binding affinity for the target protein in order to achieve effective depletion, thus holding the promise of widening the druggable proteome. Two are the prototypical types of degraders: proteolysis-targeting chimeras (PROTACs) and molecular glue degraders. PROTACs feature a dedicated ligand for the target protein and a dedicated E3 binder, both connected by a linker. In contrast, molecular glue degraders are monovalent linker-less compounds and connect target and E3 in a highly cooperative manner, typically by binding only one of them in isolation.

The availability of KRAS inhibitors quickly resulted in the development of initial PROTACs against KRAS^G12C^ and KRAS^G12D^ (26–28). Recently, two multi-KRAS PROTAC tools showed degradation against the most prevalent oncogenic KRAS alleles (29,30). The most clinically advanced PROTACs are ASP3082 (KRAS^G12D^), ASP5834 (panKRAS), PT0253 (KRAS^G12D^), and ARV‐806 (KRAS^G12D^) with phase I studies ongoing (NCT05382559, NCT07094204, NCT06797336, and NCT07023731 respectively). In summary, PROTACs have generated great interest as means against KRAS-driven tumors, suggesting that they may be one of the next long sought-after therapeutic modalities tested in the clinic. Recent elegant studies with LUAD mouse models revealed that while *Kras*^*G12C*^ genetic ablation induced massive tumor regression and prevented resistance *in vivo*, KRAS^G12C^ inhibition caused a limited antitumor response similar to that observed in the clinic, including the rapid onset of resistance (31). The prospect of chemical KRAS degradation mimicking the superior efficacy of genetic ablation compared to inhibition is certainly appealing. Yet, given the different mode of actions of these strategies and the resulting selective pressures, it remains to be determined if disease relapse could be sustained by differing resistance mechanisms.

Although the emphasis of TPD has been typically placed on expanding the druggable proteome, degraders can also help tackle resistance to current anticancer inhibitors (32). The critical opportunity lies in the fact that different pharmacology exhibits distinct resistance mechanisms. Inhibitors typically lead to target-related resistance such as secondary on-target mutations at the binding pocket that decrease inhibitor affinity. The resistance to degraders instead is mainly driven by dysregulated activity of the co-opted E3 ubiquitin ligase (33–35). Leveraging these differences presents a strategic chance to defeat resistance (36–38). From this perspective, and besides other interesting complementary capabilities, the availability of different pharmacological modalities against KRAS is expected to help achieve durable responses. There is, however, limited mechanistic understanding of KRAS degradation *in vivo*.

Here, we set out to evaluate the therapeutic potential of PROTAC-induced degradation of oncogenic KRAS *in vivo*. Given the utmost unmet clinical need, we focused on LUAD. Particularly, on KRAS^G12V^-driven LUAD, a mutant allele with no selective inhibitors or degraders clinically approved. Chemogenetic technologies of targeted protein degradation offer a loss-of-function chemical solution to resolve target biology in cells and in mice even before having the required chemistry in hand (39–43). By leveraging a tag-PROTAC approach, we developed a first-in-class, lentivirus-induced, KRAS^G12V^-degradable mouse model of LUAD. Chemical, tumor-specific modulation of KRAS^G12V^ protein abundance served here to probe the therapeutic potential of oncoprotein degradation, demonstrating profound and durable tumor regression. Recently, Li et al. used a similar tag-based approach and claimed a partial role of the anti-tumor immunity mediated by CD8+ T cells (44). However, our data suggest that tumor regression is mostly due to cancer cell-intrinsic phenotypes. Furthermore, we showed that resistance to KRAS^G12V^-degradation eventually arises as a consequence of dysregulated proteolytic machinery that also goes beyond the E3 hijacked by the PROTAC.

## Materials and Methods

### Cell lines

#### Culture conditions

Murine LUAD cell lines (C1-C6, R1-R13; generated in this study) were grown in DMEM (Gibco, #41966-029, RRID:SCR_019091) supplemented with 10% FBS (Gibco, #A5256701, RRID:SCR_021149) and 1% penicillin/streptomycin (pen/strep) (Gibco, #15140-122, RRID:SCR_021151). MEFs were grown in DMEM (Gibco) supplemented with 15% FBS and 1% pen/strep. MEFs (*Hras*^-/-^;*Nras*^-/-^;*Kras*^*lox/lox*^*;UbCreERT2*^*+/T*^, from the Barbacid Lab, CNIO*)* used in the study were treated with 4-OHT (referred to as RAS^less^) or vehicle (referred to as KRas^lox/lox^) expressing or not exogenous dTAG-KRAS^G12V^. All cell lines were routinely tested for mycoplasma contamination via targeted PCR.

#### Proliferation and viability assays

250 LUAD cells were seeded per well (in tetraplicates) in 96-well plates for apoptotic evaluation. 3 days later, DMSO (Sigma Aldrich, #D5879, RRID:SCR_021111), dTAG-13m or dTAG^V^-1m degraders (0.5µM) were added in combination with PE-Annexin-V (Immunostep, #ANXVPE-200T, RRID:AB_2894541) and cell death was monitored using the AI module of an Incucyte SX5 device (Sartorius, #4816, RRID:SCR_026298) for both cell scanning and analysis.

#### Colony formation assays

MEFs were seeded at 1000 cells/well in 6-well plates (Cultek, #003516). On day 1, the cells were exposed to different treatments (KRAS^lox/lox^ MEFs were treated with 0.6µM 4-hydroxytamoxifen and RAS^less^ + dTAG-KRAS^G12V^ MEFs were treated with 0.3µM dTAG-13). The drugs were refreshed every 2-3 days. After 12-15 days, cells were washed and subsequently fixed and stained with 0.33% (w/v) methylene blue/methanol. After 30min, the cells were washed in distilled water and air-dried. Murine LUAD cell lines were seeded at 500 cells per well in 6-well plates previously coated with laminin (0.01% in PBS). On day 1, the cells were exposed to different treatments (BI-2865 either 8µM or 12µM, dTAG-13 0.5µM, dTAG^V^-1 0.5µM or paclitaxel 0.5µM). The drugs were refreshed every 2-3 days. After 10-15 days, cells were fixed with 4% PFA, washed and stained with 0.33% (w/v) methylene blue/methanol. After 30min, the cells were washed in distilled water and air-dried.

#### Bone marrow-derived macrophage (BMDM) isolation and differentiation

Bone marrow was harvested from the 2 femurs and 2 tibias of 6- to 8- week-old C57BL/6J wild-type animals under sterile conditions. Epiphyseal heads were transected and lumens were flushed with macrophage growing media (DMEM + 1% pen/strep + 10% FBS + 20ng/mL M-CSF (Biolegend, #675402, Macrophage colony-stimulating factor 1, CSF-1_MOUSE, UniProtKB:P07141) using syringes (Braun, #9161406V) coupled with 27G needles (Terumo, #AN*2716R1). Collected bone marrow was immediately filtered through a 70-micron strainer (Falcon, #352350), and the resulting cell suspension was seeded onto 2 non-treated 150-mm Petri dishes (Greiner Bio-One, #639102), with 30mL of macrophage medium per dish. This procedure is required to eliminate adherent mesenchymal stem cells. Fresh macrophage media (30mL) was added after 3 days. After an additional 4 days of culture, macrophages were collected by gently scraping the bottom of the plates and used for co-culture assays.

#### BMDM- tumor cell co-culture, phagocytosis and cell death assays

Co-cultures were composed of ZsGreen labeled LUAD cell lines and BMDMs at a 1:4 ratio, respectively. Both cell types were seeded and dTAG-13m or DMSO (Sigma Aldrich, #D5879, RRID:SCR_021111) were added 24h later. In some cases, 2 day-dTAG-13m-pretreated tumor cell lines were co-cultured with BMDMs. In parallel, tumor cells were separately plated at the same density and subjected to the same treatment conditions as co-cultures. 1 or 2 days after treatment, BMDM-LUAD mixed and pure LUAD cell cultures were collected and analyzed for phagocytosis and cell death by flow cytometry. The immunophenotyping protocol is described below.

Cell death was evaluated using phycoerythrin (PE)-conjugated Annexin V (Immunostep, #ANXVPE-200T, RRID:AB_2894541) and 7-aminoactinomycin D (Immunostep, #7-AAD, RRID:AB_2894528) following the manufacturer’s instructions. Briefly, cells were washed with PBS (540xg for 5min) and then resuspended in 100µL of Annexin V Binding Buffer 1X (Immunostep, BB10X, RRID:SCR_021115). Annexin V-PE (Immunostep, #ANXVPE-200T, RRID:AB_2894541) and 7-AAD (Immunostep, #7-AAD, RRID:AB_2894528) were subsequently added (Supplementary Table S1), and the mixture was incubated for 15min at room temperature (RT) protected from light. Prior to flow cytometric acquisition, samples were further diluted with 200µL of Binding Buffer 1X (Immunostep, BB10X, RRID:SCR_021115). Analysis allowed the identification of viable (annexin V-, 7-AAD-), early apoptotic (annexin V+, 7-ADD-), late apoptotic (annexin V+, 7-AAD+) and necrotic (annexin V-, 7-AAD+) cells.

### Plasmid cloning

The lentiviral vector pLEX305_dTAG-2xHA-KRAS^G12V^ was generated by Gateway cloning (Invitrogen). First, KRAS^G12V^ was cloned into a Gateway compatible donor vector (pDONR223, RRID:Addgene_vector_2395) using the BP clonase after PCR with primers containing BP overhangs. Then, we performed a Gateway LR reaction with pENTR223_KRAS^G12V^ and pLEX305-N-dTAG (Addgene #91797, RRID:Addgene_91797), according to manufacturer’s protocol.

### Chemical compounds

dTAG-13m (previously described in patents WO2017/024318 as FKBP13 and FKBP49-m, and WO2017/024317, WO2016/007612 and WO2016/105518 as FKBP13), dTAG^V^-1m (novel), and IGP002 (novel) were synthesized as described in the Supplementary Information. Tariquidar (ABCB1 inhibitor; HY-10550), MK-517 (ABCC1 inhibitor; HY-19989A), reversan (ABCB1 and ABCC1 inhibitor; HY-107643), and BI-2865 (panKRAS inhibitor; HY-153724) were purchased from MedChemExpress. Carfilzomib (proteasome inhibitor; #S2853) was purchased from LabNet Biotecnica and BI-2493 (panKRAS inhibitor suited for *in vivo* work; #T7206) was synthesized at mg scale by ZellBio.

### Lentiviral production for intranasal delivery of dTAG-KRAS^G12V^

#### Lentivirus Production

293T cells (RRID:CVCL_0063) were expanded to 20x p150 plates. On day 1, cells were transfected using a mix containing 407.5µL DMEM, 2.125µg of pLEX305_dTAG-2xHA-KRAS^G12V^, 4.25µg pMD2.G (Addgene #12259, RRID:Addgene_12259), 8.5µg psPAX2 (Addgene #12260, RRID:Addgene_12260), and 122µL PEI (Polysciences, #24765-1, RRID:SCR_021118) per p150 plate. The transfection mix was vortexed, incubated at RT for 10min, and added to each plate containing 15mL DMEM. After 72h, virus-containing media was collected and concentrated by ultracentrifugation.

#### Ultracentrifugation

Two p150 plates (30mL total) were used per ultracentrifuge tube (Beckman Coulter, #344058, RRID:SCR_011409). Samples were centrifuged at 24,000rpm under vacuum for 1.5h at 4°C (Allegra X-15R Centrifuge, Beckman Coulter, RRID:SCR_018501). The viral pellet was resuspended in 300µL PBS, achieving a 100-fold concentration. Concentrated virus was pooled and aliquoted into 300µL and 50µL volumes for storage and titration, respectively.

#### Virus titration

HAP1 cells (Revvity C631; RRID:CVCL_Y019, 10^5^ per condition) were prepared in 99µL IMDM supplemented with polybrene. A seven-point 1:10 serial dilution of the virus was performed, ranging from 10µL to 0.00001µL. Cells were incubated with virus for 1h at 37°C, with gentle mixing every 15min, then plated on p100 dishes. Puromycin selection was applied 48h post-transduction. After additional 48h, colonies were stained with crystal violet and counted to determine viral titer (1.5 × 10^6^ viral particles/µL).

### Immunoblotting analysis

Cell pellets were washed in cold PBS and lysed with 8M urea 1% CHAPS with constant shaking at 4ºC for 1h. The samples were centrifuged at 11,000×g for 5min at 4°C and the supernatants were collected for protein quantification. For the electrophoresis, 20-25μg of protein with loading buffer (NuPAGE LDS Sample Buffer, 10% β-mercaptoethanol) were denatured for 10min at 70°C, separated on 4-12% SDS-PAGE gels (Invitrogen, RRID:SCR_008417) and transferred into nitrocellulose membranes using the Trans-Blot Turbo Transfer System (RRID:SCR_014973). Membranes were blocked in 5% (w/v) non-fat powder milk/TBST for 1h and later incubated at 4°C overnight with the primary antibodies in TBS-T. Next day, membranes were washed twice in TBS-T and incubated with the secondary antibodies at RT for 1h, washed again and revealed with ChemiDoc MP Imaging system (RRID:SCR_019037).

The primary antibodies used are: β-ACTIN (A5441, used at 1:20000 dilution, RRID:AB_476744), pan-RAS (OP40, used at 1:1000, RRID:AB_10683383), and UBE2Z (HPA007922, used at 1:1000, RRID:AB_1858551) from Sigma-Aldrich. FKBP12 (#sc-136962, RRID:AB_2102734) from Santa Cruz Biotechnology (1:500 dilution). ERK (#9102S, RRID:AB_330744), p-ERK (#9101, RRID:AB_331646), CRBN (#71810, RRID:AB_2799810), Cleaved Caspase-3 (#9661, RRID:AB_2341188) and HSP90 (#4877, RRID:AB_2233307) from Cell Signaling Technology (1:1000 dilution). The secondary antibodies used are the following: anti-rabbit (#111-035-003, RRID:AB_2313567) and anti-mouse (#115-035-003, RRID:AB_10015289) from Jackson ImmunoResearch (1:5000 dilution).

### Chemistry

Detailed synthetic procedures at scale and characterizations for dTAG-13m and dTAG^V^-1m are provided as **Supplementary Data Chemistry**.

### Protease activity measurement

Cells were counted and 3000 cells/well were seeded in a 384-well plate. Cells were treated with either vehicle or 1µM of carfilzomib for 30min. Proteasome activity was measured with Proteasome-Glo™ Chymotrypsin-Like Cell kit (Promega, #G8660, RRID:SCR_006724), according to manufacturer’s specifications.

### *In vivo* studies

All animal experiments were approved by the Bioethics Committee of Salamanca University and the animal experimentation authorities of the autonomous government of Castilla y León (Spain; animal license number #736).

#### Generation of dTAG-KRAS^G12V^ cell lines

7.5×10^7^ pLEX305_dTAG-2xHA-KRAS^G12V^ viral particles in PBS were delivered into C57BL/6J mice (RRID:IMSR_JAX:000664) by intranasal administration. Animals were monitored until signs of distress compatible with LUAD development were observed (average latency of 10 months). Mice were sacrificed and tumors were collected and orthotopically implanted in the lung of NSG recipient mice (RRID:BCBC_4142) as an intermediate step for the development of primary cancer cell lines as previously described (45). dTAG-KRAS^G12V^ LUAD cells used for *in vivo* experiments were infected with lentiviruses expressing ZsGreen (gift from Héctor Peinado, CNIO, Spain) and firefly luciferase (Plateforme des vecteurs viraux du GIGA, ULiege, Belgium). dTAG-13m resistant cell lines (R1-R13) and paired controls (C1-C6) were derived from dTAG-13m refractory tumors and vehicle-treated mice, respectively. Those cell cultures established from resistant tumors were maintained in medium supplemented with dTAG-13m (0.5 µM).

#### Tumor engraftment and KRAS^G12V^ targeted degradation

Mice were kept in ventilated rooms in pathogen-free facilities under controlled temperature (23°C), humidity (50%) and illumination (12h light/12h dark cycle) conditions. After weaning, mice were fed a standard chow diet (Harlan Laboratories, #2918, RRID:SCR_021119). Animals were treated humanely in accordance with standards described in the Guide for the Care and Use of Laboratory Animals considering relevant national and European guidelines. For orthotopic tumor induction, 10^5^ dTAG-KRAS^G12V^ LUAD cells co-expressing both fluorescence and luminescence reporters, were injected in 100μL of PBS via the tail vein of 8-week-old C57BL/6J or NSG mice. Tumor growth was routinely monitored by measuring the bioluminescence signal. For image acquisition, mice were injected intraperitoneally with 100μL of a 30mg/mL D-Luciferin potassium salt solution (Gold Biotechnology, #LUCK-100, RRID:SCR_021120) 10min before imaging and were then anesthetized using 2% isoflurane (Virbac, #CN:575837-4, RRID:SCR_021121). The bioluminescent signal was quantified as total photon flux at lung tumors. Bioluminescence imaging and data processing were performed using the IVIS Lumina S5 imager (Revvity, #CLS148588, RRID:SCR_027425) equipped with the Living Image 4.7.3 software, starting on day 7 after tumor cell injection. In our tail-vein injection model, >95% of bioluminescent signal localized to the chest cavity 1-month post-cell inoculation. Prior to treatment initiation, when tumor size reaches 1×10^8^p/s bioluminescence, mice with similar photon counts were randomized to the control or treatment groups. Then, mice received a daily intraperitoneal 200μL injection of solvent (20% Polyoxyl 15 hydroxystearate USP Glentham Life Sciences, #GX7347 in PBS, control group) or dTAG-13m (40mg/kg, treatment group) during the first week, in which IVIS imaging was conducted at 3 and 7 days post-treatment. Afterwards, a 4-day/week dTAG-13m administration with *in vivo* imaging follow-up once a week was implemented. Undetectable bioluminescence in two consecutive measurements resulted in treatment discontinuation. Subsequently, the 4-day/week degrader administration was restarted upon detection of bioluminescence signal. When measurements revealed recurrent increasing bioluminescence in two consecutive measurements, animals were considered refractory to the treatment and sacrificed. Representative IVIS bioluminescence images are included in Supplementary Data IVIS.

#### Depletion of macrophages with clodronate

Tumor cells were orthotopically engrafted in the lungs of 6-8-week-old C57BL/6J mice via caudal vein as described above. Two weeks post-implantation, daily administration of the targeted degrader dTAG-13m was initiated. To deplete tissue-resident macrophages and circulating monocytes, mice were pre-conditioned with clodronate (Biozol, #LIP-CP-010-010, RRID:SCR_021122) or PBS-containing liposomes (Biozol, #LIP-CP-010-010, RRID:SCR_021123) following a multi-route administration protocol. Specifically, mice received one intravenous (100µL) injection on day -3 and one intraperitoneal (100µL) injection on day -1 relative to the initiation of dTAG-13m treatment (day 0). On day 0, a single intratracheal instillation of the corresponding liposomes was administered to target pulmonary macrophage populations. Maintenance dosing was carried out on day +1 (intravenous, 100µL) and day +4 (intraperitoneal, 100µL) to ensure sustained macrophage depletion throughout the degrader treatment.

Mice were euthanized on day 5 after dTAG-13m administration. Lungs were harvested and processed for flow cytometry analysis according to previously established protocols (see tumor immunophenotyping section), with the exception that magnetic separation of CD45+ and CD45- fractions was omitted.

#### Immunohistochemistry

Tumor-bearing lungs were formalin-fixed for 24h and paraffin embedded according to standard methods. Tissue sections were stained with hematoxylin and eosin for histopathological evaluation. Additional sections were used for immunohistochemical stainings using the following antibodies: HA-tag (Cell Signaling Technology #3724, RRID:AB_1549585), ZsGreen (Takara Bio #632474, RRID:AB_2491179), NK1.1 (Cell Signaling Technology #39197, RRID:AB_2892989), CD4 (Cell Signaling Technology, #25229, RRID:AB_2798898), PD-L1 (Abcam #ab213480, RRID:AB_2773715), αSMA (Agilent, #IR611, RRID:AB_3099704), phospho-Histone H3 pSer10 (Cell Signaling Technology, #9701, RRID:AB_331535), Cleaved Caspase-3 Asp175 (Cell Signaling Technology, #9661, RRID:AB_2341188), pERK1/2 (Cell Signaling Technology, #9101, RRID:AB_331646), Phospho-S6 Ribosomal Protein Ser240/244 (Cell Signaling Technology, #2215, RRID:AB_331682), CD8 (CNIO, #OTO94A) and F4/80 (Bethyl, #A700-209). A quantitative histology score (H-score) considering both the intensity of the staining and the area containing a positive signal was automatically calculated by QuPath v0.5.1 (RRID:SCR_018257).

#### In vivo CD8^+^ T cell depletion

CD8^+^ T cells were depleted by intraperitoneal administration of a monoclonal anti-CD8 antibody. Mice received 200µg of anti-CD8 antibody (AssayGenie, #IVMB0070, RRID:AB_2935826) or an equivalent amount of isotype control antibody (AssayGenie, #IVMB0200) every 3-4 days throughout the experiment. Antibody administration was initiated 48h before starting the dTAG-13m treatment to ensure effective depletion during the course of treatment. The efficiency of CD8^+^ T cell depletion was directly assessed in tumor-bearing lungs by flow cytometry using a T cell-restricted marker panel at 7 days after initiation of dTAG-13m treatment, as well as at the experimental endpoint upon emergence of resistance.

#### In vivo BI-2493 studies

Treatments were initiated when mice reached an average bioluminescence signal of 3×10^8^p/s (dTAG-13m cohort) or 6×10^7^p/s (BI-2493 cohort), BI-2493 (TargetMol, #T72061) was dissolved in an aqueous solution containing 0.5% natrosol (Ashland Industries Europe, #431292) and 5% HPβCD (Sigma Aldrich, #778907) by oral gavage at 60mg/kg twice daily. IVIS imaging was performed weekly until the end of the treatments and tumor-bearing lungs were analyzed by flow cytometry and immunohistochemistry.

### Tumor immunophenotyping studies

#### Sample preparation

Mice were euthanized by cervical dislocation and tumor bearing lungs were dissected and minced by using sterile scissors and added into gentleMACS C tubes (Miltenyi, #130-093-237, RRID:SCR_020270) with 2.5mL of the digestion mix (Miltenyi, #130-096-730, RRID:SCR_020285). Samples were incubated at 37°C during 40min and mechanically processed into single cell suspensions by gentleMACS dissociator (Miltenyi, #130-093-235, RRID:SCR_020267) using program m_impTumor_04.01. After homogenization, samples were filtered through a 70-micron nylon cell strainer (Falcon, #352350), erythrocytes were eliminated using a red-cell lysis buffer (15mM NH4Cl, 1mM KHCO_3_, 0.1mM EDTA). Samples were washed and blocked on ice during 15-20min in FACS buffer (2mM EDTA and 0.5% bovine serum albumin –BSA– in PBS). After blocking, a small volume of the cell suspension was separated for subsequent APC anti-CD45 staining (BioLegend, #147708, RRID:AB_2563540) required for estimating tumor cellularity contribution and immune cell infiltration into the lung tissue by flow cytometry (designated as pre-depleted fraction). The remaining sample was incubated with magnetically labeled mouse anti-CD45 antibody conjugated microbeads (Miltenyi Biotec, #130-052-301, RRID:AB_2877061), in order to separate both CD45+ and CD45- fractions, representing immune and non-immune cell populations respectively. While the CD45+ cell fraction was immunophenotyped (see below), CD45- cells were subjected to a second round of CD45 staining and FACS separation in order to enhance the elimination of immune contaminants. Finally, tumor cells positive for ZsGreen expression were isolated by FACS from this ultrapure CD45- cell fraction and processed for transcriptomic analysis.

#### Immunophenotyping protocols

Characterization of tumor immune cell components was conducted using 2.5×10^5^ cells of the crude tumor suspension and 2×10^6^ cells from the CD45+ purified fraction. All samples were stained employing a stain-lyse-wash procedure. All antibody/reagent references are in Supplementary Table S2. Briefly, samples were washed with PBS at pH 7.4 for 5min at 540xg and pre-incubated with anti-CD3 antibody for C57BL/6J samples, True-Stain Monocyte Blocker TruStain FcX™ PLUS and the Zombie NIR viability marker at a 1:2000 dilution for 30min at RT, protected from light. Subsequently, the remaining antibodies were added, and the samples were incubated in Brilliant Staining Buffer for 30min at RT (protected from light) in a roller. To lyse red blood cells and fix the samples, 1x BD FACS Lysing Solution was added and incubated for 10min at RT (protected from light), followed by centrifugation at 540xg for 5min, washing with PBS containing 0.5% BSA, 0.1% sodium azide, and 2mM EDTA (pH 7.4), and resuspension in 400µL of PBS prior to analysis.

Immunophenotypic analysis of *in cellulo* co-culture samples was performed employing a stain-wash approach. Briefly, cells were incubated with antibodies (see Supplementary Table S1), Brilliant Staining Buffer Plus, True-Stain Monocyte Blocker, TruStain FcX™ PLUS, and the Zombie NIR viability marker at a 1:2000 dilution for 30min at RT, protected from light. Subsequently, the remaining antibodies (see Supplementary Table S1) were added, and the samples incubated in Brilliant Staining Buffer Plus for 30min at RT (protected from light). Following incubation, cells were washed with PBS containing 0.5% BSA, 0.1% sodium azide, and 2mM EDTA (pH 7.4) by centrifugation at 540xg for 5min. Finally, cells were resuspended in 400µL of PBS prior to analysis.

#### Flow cytometry data acquisition and analysis

Data acquisition was performed using an Aurora spectral flow cytometer (Cytek, #Aurora 5L, RRID:SCR_019826) equipped with five lasers (355nm, 405nm, 488nm, 561nm, 640 nm). Daily instrument setup and quality control were conducted according to manufacturer instructions prior to sample measurement. Single-stained reference controls for each fluorochrome in the antibody combination, as well as an unstained control sample, were processed identically to the multicolor-stained samples to ensure accurate spectral unmixing. The resulting unmixing matrix was generated using SpectroFlo software (v3.3.0; Cytek, RRID:SCR_025494).

For analysis of tumor bearing lungs and co-culture samples processed for flow cytometry, dead cells were first excluded based on the expression of the Zombie marker. Subsequently, doublets were removed by gating on forward scatter area (FSC-A) versus forward scatter height (FSC-H). When appropriate, non-lysed red blood cells were excluded from the analysis using the side scatter (SSC) signal on both blue and violet lasers.

For comprehensive *ex vivo* immunophenotyping of tumor bearing lungs and *in cellulo* co-culture experiments, cancer cells were identified as ZsGreen+CD45-, while leukocytes were defined as CD45+ cells. Specific immune cell populations were subsequently identified based on their immunophenotypic profiles, as detailed in Supplementary Table S2. Importantly, all the immune cell populations analyzed were normalized to the tumor cell compartment (ZsGreen+CD45-).

Phagocytosis index was calculated according to the formula: phagocytic cells (CD45+ZsGreen+)/tumor cells (CD45-ZsGreen+)x 100 × Mean ZsGreen MFI (CD45+ZsGreen+)/10^5^ (*in cellulo* assays) or /10^6^ (*in vivo* assays).

All flow cytometric data analysis was performed with Infinicyt software version 2.1.0.a (BD Biosciences, RRID:SCR_026033).

### Transcriptomic studies

#### RNA sequencing

ZsGreen+CD45-LUAD cells were sent to CeGaT GmbH for RNA isolation (MagMax, ThermoFisher, RRID:SCR_023773), library preparation (SmartSeq stranded, Takara Bio, RRID:SCR_021372) and sequencing (NovaSeq6000, Illumina, RRID:SCR_016387). Bioinformatic analysis included demultiplexing using Illumina bcl2fastq (v2.20, RRID:SCR_015058) and FastQC analysis (v0.11.5-cegat, RRID:SCR_014583) prior to completing in-house bioinformatics studies (see below).

#### Bioinformatic pipeline

RNA sequencing data analysis was performed using the following workflow: (1) the quality of the samples was verified using FastQC software (RRID:SCR_014583) (https://www.bioinformatics.babraham.ac.uk/projects/fastqc/); (2) the alignment of reads to the mouse genome (GRCm39) was performed using STAR (RRID:SCR_004463); (3) gene expression quantification using read counts of exonic gene regions was carried out with featureCounts (RRID:SCR_012919); (4) the gene annotation reference was Gencode M34 (RRID:SCR_014966); and (5) differential expression statistical analysis was performed using R/Bioconductor. Data is available at the GEO database (RRID:SCR_00501): GSE301303. Gene expression data was normalized with edgeR (RRID:SCR_012802) and voom. After quality assessment and outlier detection using R/Bioconductor (RRID:SCR_006442), a filtering process was performed. Genes with read counts lower than 6 in more than the 50% of the samples of all the studied conditions were considered as not expressed in the experiment under study. LIMMA (RRID:SCR_010943) (78) was used to identify the genes with significant differential expression between experimental conditions. Further functional and clustering analyses were performed and graphical representations were generated using clusterProfiler (RRID:SCR_016884) and R/Bioconductor. The functional analyses included Gene Set Enrichment Analysis (GSEA) with MsigDB Hallmarks collection of gene sets (RRID:SCR_016863). Additional GSEA analyses were performed using a recently reported KRAS-dependent transcriptome (51).

### Sanger sequencing of dTAG-KRAS in LUAD-derived cell lines

LUAD cells were harvested and centrifuged at 3,000 g to obtain pellets. DNA was extracted using the NucleoSpin Tissue kit (#740952.250, dD Biolab SLU), according to manufacturer’s instructions. One PCR reaction contained 0.25µL Q5 high-fidelity DNA polymerase (#M0491L, New England Biolabs), 5µL 5X buffer, 0.5µL dNTP mix (#N0447S, Thermo Scientific), 1.25µL of 100mM forward primer (CAAGCCTCCGGAGCGCACG), 1.25µL of 100mM reverse primer (AGCAAACACAGTGCACACCAC), and water to reach 25µL. The program to achieve target amplification was: 30s at 98ºC, 7s at 98ºC, 20s at 56ºC, 35s at 72ºC, for 35 cycles; 2min at 72ºC. Amplified DNA was purified according to manufacturer’s instructions by using the NucleoSpin Gel and PCR Clean‐up Mini kit (#22740609.250, Cultek, S.L.U.) and sent to Macrogen for Sanger sequencing (RRID:SCR_014454).

### Proteomics

#### Sample collection

15×10^6^ cells of each cell line were seeded in triplicates the day before collection. To collect, cells were washed with PBS and trypsinized until detached. After collection into a Falcon and centrifuged for 5min at 300xg, pellets were washed three times with cold PBS and then snap-frozen in liquid nitrogen and stored at -80ºC until processed.

#### Cell Lysis and Digestion

Cell pellets were lysed using Beatbox according to the manufacturer’s instructions. In brief, each cell pellet sample was introduced in an individual BeatBox tube. PreOmics’ LYSE buffer and Pierce™ Universal Nuclease for Cell Lysis (Thermo Scientific) were added. Samples were homogenized into the BeatBox instrument where the settings were set at STANDARD for 2 cycles (10min each). Cell debris was removed by centrifugation at 16000g for 5min at 20ºC and supernatant was transferred to new tubes. Protein concentration was determined using the Pierce™ BCA protein Assay (Pierce Biotechnology). 100µg of protein per sample were digested following the PreOmics digestion protocol (iST Sample Preparation Kit 96x, P.O.00027, PreOmics) using Trypsin/LysC mix as enzyme. Incubation was done at 500rpm for 2h at 37ºC. The tryptic peptides eluted were dried down in a speed vacuum centrifuge (Eppendorf).

#### RP Liquid Chromatography Mass Spectrometry

Mass spectrometry was performed on an Orbitrap Eclipse Tribrid mass spectrometer (ThermoFisher Scientific, RRID:SCR_022212) coupled to a EVOSEP One via nanoEasy Spray Source interface with a stainless-steel emitter (EV-1086 EVOSEP, RRID:SCR_025176). Tryptic peptides were loaded onto the EVOTIP following the manufacturer’s instructions. The analytical column was a 15cmX150µm ID, with Dr Maisch C18 AQ 1.9µm beads. The eluents were 0.1% formic acid in water and 0.1% formic acid in acetonitrile. The EVOSEP One method was 15 SPD (88min gradient) and the flow rate 220nL/min. The mass spectrometer was operated in targeted mode, acquiring data with a tailored Parallel Reaction Monitoring (PRM) method. Unique peptides corresponding to 23 proteins of interest were selected for quantification in 21 samples. Full MS1 scans were acquired in the Orbitrap with a scan range of 350-1400 m/z and a resolution of 120,000 (at 200 m/z). Automatic gain control (AGC) was set to a target of 4×10^5^ and a maximum injection time of 50ms. Only MS1 precursors corresponding to the peptides of interest were subjected to MS2 fragmentation and were acquired in the Orbitrap with a resolution of 15,000 (at 200 m/z). AGC was set to a target of 1 × 10^5^ and a maximum injection time of 22ms. Higher energy collision induced dissociation was applied with a normalized collision energy of 28 or 30% depending on the m/z (see Supplementary Table S3). Orbitrap Eclipse Tune Application 3.5.3890 (RRID:SCR_014593) and Xcalibur version 4.6.67.17 (RRID:SCR_014593) were used to operate the instrument and to acquire data, respectively. The mass spectrometry proteomics data has been deposited at PRIDE with the identifier PXD058734.

#### Data Analysis

Acquired raw data files were searched against Mouse SwissProt database (v. June 2024 containing the proteins of interest, RRID:SCR_002380) using the Proteome Discoverer 3.0.1.27 software (RRID:SCR_014477), with Sequest HT as the search engine. The Percolator validation node was used to remove false positives with a false discovery rate (FDR) of 1% at the peptide level. Data were searched with mass tolerances of ±10ppm and ±0.02Da on the precursor and fragment ions, respectively. These results were used to build a spectrum library to facilitate the integration of the peaks for the quantitative analysis. For that, the raw data files were analyzed in Skyline v.24.1.0.199 (RRID:SCR_014080). Peaks were picked automatically, using the default Skyline peak picking model and Savitzky–Golay smoothing was applied. Areas corresponding to the selected peptides were reviewed and after integration and manual validation of the available transition peak areas, total areas for each peptide were exported in .csv format to be further analyzed. Protein quantitation was done by performing MaxLFQ algorithm over the tracked peptide areas for a given protein, using diann_maxlfq from the diann package of the R statistical software (v4.3.0; R Core Team 2023, RRID:SCR_022865). Comparison between groups was done (each resistant cell line vs. control). For each comparison, estimated log2 fold changes were calculated from the mean of each cell line triplicate.

### SNP calling from bulk RNAseq

#### Custom Genome Construction

A custom genome was generated by incorporating the dTAG-2xHA-KRAS^G12V^ sequence into the *Mus musculus* version 10 genome assembly.

#### Gene Annotation

Coding exon regions from genes of interest were extracted from the Ensembl database (RRID:SCR_002344) using the biomaRt package in R (RRID:SCR_019214).

#### Variant Discovery

To identify new variants (SNPs and Indels) for each sample in the regions of interest, the RNA-seq short variant discovery pipeline from the GATK website was utilized with minor customizations. RNA-seq reads were aligned using a two-step alignment strategy with STAR (v2.7.10a, RRID:SCR_004463). First alignment was performed using outFilterMismatchNoverLmax=0.05; outFilterMatchNmin=25; genomeLoad=LoadAndRemove and outSAMstrandField=intronMotif as parameters. A genome generation step (--genomeGenerate) was performed with the parameter sjdbOverhang=50. Second Alignment was performed using genomeLoad=LoadAndRemove and outSAMstrandField=intronMotif as parameters.

Post-alignment data cleanup was performed using Picard Tools (v1.98, RRID:SCR_006525). The following steps and parameters were applied: AddOrReplaceReadGroups with default settings; MarkDuplicates with REMOVE_DUPLICATES=TRUE; ASSUME_SORTED=TRUE and VALIDATION_STRINGENCY=LENIENT as parameters and ReorderSam with ALLOW_CONTIG_LENGTH_DISCORDANCE=TRUE.

Variant calling was conducted using GATK (v4.5.0.0, RRID:SCR_001876): Data was prepared for variant calling using the SplitNCigarReads function and nucleotide recalibration was conducted by applying BaseRecalibrator utilizing the dbSNP137 database (RRID:SCR_002338) for known variants and ApplyBQSR to apply base quality score recalibration. Variants were identified specifically within the exonic regions of the selected genes using the HaplotypeCaller function with parameters ERC=GVCF; dont-use-soft-clipped-bases=TRUE and standard-min-confidence-threshold-for-calling=20.0. Then, a variant filtering step was performed using the VariantFiltration with parameters cluster-window-size=35; cluster-size=3; FS>30.0 and QD<2.0.

#### Joint cohort genotyping, filtering, and annotation

Cohort-level joint genotyping was performed using GATK (v4.5.0.0). Briefly, per-sample GVCF files were imported into a GenomicsDB workspace with GenomicsDBImport, followed by joint genotyping with GenotypeGVCFs to generate a multi-sample variant call set. SNPs and indels were then separated using SelectVariants. Hard filtering was performed with VariantFiltration using variant-type-specific thresholds: SNPs were filtered with QD<2.0 and FS>60.0, while indels were filtered with QD<2.0 and FS>200.0. Filtered variants were then functionally annotated using SnpEff.

## Results

### Novel LUAD KRAS^G12V^-degradable syngeneic mouse models enable PROTAC-dependent phenotyping *in vivo*

**Fig. 1:**
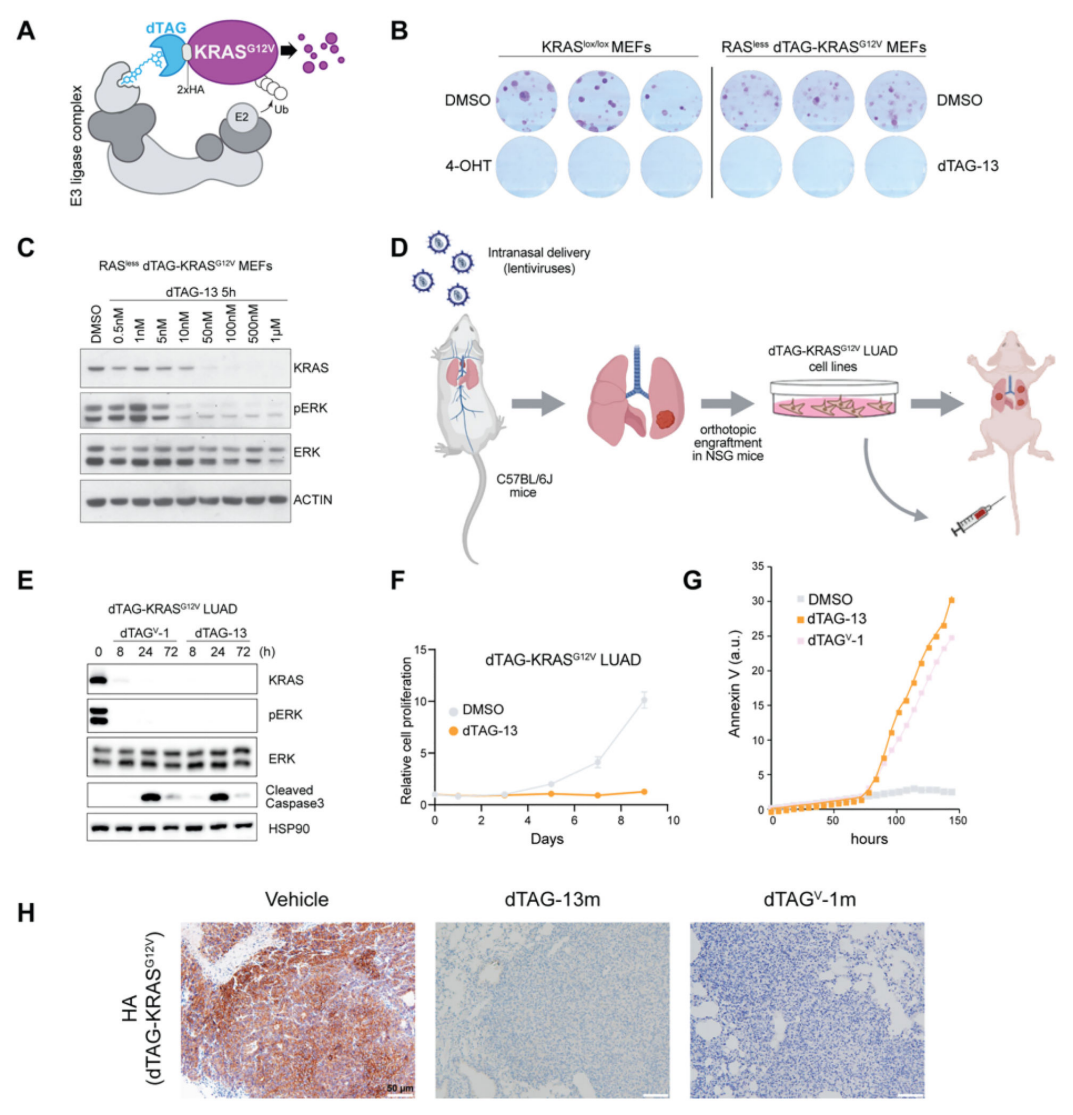
A novel LUAD mouse model driven by degradable KRAS^G12V^ enables PROTAC-dependent phenotyping *in vivo*. **A**, Depiction of the dTAG system. **B**, Colony forming assay of RAS^less^ MEFs without or with dTAG-KRAS^G12V^ expression. 4-OHT (0.6 µM); dTAG-13 = 0.3 µM. **C**, Degradation of dTAG-KRAS^G12V^ in RAS^less^ MEFs at the indicated doses and time. **D**, Depiction of the dTAG-KRAS^G12V^– driven LUAD model generation based on intranasal administration of lentiviruses carrying the dTAGged oncogene. **E**, Time-resolved degradation of dTAG-KRAS^G12V^ in LUAD cells established from tumors as depicted in (D) (dTAG-13 and dTAG^V^-1 = 0.5 µM). **F**, Growth curves of tumor-derived dTAG-KRAS^G12V^ LUAD lines (dTAG-13 = 0.5 µM). **G**, Time-resolved Annexin V staining upon degradation of dTAG-KRAS^G12V^ in LUAD cells (dTAG-13 and dTAG^V^-1 = 0.5 µM). **H**, Representative images of HA-stained (surrogate marker for dTAG-KRAS^G12V^) paraffin-embedded sections of LUADs from C57BL/6J mice treated with vehicle, dTAG-13m, or dTAG^V^-1m for 7 days at 40 mg/kg.

To explore the *in vivo* outcomes of acute KRAS^mut^ degradation in LUAD, we took advantage of the degradation TAG (dTAG) approach (Fig. 1A). In brief, this chemogenetic system uses a 12-kDa cytosolic prolyl isomerase engineered variant (FKBP12^F36V^) as a tag to make fusion proteins amenable to targeted degradation (39,40). This tag-based approach leverages the potency of standardized PROTACs such as dTAG-13 and dTAG^V^-1, which hijack the endogenous E3 ubiquitin ligases CRL4^CRBN^ and CRL2^VHL^, respectively (39,40). We focused on KRAS^G12V^, a variant with no selective, clinically approved inhibitors or PROTACs available. The C-terminal portion of KRAS contains the hypervariable region that cannot be modified as it is essential for KRAS trafficking and function. KRAS has been N-terminally fused to a variety of tags without any obvious biological alteration. Thus, we opted for the N-terminal dTAG fusion. The chimeric fusion also included two HA tags to facilitate detection (Fig. 1A). Of note, FKBP12^F36V^ and HA tags have been previously expressed *in vivo* with no immunogenicity reported (46–48). First, we validated *in cellulo* the ability of dTAG-KRAS^G12V^ to rescue the proliferation of cells devoid of all RAS isoforms (49,50), thus confirming the biological activity of the tagged oncoprotein and the overall cellular efficiency of the system (Fig. 1B, C and Supplementary Fig. S1A).

To establish mouse models amenable to targeted KRAS degradation, we innovated a LUAD-induction approach based on intranasal inhalation of dTAG-KRAS^G12V^-carrying lentiviruses into immunocompetent mice (Fig. 1D). Our approach confirmed the oncogenic activity of dTAG-KRAS^G12V^ by inducing the development of LUAD with an average latency of 10 months. Tumors were subsequently harvested and orthotopically implanted into the lungs of immunodeficient mice as this approach greatly enhances the development of primary cancer cell lines (45). We next established several cancer cell lines derived from independent lesions and evaluated the efficiency of the dTAG system *in cellulo* upon exposure to the dTAG-13 and dTAG^V^-1 PROTACs. As expected, acute degradation of KRAS^G12V^ resulted in downstream signaling blockade accompanied by concomitant proliferation arrest and apoptosis (Fig. 1E-G).

Tail vein injection of the established cell lines consistently enabled the generation of syngeneic mouse models of LUAD. To test the efficacy of dTAG-KRAS^G12V^ degradation *in vivo* we first optimized the scalable synthesis of two compatible PROTACs that we referred to as dTAG-13m and dTAG^V^-1m (Supplementary Fig. S1B; see Methods and Supplementary information for detailed synthetic and purification procedures). In brief, these PROTACs were prepared from the selective FKBP12^F36V^ ligand AP-1867 functionalized with a carboxylic acid group at the meta-position to facilitate the gram-scale synthesis. In addition, we designed and synthesized an additional PROTAC analogue (IGP002; Supplementary Fig. S1B) aimed at improving the metabolic stability and solubility of the formers. However, we observed concomitant cellular degradation of the endogenous FKBP12 (Supplementary Fig. S1C). This finding with IGP002 was particularly surprising given that it only differs from dTAG-13m in one atom. We assume that subtle differences in the ternary complex formation drive the lack of FKBP12^F36V^-selective degradation. Henceforth, we focused on dTAG-13m and dTAG^V^-1m, and observed efficient degradation *in vivo* by intraperitoneal administration at 40 mg kg^‐1^ (Fig. 1H).

Collectively, we established an efficient approach to study tumor-specific chemical modulation of KRAS^G12V^ protein abundance in LUAD syngeneic mice.

### Degradation of oncogenic KRAS inhibits downstream signaling resulting in efficient tumor regression *in vivo*

**Fig. 2:**
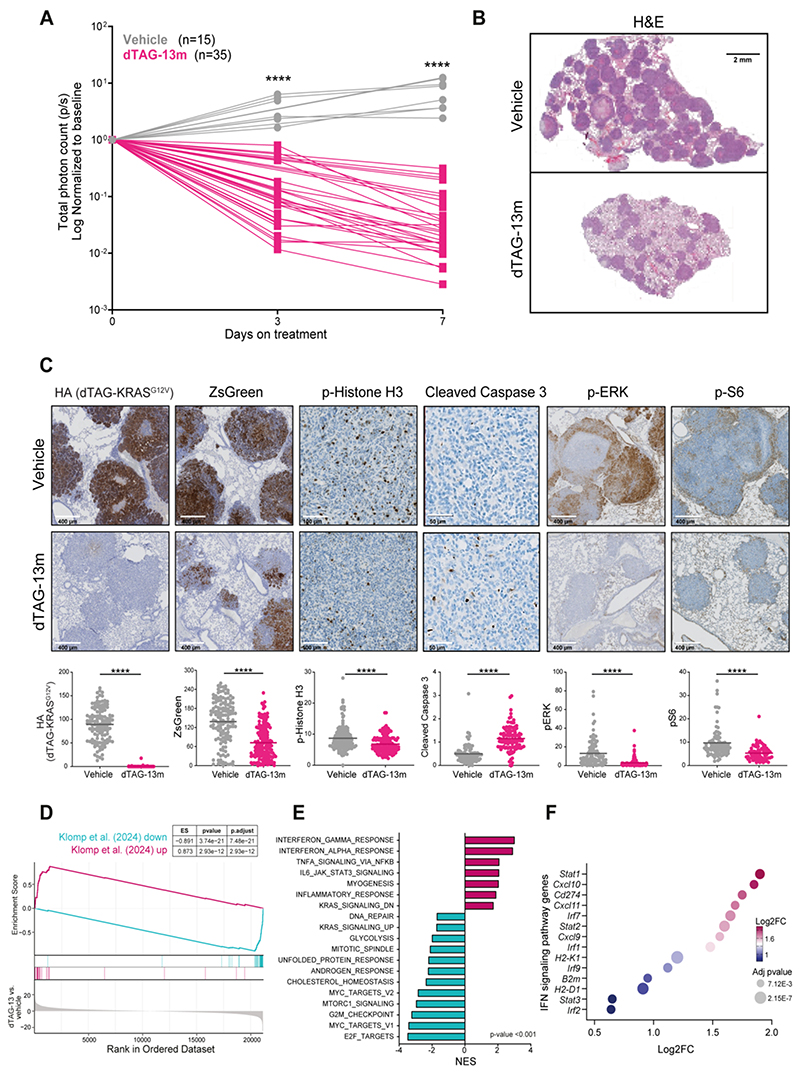
KRAS^G12V^ targeted degradation induces tumor regression associated with changes in cell- and non-cell-autonomous processes. **A**, Longitudinal quantification of LUAD tumor evolution by IVIS imaging (LUAD bioluminescence signal) in C57BL/6J mice treated daily with vehicle or dTAG-13m (40 mg/kg). Measurements were performed at days 3 and 7 post-treatment initiation. **B**, Representative images of hematoxylin and eosin (H&E)-stained, paraffin-embedded lung sections of tumor-bearing C57BL/6J mice treated daily with vehicle or dTAG-13m (40 mg/kg, day 7). **C**, Representative images of the indicated immunohistochemistry markers (upper panels) and the quantification (bottom graphs; H-score: see Methods) from tumor-containing lung sections of C57BL/6J mice treated with vehicle or dTAG-13m (40 mg/kg, day 7). **D**, Gene signature associated with KRAS inhibition (51) with enrichment scores of the corresponding up and downregulated genes differentially expressed in dTAG-13m vs. vehicle-treated FACS-sorted LUAD cells (ZsGreen+CD45-). ES: enrichment score. **E**, Hallmark gene set enrichment analysis (GSEA) with positive and negative enrichment scores from RNAseq data of 7-day dTAG-13m vs. vehicle treated FACS-sorted LUAD cells. NES: normalized enrichment score. **F**, Upregulated canonical IFN signaling pathway genes upon KRAS^G12V^ degradation. Statistical differences in A and C were analyzed using Mann-Whitney test. ****, p<0.0001. Enrichment scores and significance parameters in D, E and F were calculated using the GSEA software.

To assess the *in vivo* potential of a KRAS^G12V^ degradation-based therapy, we further modified the dTAG-KRAS^G12V^ cell lines described above by concomitantly introducing a luciferase reporter and fluorescent marker (ZsGreen) to allow both longitudinal assessment of tumor burden and purification of cancer cells upon tissue dissociation, respectively. To this end, we delivered the dTAG-KRAS^G12V^ LUAD cells to the lungs of isogenic C57BL/6J recipient mice via caudal vein injection. This procedure resulted in efficient engraftment followed by tumor development with a short latency period (3-4 weeks). In order to induce degradation of KRAS^G12V^ we utilized dTAG-13m given that most of the PROTACs currently in clinical trials rewire the E3 CRL4^CRBN^ (22). Daily intraperitoneal injection of dTAG-13m significantly suppressed tumor growth as early as 3 days post-treatment, assessed both by luminescence signal (Fig. 2A) and histopathological examination (Fig. 2B). Two independent cohorts were sacrificed following 3 and 7 days of continuous dTAG-13m treatment. Immunohistochemical detection of the ZsGreen cancer cell reporter confirmed the efficient tumor regression phenotype anticipated by the decline in the luminescence signal (Fig. 2C). Likewise, quantification of HA-tag immunostaining (a KRAS^G12V^ surrogate marker) demonstrated the acute degradation of the driver oncoprotein. Furthermore, immunostaining analyses of lung tumor sections showed that dTAG-13m treatment resulted in a significant reduction of proliferative cells and enhanced induction of apoptosis when compared to untreated counterparts (Fig. 2C). Oncoprotein degradation resulted in a marked downregulation of the ERK/MAPK and PI3K signaling pathways (Fig. 2C).

To gain deeper mechanistic insights, bulk RNA sequencing (RNAseq) was performed on LUAD cells (ZsGreen+/CD45– population) directly isolated upon dissociation and FACS separation of tumor-bearing lungs. The biological significance of KRAS^G12V^ degradation was reinforced by an overall transcriptional profile almost identical to a recently reported gene-signature obtained following chemical and genetic inhibition of the oncogene (51) (Fig. 2D). A more detailed scrutiny using gene set enrichment analysis (GSEA) identified 7 gene sets significantly enriched in dTAG-13m-treated tumor cells, including immune-related categories such as those associated with type I and type II interferon (IFN) alpha and gamma responses (Fig. 2E), previously reported in the context of oncogenic KRAS inhibition (52). Consistent with this finding, we observed increased expression of IFN target genes (Fig. 2F). This transcriptional reprogramming likely reflects multiple complementary mechanisms. Oncogenic KRAS signaling is known to repress IFN responses through inhibition of the JAK-STAT axis and MYC-dependent suppression of non-canonical IFN pathways (52–55). KRAS^G12V^ loss therefore might release this repression, reactivating IFN signaling. Additionally, 12 gene sets were found significantly downregulated when compared to untreated tumor cells with a remarkable presence of metabolic and proliferative related functions (Fig. 2E). Collectively these data indicate that targeted degradation of KRAS^G12V^ is a highly effective and rapid anti-tumoral approach.

### KRAS^G12V^ degradation induces a profound remodeling of the tumor microenvironment

**Fig. 3:**
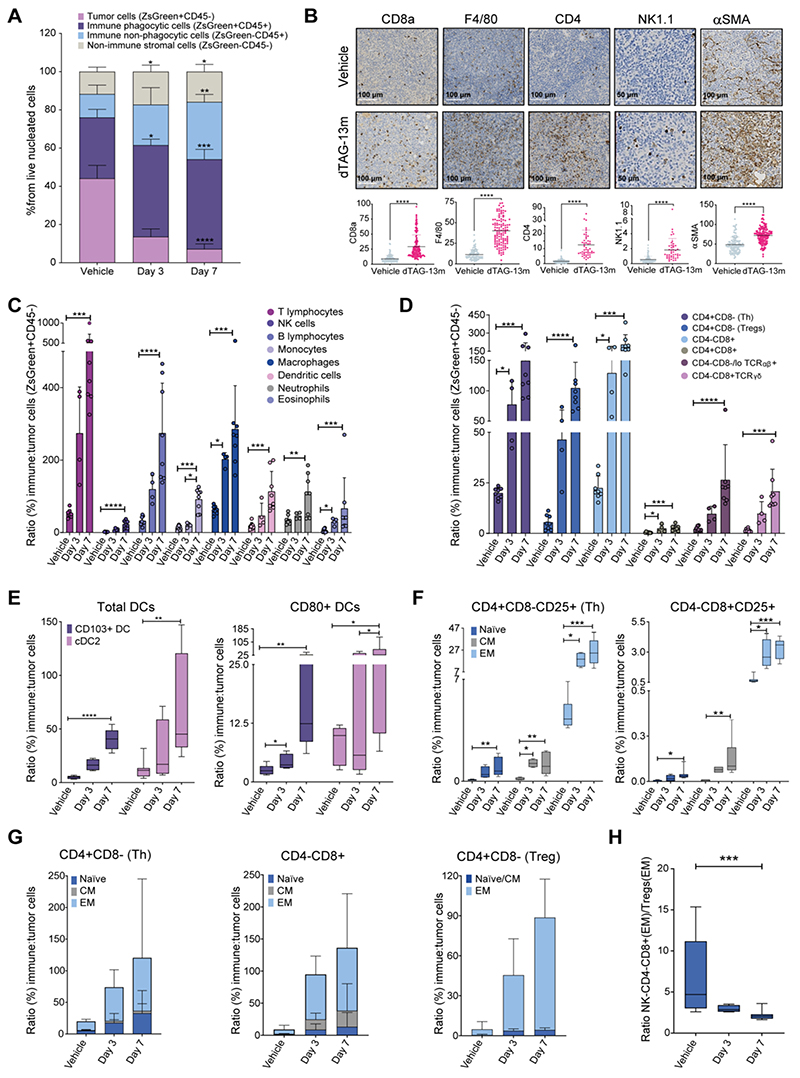
Alteration of the tumor microenvironment driven by KRAS^G12V^ degradation. **A**, Immune and non-immune cell population summary from the flow cytometry analysis following dissociation of tumor-bearing lungs. The relative frequency of the indicated cell populations for vehicle- and dTAG-13m-treated (40 mg/kg) C57BL/6J mice at 3 or 7 days post-treatment initiation are shown. **B**, Representative immunostainings of LUAD tumors using the indicated antibodies in both vehicle and dTAG-13m-treated cohorts (day 7 post-treatment initiation). The corresponding quantifications are indicated. **C**, Normalized abundance of the indicated immune cell populations in each of the treatment conditions. **D**, Relative abundance of the indicated T cell subtypes in response to vehicle or dTAG-13m treatment. **E-F**, Quantitative analysis of total and CD80+ dendritic cell subtypes (DCs) (E) and evaluation of T cell activation (CD25+) of CD4+CD8- (Th) and CD4-CD8+T cells (F) in vehicle, 3 day- and 7 day- dTAG-13m treated C57BL/6J mice. **G**, Maturation stage distribution for Naïve, Central Memory (CM) and Effector Memory (EM) T cell populations at the indicated time points in vehicle or dTAG-13m treated mice. CD4+CD8- (Th): day 3 vs. vehicle (EM) p=*; day 7 vs. vehicle (EM) p=***; day 7 vs. vehicle (naïve) p=*; day 7 vs. vehicle (CM) p=*. CD4-CD8+: day 3 vs. vehicle (EM) p=*; day 7 vs. vehicle (EM) p=***; day 7 vs. vehicle (naïve) p=*; day 7 vs. vehicle p=**. CD4+CD8- (Treg): day 7 vs. vehicle (EM) p=***; day 3 vs. vehicle (naïve/CM) p=**; day 7 vs. vehicle (naïve vs. CM) p=***. **H**, Evaluation of the combined contribution of cytotoxic cell populations (mature NK and CD8+CD4- T cells) compared to immunosuppressive regulatory T cell (Tregs) infiltrates in vehicle and dTAG-13m treated mice at 3 or 7 days post-treatment initiation. Statistical differences were analyzed using non-parametric Mann-Whitney test in (B) and non-parametric One-way ANOVA followed by False Discovery Rate multiple comparison tests in the rest of the panels. In panel (A) all the statistical differences correspond to the comparisons between treated and vehicle control conditions. *, p<0.05; **, 0.05<p<0.01; ****, p<0.0001. Data are indicated as mean ± SD. Except for panel B (n=4 in all cases), cohort sizes for all experiments were vehicle (n=8), dTAG-13m treated mice at 3 days (n=4) and 7 days (n=8).

The results presented above, highlighting the increased enrichment of several pro-inflammatory pathways following KRAS oncoprotein degradation, prompted us to investigate the tumor microenvironment (TME) composition. Dissociation and FACS analysis of tumor-bearing lungs from both dTAG-13m- and vehicle-treated C57BL/6J mice showed that tumor shrinkage coincided with profound TME remodeling, including increased stromal components and both phagocytic and non-phagocytic immune populations (Fig. 3A). Accordingly, immunohistochemical staining of tumor sections revealed increased infiltration of several immune populations and presence of cancer associated fibroblasts (Fig. 3B).

To more precisely quantify the immune-related changes we performed a comprehensive immunophenotypic characterization of the TME after 3 and 7 days of dTAG-13m treatment. Degradation of the KRAS oncoprotein significantly increased myeloid and lymphoid major lineages (Fig. 3C). Since various T-cell populations are pivotal in mediating cancer immune rejection, we focused our initial analyses on these compartments. Most notably, the relative abundance of all major T cell subtypes was significantly increased in a time-dependent manner upon oncogenic KRAS^G12V^ degradation (Fig. 3D), a process potentially linked to the transcriptional upregulation of the CXCL9, CXCL10, and CXCL11 chemokines and IFN-stimulated genes observed upon KRAS degradation (Fig. 2F) (56). This was accompanied by a parallel increase in the recruitment of dendritic cells including the CD80+ antigen-presenting population (Fig. 3E), suggesting enhanced T-cell priming potential. Indeed, the activated CD4-CD8+ and CD4+CD8-(Th) subsets appeared significantly amplified according to the CD25 activation marker (Fig. 3F). Moreover, KRAS^G12V^ degradation also promoted the acquisition of a differentiation phenotype with both memory and effector properties in the three major T cell populations analyzed (Fig. 3G). All these data suggest the acquisition of a robust adaptive cytotoxic response triggered by KRAS^G12V^ degradation. This effect was potentially enhanced both by increased MHC-I expression of LUAD cells (H2D1, H2K1 and B2M, Fig. 2F), making them more susceptible to cytotoxic CD4-CD8+ T cells, and by elevated numbers of innate cytotoxic natural killer (NK) cells (Fig. 3B-C). Altogether, these results highlight the prominent role played by oncogenic KRAS in the suppression of several anti-tumor immune responses that were significantly increased upon degrader treatment. Finally, the ratio between the sum of cytotoxic lymphoid CD8+CD4- and NK cells and the counteracting immunosuppressive Treg population was significantly diminished at day 7, suggesting a shift towards inflammation resolution likely due to the drastic reduction of the tumor burden induced by KRAS^G12V^ degradation (Fig. 3H).

### Short-term tumor regression upon KRAS^G12V^ degradation is primarily driven by cancer cell-autonomous mechanisms

**Fig. 4:**
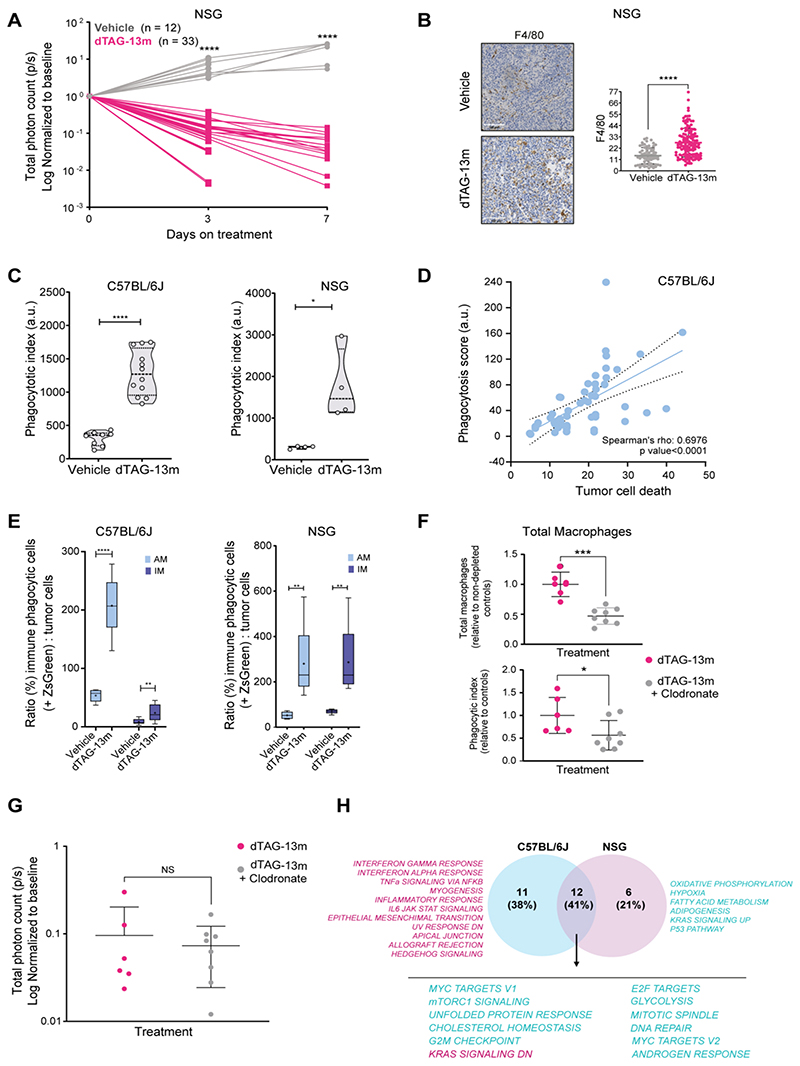
Adaptive and innate immunity are dispensable for the short-term tumor regression elicited by KRAS^G12V^-targeted degradation. **A**, Longitudinal quantification of LUAD tumor evolution by IVIS imaging (LUAD bioluminescence signal) in NSG mice treated daily with vehicle or dTAG-13m (40 mg/kg) at days 3 and 7 post-treatment initiation. **B**, Immunohistochemical scoring (H-score, see Methods) values for F4/80 expression (macrophages) in all lung samples from NSG mice subjected to the indicated treatment conditions (n=4 for both vehicle and dTAG-13m). Illustrative F4/80 immunostainings are shown. **C**, Comparative analysis (dTAG-13m vs. vehicle treatment) of the respective phagocytic indexes (see Methods) in C57BL/6J and NSG mice. C57BL/6J mice: n=8 (vehicle) and n=12 (dTAG-13m); NSG mice: n=4 (dTAG-13m) and n=4 (vehicle). **D**, Positive correlation between phagocytosis and tumor cell death in co-cultures of C57BL/6J bone marrow derived macrophages and tumor cell lines subjected or not to dTAG-13m treatment. Data from three independent experiments are shown. **E**, Relative abundance of AM (alveolar macrophages) and IM (interstitial macrophages) upon vehicle or dTAG-13m treatment in C57BL/6J and NSG mice. Sample size per strain as in (C). **F**, Relative abundance (upper) of total macrophages and their associated phagocytic activity (bottom) upon clodronate treatment in lungs from tumor-bearing C57BL/6J mice exposed to dTAG-13m. Sample size: n=6 (dTAG-13m) and n=8 (dTAG-13m+clodronate). **G**, Quantitative analysis of the bioluminescence signal in response to clodronate administration of dTAG-13m treated immunocompetent mouse cohorts. Sample size as in (F). **H**, Positively- (red) and negatively-enriched (green) biological processes in LUAD cells from dTAG-13m-treated tumor-bearing mice (C57BL/6J and NSG). Private as well as common differentially-expressed pathways are indicated. GSEAs of RNAseq data were applied using data from two independent experiments with each of the mouse strains (p.adjust <0.01). Statistical differences were analyzed using non-parametric Mann-Whitney test in all the panels. *, 0.05<p<0.01; **, 0.01<p<0.001; ***, 0.001<p<0.0001; ****, p<0.0001. Data are indicated as mean ± SD.

We went on to evaluate the role of both adaptive and innate immunity in the observed early tumor regression (up to 7 days of dTAG-13m treatment) described above. To this end, we replicated the orthotopic engraftment procedure using NSG mice, a strain characterized by lacking functional B, T and NK cells (57). Remarkably, tumor regression (measured by the decline in bioluminescence signal) occurred with similar kinetics as in the immunocompetent background (Fig. 4A and 2A). Accordingly, histological examination upon dTAG-13m administration showed a significant decrease in the tumor area, HA (dTAG-KRAS^G12V^), and ZsGreen positive LUAD cells (Supplementary Fig. S2A-B). Likewise, immunohistochemical analysis revealed an impairment of ERK/MAPK and PI3K signaling pathways accompanied by cleaved caspase 3 induction and compromised cell proliferation (Supplementary Fig. S2B). The phenotypes observed in the NSG mice rule out a key role of the adaptive immunity during the initial steps of tumor regression.

Next, we directly addressed the potential implication of innate immune responses in both mouse strains during the early phase of tumor regression (up to 7 days of dTAG-13m treatment). Considering the potential role of the phagocytic response as an anti-tumor mechanism (58), we took again advantage of the ZsGreen reporter expressed by the LUAD cells to perform a precise quantification of this process (ZsGreen+ phagocytes). Degradation of the KRAS oncoprotein greatly exacerbated the overall macrophage infiltration (Fig. 3B and 4B) as well as the phagocytic activity in both immunocompetent and immunocompromised mice (Fig. 4C). Similarly, co-culture experiments of three independent dTAG-KRAS^G12V^ mouse LUAD cell lines and bone-marrow derived macrophages from naïve immunocompetent mice showed a strong correlation between phagocytosis and dTAG-13m-induced tumor cell death (Fig. 4D and Supplementary Fig. S2C). Among the potential immune populations with phagocytic capacity, alveolar macrophages emerged as the most prominent cell type able to phagocyte tumor cells in the C57BL/6J background, while in NSG mice the interstitial macrophages also appeared implicated in this process (Fig. 4E and Supplementary Fig. S2D).

To evaluate the potential contribution of cancer-cell phagocytosis in the early anti-tumor response, we carried out clodronate-mediated depletion of macrophages. As expected, clodronate treatment caused a marked reduction of total macrophages reaching an average of 50% depletion (Fig. 4F, top) with a similar decay in the overall phagocytic activity (Fig. 4F, bottom). Importantly, this macrophage depletion did not alter the observed tumor regression upon KRAS^G12V^ degradation (Fig. 4G), suggesting that the phagocytic activity observed upon dTAG-13m treatment was likely a tissue clearance homeostatic response rather than a tumoricidal mechanism.

In summary, these results indicated that tumor cell-intrinsic processes have a major contribution to the short-term tumor regression triggered by KRAS^G12V^ degradation. Transcriptomic analysis of cancer cells isolated from control and dTAG-13m-treated lung tumors in both C57BL/6J and NSG mice revealed several private and common metabolic and proliferative processes that were strongly compromised upon oncoprotein degradation (Fig. 4H), suggesting these changes may underlie tumor regression.

To directly assess the contribution of cytotoxic T lymphocytes to tumor regression upon KRAS^G12V^ degradation, we conducted CD8+ T-cell depletion experiments in immunocompetent mice (44). To this end, we injected anti-mouse CD8a (Ly 2.2) 3 days prior to the initiation of dTAG-13m treatment in C57BL/6J mice. The control cohort was treated in parallel with an IgG2b isotype control and the procedure was maintained throughout the entire experiment. This protocol efficiently resulted in depletion of the CD8 pool as measured at end point (7 days post dTAG-13m treatment) by immunophenotyping of tumor cell suspensions (Supplementary Fig. S3A). Our results indicate that CD8+T cell immunodepletion resulted in a similar tumor regression rate when compared to the fully immunoproficient controls at both 3 and 7 days following the initiation of the dTAG-13m treatment. Indeed, the overall impact in tumor regression assessed after 7 days of treatment was 96.5% decrease in the absence of CD8 cells vs. 98.1% in the isotype-treated cohort (Supplementary Fig. S3B). The differences were even lower if assessed at an earlier time point (3 days). We prolonged the CD8+ immunodepletion experiment to assess the possibility that the adaptive immunity could extend overall survival by eliminating cells resistant to KRAS^G12V^ degradation (Supplementary Fig. S3C-E). Our results indicated that, despite efficient CD8+ immunodepletion, there was a negligible impact on overall survival when compared to the isotype-treated cohort, thus recapitulating the previous results obtained with the NSG mice (Fig. 4).

Additionally, we conducted this protocol in parallel using a 10-fold lower initial tumor burden to assess whether CD8+ contribution might manifest. When assessed in this experimental setting 3-days post dTAG-13m, we observed a statistically significant tumor-regression delay in the CD8-depleted animals (Supplementary Fig. S3B). However, assessment at 7 days post-treatment revealed no difference in tumor regression between cohorts, with similar average tumor burden regression rates when comparing the isotype and CD8-depleted cohorts.

When considered together, these results suggest a potential contribution of CD8+ cells in the kinetics of tumor regression following KRAS^G12V^ degradation, specifically in conditions of low-tumor burden. Yet, this immune-mediated clearance seems largely dispensable as similar regression rates are achieved with delayed kinetics in the absence of CD8+ cells, most likely mediated by cancer cell-intrinsic phenotypes.

### Resistance to KRAS^G12V^ degradation *in vivo* is mostly driven by dysregulation of the proteolytic machinery

**Fig. 5:**
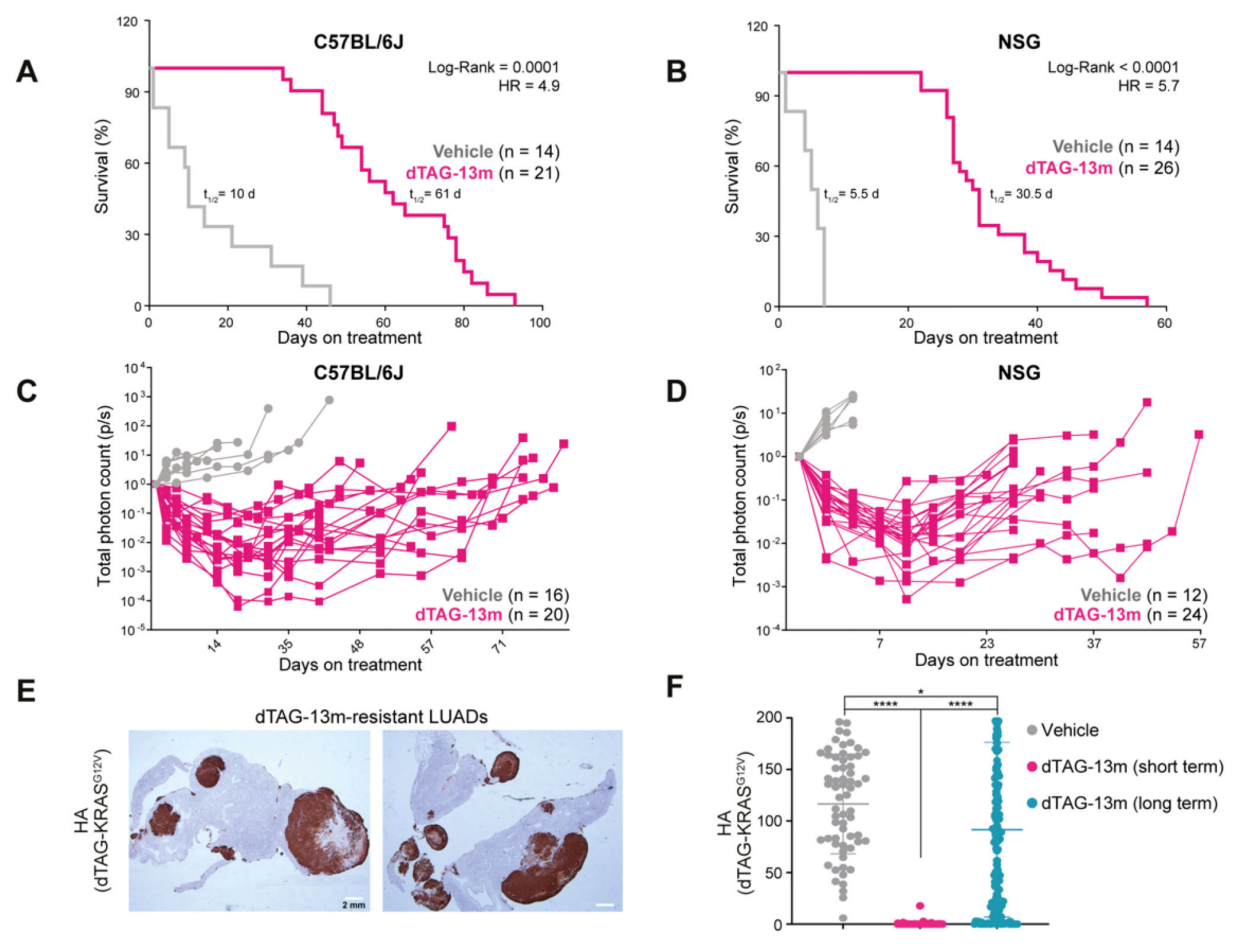
Prolonged dTAG-13m treatment *in vivo* leads to disease relapse accompanied by KRAS^G12V^ protein expression. **A-B**, Survival curves of C57BL/6J (A) or NSG (B) mice treated with vehicle or dTAG-13m (40 mg/kg) 4 days/week. The log rank test was used to evaluate survival differences between both groups, and the HR indicates an increased risk of LUAD related-death in the untreated group. t_1/2_ = mean survival time. **C-D**, Longitudinal assessment of dTAG-KRAS^G12V^-driven LUAD tumor evolution by IVIS imaging (LUAD bioluminescence signal in C57BL/6J (C) or NSG (D) mice treated with vehicle or dTAG-13m (40 mg/kg) 4 days/week. **E**, Representative images of HA (dTAG-KRAS^G12V^) paraffin-embedded sections of whole lung lobule sections including both normal lung parenchyma and LUADs from C57BL/6J mice treated with dTAG-13m (40 mg/kg) 4/days/week until humane end-point. **F**, Quantification of HA (dTAG-KRAS^G12V^) expression (H-score, see Methods) from paraffin-embedded whole lung lobule sections from C57BL/6J mice treated with vehicle or dTAG-13m (short term = 7 days; long term ≥ 45 days).

Patterns of PROTAC resistance *in vivo* remain largely unknown. Despite the rapid and efficient tumor regression elicited by KRAS^G12V^ degradation, in our long-term dTAG-13m treatments upon CD8+ T-cell depletion we eventually observed disease relapse (Supplementary Fig. S3C). To evaluate the molecular mechanisms underlying the emergence of resistance *in vivo* we followed both C57BL/6J and NSG cohorts on continuous dTAG-13m treatment until detecting tumor relapse (see Methods). dTAG-13m resistance appeared with accelerated kinetics in NSG mice resulting in shorter median time to progression and overall reduced survival when compared to C57BL/6J mice (Fig. 5A-D). The relative therapeutic benefit was nevertheless similar in immunocompetent and immunodeficient mice (Fig. 5A,B), as assessed by the median time to progression in treated vs. untreated animals (t_1/2_), thereby reinforcing the abovementioned message that KRAS^G12V^ degradation efficacy in LUAD seems largely independent of host immune status.

Immunohistochemical studies of the resistant tumors revealed that the vast majority of proliferative lesions retained KRAS^G12V^ protein expression (Fig. 5E,F). Thus, the tumor relapse observed during prolonged dTAG-13m treatment likely stems from defective PROTAC-induced KRAS^G12V^ proteolysis, indicating resistance mechanisms distinct from those reported upon KRAS inhibition.

**Fig. 6:**
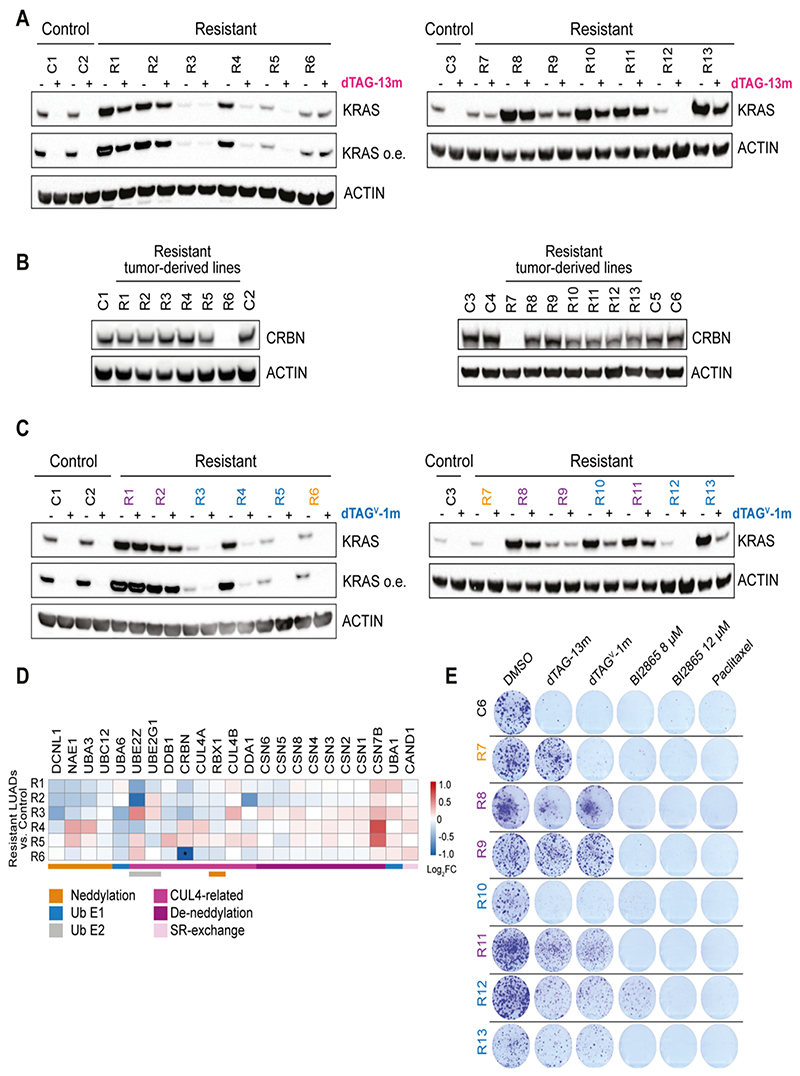
Resistance to KRAS degradation *in vivo* is mainly driven by diverse dysregulations of the proteolytic machinery. **A**, Degradation of dTAG-KRAS^G12V^ in LUAD murine cells treated with DMSO or dTAG-13m (0.3 µM, 24h). Cell lines were established from treatment-naïve (Controls) and dTAG-13m resistant LUAD tumors. Short and overexposed (o.e.) blots are shown. **B**, CRBN levels in the indicated cell lines. **C**, Degradation of dTAG-KRAS^G12V^ in LUAD murine cells treated with DMSO or dTAG^V^-1m (0.3 µM, 24h). Based on the assessments in Fig. 6A-C, in purple are cell lines with impaired degradation upon both dTAG-13m and dTAG^V^-1m, in blue cell lines responsive to dTAG^V^-1m-induced degradation, and in orange cell lines that are CRBN null and thus show lack of dTAG-13m-induced degradation. Short and overexposed (o.e.) blots are shown. **D**, Targeted proteomics via parallel reaction monitoring (PRM) of a selection of E3-related proteins. Log2FC (resistant LUADs vs. control) is shown in the heatmap (n=3). Functional classification is color-coded as indicated. Ub: ubiquitin; SR: substrate receptor. *CRBN in sample R6: lower than minimum color-coded scale. **E**, Colony forming assays in LUAD lines derived from dTAG-13m resistant or treatment-naïve tumors. dTAG-13m and dTAG^V^-1m = 0.5 µM; BI-2865 (panKRAS inhibitor) = 8 µM and 12 µM. Paclitaxel = 0.5 µM (minimum dose to affect control cells).

To ensure a comprehensive representation of the *in vivo* resistance landscape, 13 cell lines were established from dTAG-13m-resistant tumors for further phenotyping, together with 6 control lines derived from treatment-naïve animals. Immunoblotting confirmed that dTAG-13m-driven KRAS^G12V^ degradation was compromised in most of the resistant lines compared to control cells (Fig. 6A). Only 3 out of the 13 lines retained substantial dTAG-13m-induced KRAS^G12V^ degradation (R4, R5, and R12). Relative to the controls, the dTAG-KRAS^G12V^ basal levels in the resistant lines were heterogeneous (similar, elevated, or reduced) (Fig. 6A).

We then focused on understanding the mechanisms driving the lack of targeted degradation. To discard potential technical issues related to the chemogenetic system, we first sequenced the dTAG-KRAS^G12V^ fusion in the cell lines established from the resistant LUAD tumors revealing lack of on-target secondary mutations that could impair dTAG-13m binding or induced ubiquitination (Supplementary Table S4). Efflux pump overexpression has been reported as a mechanism of resistance to PROTACs (59,60). However, these models were sensitive to paclitaxel, a common substrate of efflux pumps (61), and efflux pump inhibitors did not restore dTAG-13m-induced degradation of KRAS (Supplementary Fig. S4A,B).

The loss of function of the E3 hijacked by degraders is the most common resistance mechanism *in cellulo* (33,34,62–65). To our surprise, only 2 of the tumor-resistant cell lines (R6 and R7) showed lack of CRBN expression (Fig. 6B), suggesting that the *in vivo* mechanisms of PROTAC resistance might differ from previous *in cellulo* observations (33,34,62–65). We treated our dTAG-13m resistant models with dTAG^V^-1m, which recruits CRL2^VHL^ instead of CRL4^CRBN^. In addition to the 2 cell lines without CRBN (Fig. 6C, in orange), 6 additional lines showed complete or partial sensitivity to this alternative PROTAC (Fig. 6C, in blue), suggesting that, in this group, the lack of KRAS degradation was driven by factors related to the CRL4^CRBN^ complex. Interestingly, around half of the dTAG-13m resistant cell lines were also resistant to dTAG^V^-1m (Fig. 6C, in purple). These results suggested frequent dysregulation of proteolytic machinery upstream or downstream of the E3 ligases as a driver of resistance *in vivo*.

To query changes in the protein plasticity of E3-related factors, we defined a panel of functionally-relevant proteins related to the Cullin-RING ligase (CRL) family of ubiquitin E3s, and performed targeted proteomics via parallel reaction monitoring (PRM) in cell lines representative of the aforementioned phenotypes. This included R1 and R2 as resistant to dTAG-13m and dTAG^V^-1m, R3 as resistant to dTAG-13m and partially sensitive to dTAG^V^-1m, R4 and R5 as examples of considerable responders to dTAG-13m and to dTAG^V^-1m, and R6 as a CRBN-null for comparison. We captured a trend in the tumor-derived resistant cell lines R1-R3 towards dampening the levels of important activation machinery common to CRL4^CRBN^ and CRL2^VHL^ (Fig. 6D). For instance, downregulation of DCNL1 and NAE1, both part of the neddylation cascade to activate CRLs, was observed and might contribute to the defective targeted degradation of KRAS (Fig. 6D). We also mined our RNAseq data to search for potential coding point mutations that might compromise ligase activity in this panel of E3-related genes. In addition to the resistant cell lines, control and dTAG-13m-resistant tumors from immunocompetent and immunodeficient mice were analyzed. We only found one point mutation (50% of reads, which would be equivalent to a heterozygote tumor) corresponding to a single-nucleotide insertion predicted to cause a frameshift in the *Nae1* gene (Supplementary Fig. S4C and Supplementary Table S5). Overall, we could not find any other point mutations predicted to affect E3 activity in the resistant cell lines or tumors, suggesting the observed dysregulation at the protein level as the most probable contribution towards resistance (Fig. 6D). Beyond neddylation, intrigued by the UBE2Z downregulation showed by PRM analysis for some cell lines, the levels of this ubiquitin-priming E2 enzyme were assessed by western blotting in the remaining lines (R7-R13). Interestingly, most of these resistant cell lines showed downregulation of UBE2Z (Supplementary Fig. S4D). Intriguingly, UBE2Z and the alternative E1 ubiquitin-activating enzyme UBA6 with which it preferentially cooperates have been previously nominated as modifiers of degrader sensitivity in our previous work using CRISPR screens (66). Given that UBA1 is the principal source of activated ubiquitin in our cells (~90%), the UBA6-UBE2Z axis might act as a complementary ubiquitin supplier to sustain degrader-induced ubiquitination on top of the normal cellular needs. Finally, baseline chymotrypsin-like proteasome activity was comparable across control and resistant lines and remained pharmacologically responsive (Supplementary Fig. S4E). This finding is consistent with our previous results, suggesting that PROTAC resistance *in vivo* might arise in most cases from heterogeneous, concomitant dysregulation of components upstream of the proteasome rather than from global proteasome impairment (Fig. 6D and Supplementary Fig. S4E).

Beyond KRAS^G12V^ degradation impairment, higher proliferation capacity was observed in dTAG-13m resistant cell lines when compared to treated controls (Fig. 6E and Supplementary Fig. S5A). In this regard, 3 cell lines (R4, R5, and R12) preserved considerable targeted degradation capacity (Fig. 6A,C) but showed substantial proliferation (Fig. 6E), suggesting adaptive or acquired alterations that bypass KRAS^G12V^ absence instead of proteolysis impairment. We explored potential bypass mutations in expressed genes by performing SNV/INDEL calling in the resistant lines, prioritizing variants with high-predicted functional impact. First, we focused specifically on those bypass mutations described in the context of resistance to KRAS inhibitors (20). Only one of the resistant lines (R5) harbored a gain-of-function mutation (particularly in *Pik3ca*), which could help explain why growth inhibition was not totally impaired upon dTAG^V^-1m treatment although KRAS was substantially degraded (Supplementary Fig. S5B). To further explore other potential bypass mutations across the resistant lines, we performed transcriptome‐wide SNV/INDEL calling (Supplementary Fig. S5C-D). Validating our initial UPS-focused variant calling in tumors and lines (Supplementary Fig. S4C), no UPS-related mutations were found in the resistant lines. Thus, further emphasizing our main conclusion that impaired KRAS degradation *in vivo* is predominantly shaped by heterogeneous concomitant protein changes in proteostasis components (Fig. 6D). Some loss-of-function mutations found in lines R4 and R12 (e.g., *Zfp609, Tigd2*, and *Dsn1, Rbbp6* and *Mmp17*) suggest potential lineage-specific adaptive routes that may help preserve proliferation under partial KRAS loss. Showcasing another hybrid resistance mechanism, the R10 line had impaired KRAS degradation (Fig. 6A,C) but proliferation was compromised despite being established from a dTAG-13m resistant tumor (Fig. 6E). Further analyses will be required to fully understand these resistance-driving alterations captured *in vivo*.

Finally, given that the KRAS oncoprotein was still expressed, we decided to test whether our degradation-resistant LUAD models were sensitive to KRAS inhibitors. dTAG-13m resistant cells, including the dTAG^V^-1m cross-resistant cell lines, were sensitive to the panKRAS inhibitor BI-2865 (Fig. 6E), showcasing their maintained dependency on the driver oncoprotein. These results support the exploration of different, orthogonal pharmacology against the same target to defeat resistance.

In summary, impaired KRAS^G12V^ degradation upon PROTAC treatment was the predominant resistance pattern observed *in vivo*. Mechanistically, our studies suggest that resistance *in vivo* mainly occurs as a consequence of heterogeneous defects in E3-dependent protein degradation rather than KRAS bypass adaptations.

## Discussion

Given the limited duration of the clinical responses to FDA-approved KRAS inhibitors, finding alternative and/or combination therapies is a pressing medical need. In this context, the development of KRAS PROTACs, with some already under early clinical evaluation, could represent a significant breakthrough in the treatment of KRAS-driven cancers. Yet, there is currently very limited information regarding the mechanistic phenotypes brought about by oncogenic KRAS targeted degradation *in vivo* and no data concerning the potential resistance mechanisms.

We focused on modeling targeted degradation of KRAS^G12V^ in LUAD using the dTAG system. Leveraging this chemogenetic strategy, and upon optimization of the gram-scale synthesis of the PROTAC dTAG-13m, we enabled *in vivo* mechanistic and follow-up studies that could inform the efficacy of the first KRAS PROTACs suitable for first-in-human studies (67). Furthermore, our work demonstrates the potential of similar PROTAC-tag mouse models to study, validate, and mechanistically profile the pharmacological degradation of drug targets *in vivo* prior to investing in the development of direct degraders. Overall, our preclinical findings provide proof-of-principle of KRAS^G12V^ degradation as a therapeutic strategy in LUAD, potentially leading to rapid tumor regression that appears to be primarily mediated by cancer cell-autonomous mechanisms. We hypothesize that dampened pro-survival and essential metabolic functions, together with severely reduced downstream signaling (Fig. 4H), are the major contributors to early tumor regression. The substantial metabolic alterations brought about by KRAS^G12V^ degradation at early time points suggest that this is a promising avenue of research to potentially extend therapeutic responses by leveraging metabolism-related pharmacologic interventions.

Using a conditional dTAG-KRAS^G12V^ knock-in mouse model and neutralizing CD8 antibodies, Li and colleagues recently reported that the anti-tumor response upon oncogene degradation is partly dependent on CD8+ T cells (44). While we cannot exclude that methodological and/or overall tumor burden differences may explain the discrepancy, our results using NSG mice –which lack mature T, B and NK cells (57)– suggest that the adaptive immune response is functionally dispensable to elicit tumor regression (Fig. 3 and 4A). These observations were reinforced by the lack of notable differences in tumor-regression in C57BL/6J mice following depletion of CD8+ T cells or macrophages (Supplementary Fig. S3 and Fig. 3F,G). The enhanced immune infiltration and activation upon dTAG-13m treatment (this work and Li et al. (44)) likely arise from both intrinsic and extrinsic cues. Oncogenic KRAS signaling suppresses canonical IFN responses through multiple mechanisms, including inhibition of JAK-STAT signaling (53,54). KRAS^G12V^ degradation therefore likely releases this suppression, allowing reactivation of IFN signaling pathways (Fig. 4H) resulting in immune infiltration. The concomitant cell cycle arrest, metabolic collapse, and apoptosis upon KRAS loss (Fig. 2C-F) may further amplify IFN responses by generating cytosolic nucleic acids that engage derepressed innate immune sensing machinery. KRAS^G12C^ inhibition has also been shown to activate non-canonical IFN signaling by relieving a MAPK/MYC-dependent repression (52) and, therefore, IFN suppression may result from combined MYC and KRAS activity (55). The relative contribution of these regulatory programs to IFN reactivation upon KRAS^G12V^ degradation remains unclear. It is tempting to speculate that a putative crosstalk with the adaptive immunity is contributing to this response. Adaptive immunity may further shape this response as IFN transcriptional activation in cancer cells was consistently observed in tumors from immunocompetent mice upon KRAS^G12V^ degradation (Fig. 4H), whereas the same approach in NSG mice or in cultured cells led to more variable IFN responses. Together, both intrinsic and extrinsic regulation of the IFN program likely cooperate to drive robust antitumor immunity by enhancing T cell infiltration, cytotoxicity, and antigen presentation.

Therefore, the contribution of cell-extrinsic processes to the long-term evolution of low tumor burden conditions (Supplementary Fig. S3) and their therapeutic potential cannot be fully excluded. Indeed, immunotherapy enhances responses to KRAS inhibition in preclinical LUAD models (56). Irrespectively of the regulatory circuitry involved, our transcriptomic and immunohistological data revealed increased PD-L1 in dTAG-13m-treated LUADs (Supplementary Fig. S6A-B and Fig. 2F ‐*Cd274* gene), supporting combined KRAS degradation and immunotherapy. Although this appealing strategy remains untested in LUAD, its use in preclinical colorectal models has shown synergy with immunotherapy (68). Furthermore, KRAS-derived neo-peptides are efficient immunological targets (69). PROTACs targeting mutant KRAS may further enhance anti-tumor immunity by generating neo-antigens upon proteasomal degradation. Moreover, some of these potential PROTAC-induced neo-antigens, especially mutant KRAS peptides, could be harnessed for adoptive therapies like CAR-T cells. Interestingly, a recent manuscript by Benton et al. partially exploited this rationale detailing a proof of concept with promising results (70). While some of these notions will require additional experimental support, our observations suggest synergy between KRAS degradation and tailored immunotherapy to achieve durable responses.

Currently, the patterns of PROTAC resistance *in vivo* remain largely unknown. We and others have mapped in cultured cells how E3 ligases and their regulators shape sensitivity to degraders (33,34,62,63,71). Similar E3-centered resistance has been observed clinically with molecular glue degraders such as IMiDs (72,73) largely differing from the target-centric resistance associated with traditional inhibitors. Our *in vivo* studies revealed that long-term treatment (>45 days on dTAG-13m) eventually resulted in disease relapse due mainly to the lack of efficient KRAS^G12V^ protein degradation. Interestingly, our results consistently pointed to heterogeneous yet convergent dysregulation of E3/UPS machinery as the dominant mechanism of PROTAC resistance *in vivo*. Remarkably, only 2 out of the 13 cell lines established from dTAG-13m-resistant tumors exhibited CRBN loss (Fig. 6B) and no tumor or cell line harbored mutations predicted to have an impact on CRBN function (Supplementary Fig. S5C), which differentiates *in vivo* resistance from the studies in cells, in which CRBN loss of function is the prevailing resistance mechanism observed with CRL4^CRBN^-dependent degraders (33–35). Another surprising finding was that 5 of the 13 dTAG-13m-resistant cell lines showed cross-resistance to both dTAG-13m (a CRL4^CRBN^-based PROTAC) and dTAG^V^-1m (a CRL2^VHL^-based PROTAC), challenging the widespread assumption that sequential PROTAC co-opting of different E3s might lead to longer responses. In summary, our findings add to the emerging understanding of adaptive responses to KRAS-targeted therapies that, whereas resistance to KRAS inhibitors commonly involves amplification or reactivation of the MAPK pathway (20,31), resistance to KRAS degradation *in vivo* appears to arise primarily from heterogenous, concomitant dysfunction within the E3/UPS machinery. Mechanistically, PROTAC efficacy depends on coordinated activity across multiple UPS components; hence, even partial dysfunction at different nodes can compromise degradation efficiency.

Our preclinical study is based on tag-based PROTACs, thus, direct KRAS degraders might have divergent resistance mechanisms and/or toxicity profiles, including different pharmacokinetic and pharmacodynamic properties that might result in distinct tumor accumulation and/or impaired pathway coverage. Nevertheless, we hypothesize that the resistance patterns we observed with dTAG-13m are expected to be similar for PROTACs currently in clinical trials against a variety of targets, given that most of them also hijack the E3 CRL4^CRBN^ via similar thalidomide-inspired ligands.

When compared to the panKRAS inhibitor BI-2493 (17), KRAS^G12V^ degradation resulted in a more profound signaling inhibition, increased immune infiltration, and overall enhanced tumor regression (Supplementary Fig. S6). The distinct degree of target inhibition may trigger different selective pressures leading to molecularly diverse resistance mechanisms. Nevertheless, despite these mechanistic differences, previous work together with the results presented here suggest that KRAS dependency is an overarching feature in the disease relapse setting in LUAD, irrespectively of the therapeutic strategy. Our findings suggest a valuable opportunity to explore on-target combination therapies with different pharmacological modalities. Indeed, the use of a KRAS inhibitor was able to overcome degrader resistance *in cellulo*, suggesting that the sequential use of PROTACs and inhibitors could become a promising strategy to achieve more sustained therapeutic responses in KRAS-driven LUAD. Ongoing research with engineered mouse models will further elucidate the systemic effects triggered by KRAS degradation and help refine therapeutic strategies.

## Supplementary Material

1

2

3

4

5

6

7

8

9

10

11

12

13

## Data Availability

The data generated in this study are available within the article and its Supplementary files. RNAseq data has been deposited at GEO (RRID:SCR_005012) with the identifier GSE301303. The mass spectrometry proteomics data has been deposited at PRIDE (RRID:SCR_003411) with the identifier PXD058734. All other raw data generated in this study are available upon request from the corresponding authors.

## Statement of Significance

KRAS degradation induces rapid regression of lung adenocarcinoma tumors mainly via cancer cell-intrinsic mechanisms, offering a complementary strategy to target KRAS given the short duration of clinical responses to inhibitors.
