# Supplementary material for: Targeted KRASG12V Degradation in vivo Elicits Lung Adenocarcinoma Regression with Subsequent Relapse from Dysregulated Proteolysis
Source: Cancer Res. Author manuscript; Available in PMC 2026 Jun 13. (PMC7619155; doi:10.1158/0008-5472.CAN-25-5172)
Supplement: 1 [file EMS214174-supplement-1.pdf]

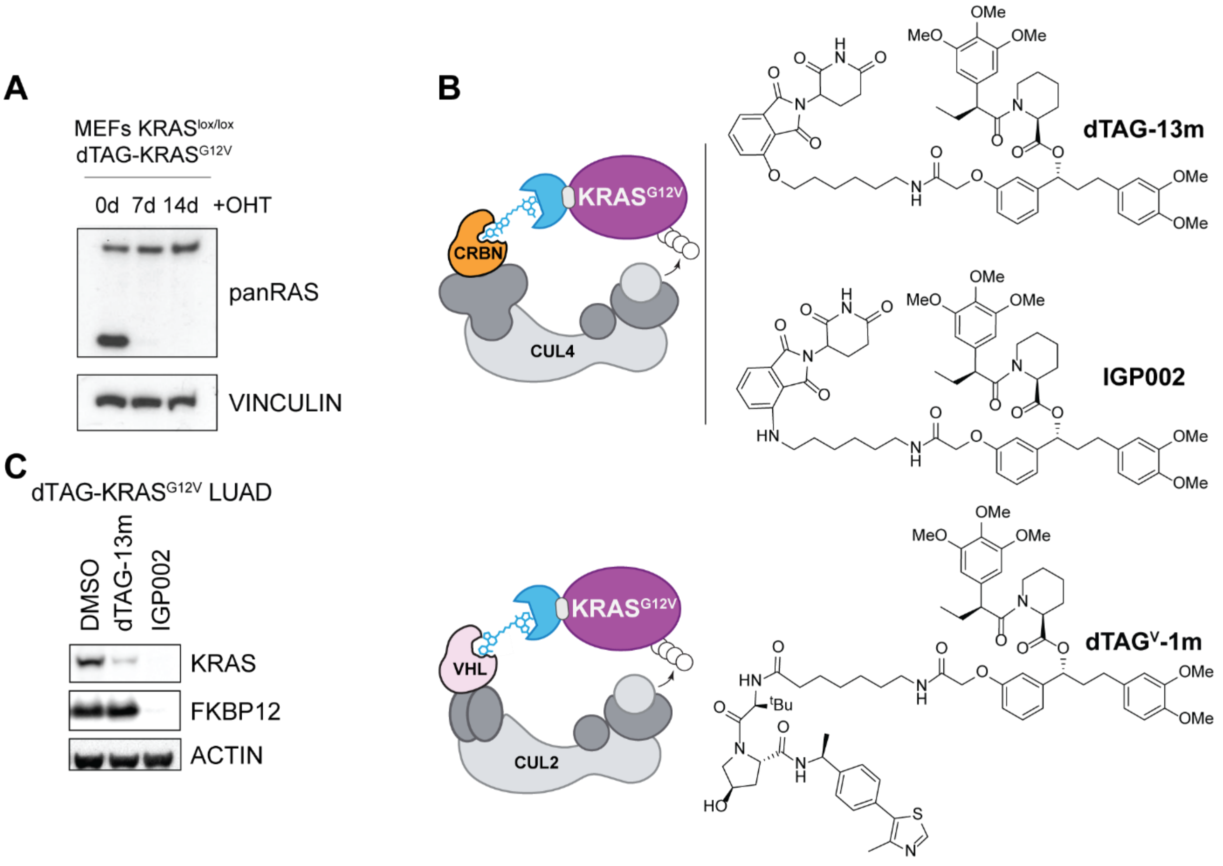

Supplementary Fig. S1. Selection of PROTACs compatible with the dTAG-KRAS^{G12V} LUAD model. **A,** RAS^{less} MEFs with dTAG-KRAS^{G12V} expression generated from H/NRAS$^{-/-}$ KRAS$^{lox/lox}$ MEFs. (OHT: 0.6 µM). **B,** Structures of the dTAG PROTACs tested in (C) and depictions of the E3 recruited. IGP002 was synthesized aiming to improve the metabolic stability and solubility of the previously reported PROTAC dTAG-13m. **C,** Degradation of dTAG-KRAS^{G12V} and endogenous FKBP12 in LUAD murine cells with DMSO or the
