# Supplementary material for: Targeted KRASG12V Degradation in vivo Elicits Lung Adenocarcinoma Regression with Subsequent Relapse from Dysregulated Proteolysis
Source: Cancer Res. Author manuscript; Available in PMC 2026 Jun 13. (PMC7619155; doi:10.1158/0008-5472.CAN-25-5172)
Supplement: 2 [file EMS214174-supplement-2.pdf]

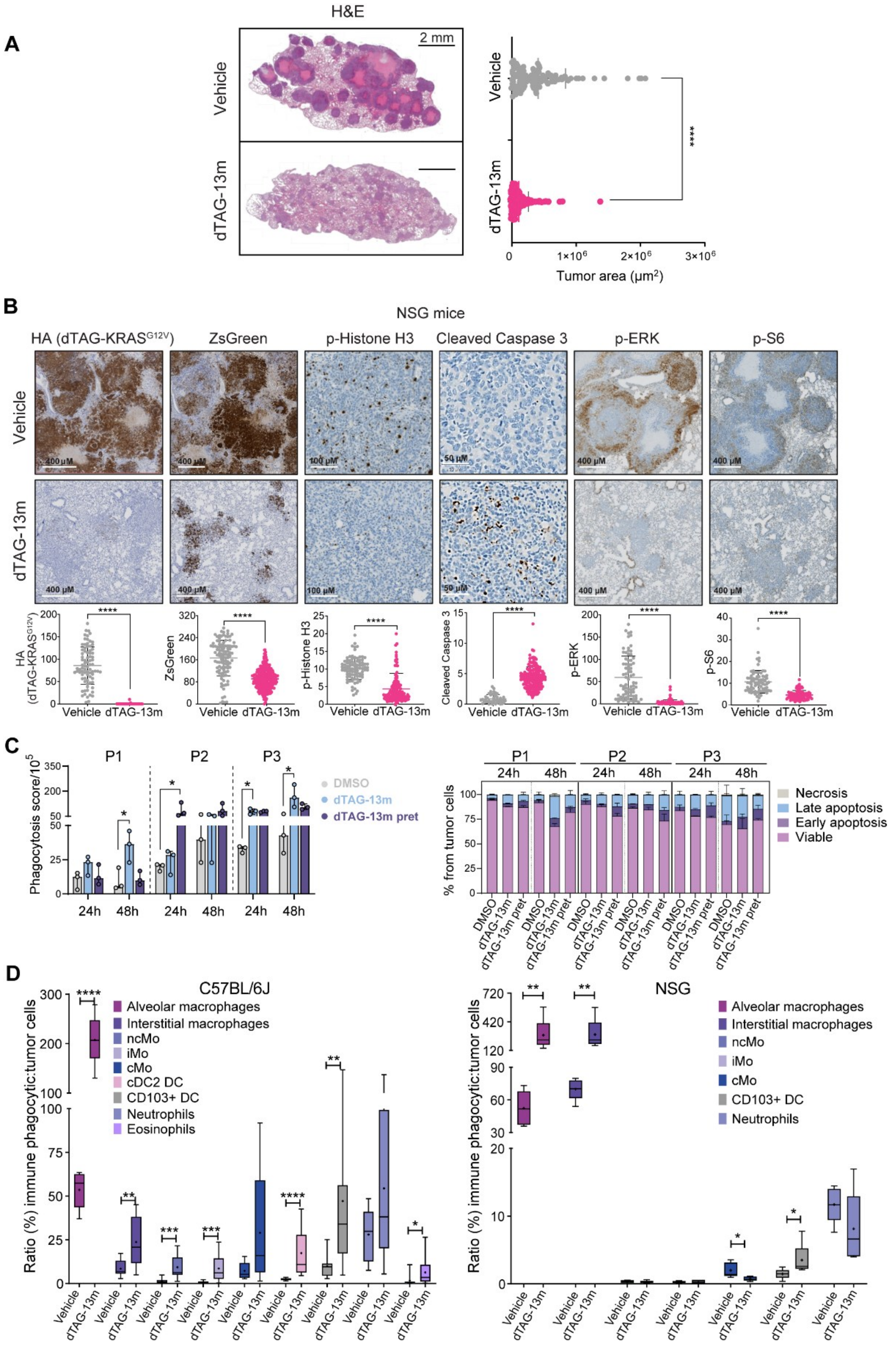

Supplementary Fig. S2. The anti-tumor response elicited by KRAS^{G12V} degradation in immunocompromised NSG mice closely resembles the effects observed in immunocompetent C57BL/6J mice. A, Representative example of H&E-stained sections of tumor-bearing lungs collected from NSG mice at day 7 post-treatment initiation with vehicle or dTAG-13m (40 mg/kg) together with the respective tumor area quantifications (right). **B,** Representative images of the indicated immunohistochemistry markers (upper panels) and the quantification (bottom graphs; H-score, see Methods) from tumors of NSG mice treated with vehicle or dTAG-13m (40 mg/kg) at day 7 post-treatment

initiation. Quantification data correspond to individual lung tumors derived from all the animals included in each of the mouse cohorts (vehicle (n=4) and dTAG-13m (n=4) treated). **C,** Quantification analysis of the phagocytic index (left) in vehicle or dTAG-13m treated co-cultures of bone marrow-derived macrophages from naïve immunocompetent mice and 3 independent dTAG-KRAS^{G12V} LUAD cell lines (P1-P3) at the indicated time points. Cell death analysis of each of the LUAD cell lines is also shown (right). The pre-treated condition corresponds to LUAD cell lines exposed to dTAG-13m 2 days before co-cultures were established. **D,** Relative abundance of phagocytic cells in both treated and untreated C57BL/6J (n=12 and n=8, respectively) and NSG (n=4 in both settings) mice. AM (Alveolar Macrophages), IM (Interstitial Macrophages), ncMo (non-classical Monocytes), iMo (intermediate Monocytes), cMo (classical Monocytes), DCs (Dendritic Cells). Statistical differences were analyzed using non-parametric Mann-Whitney test, except for panel C, where a non-parametric one-way ANOVA test was used. *, $0.05<p<0.01$; **, $0.01<p<0.001$; ***, $0.001<p<0.0001$; ****, $p< 0.0001$. Data are indicated as mean ± SD.
