# Supplementary material for: Targeted KRASG12V Degradation in vivo Elicits Lung Adenocarcinoma Regression with Subsequent Relapse from Dysregulated Proteolysis
Source: Cancer Res. Author manuscript; Available in PMC 2026 Jun 13. (PMC7619155; doi:10.1158/0008-5472.CAN-25-5172)
Supplement: 3 [file EMS214174-supplement-3.pdf]

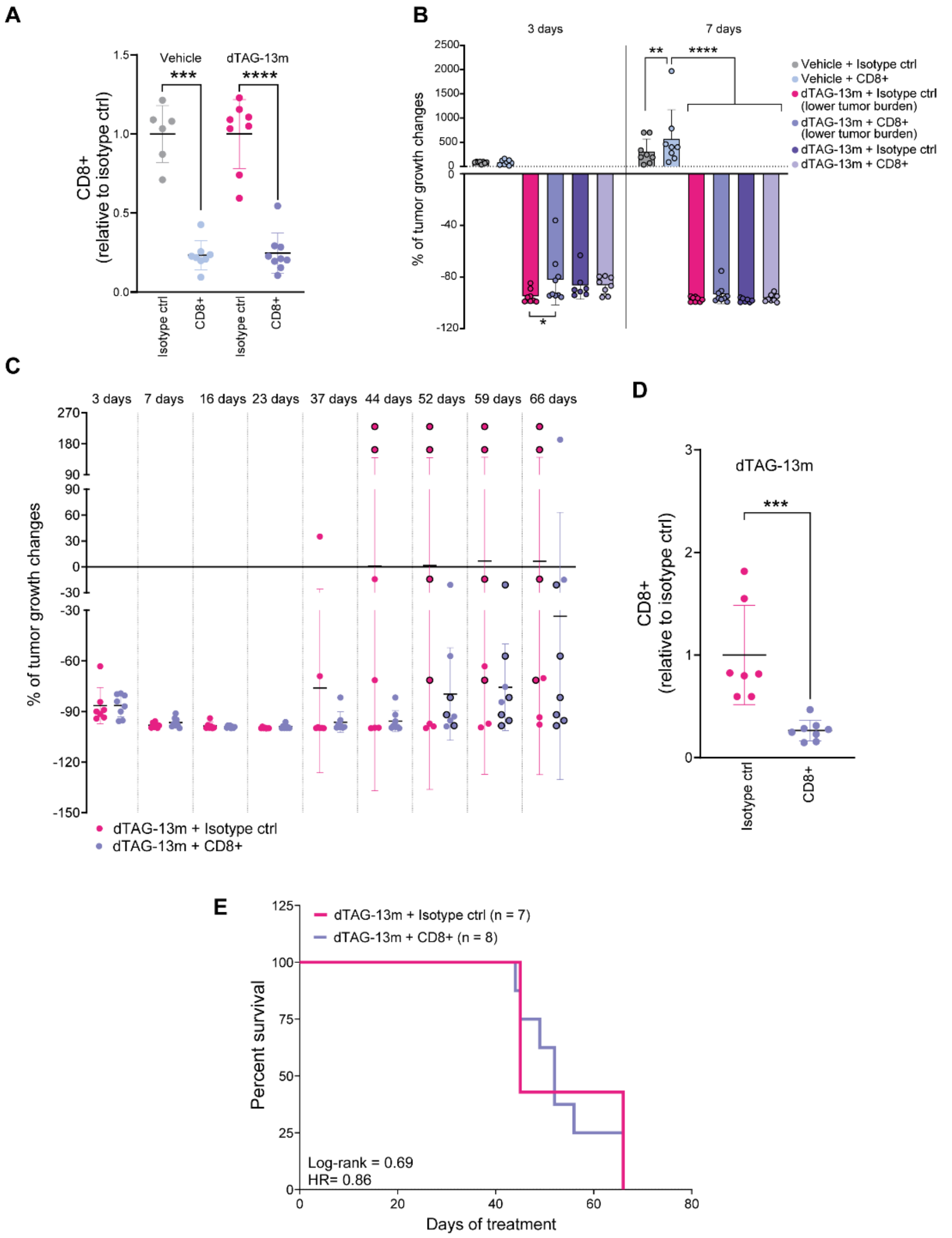

Supplementary Fig. S3. Effect of CD8+T cell immunodepletion in LUAD tumor regression upon KRAS^{G12V} degradation. A, Efficiency of CD8^{+} T-cell depletion in C57BL/6J tumor-bearing mice at 7-days post-treatment initiation. **B,** Tumor growth changes at 3- and 7-days post-treatment initiation in control and CD8-depleted mice. Results are shown using the standard protocol in this study (10^8 p/s average bioluminescence signal per animal at treatment initiation) as well as low tumor burden protocol (10^7 p/s average bioluminescence signal per animal at treatment initiation). **C,** IVIS analysis of tumor progression (standard bioluminescence protocol) in control and CD8-depleted mice treated with dTAG-13m for up to 66 days. Black-outlined dots represent deceased animals. **D,** Assessment of CD8^{+} T cell depletion efficiency at the humane endpoint. **E,** Kaplan-Meier curve displaying the impact of the indicated experimental conditions on mouse survival (standard bioluminescence protocol). The log-rank test was used to evaluate survival differences between groups, and the HR indicates the risk of LUAD related-death in the non-depleted group. Statistical differences were analyzed using non-parametric Mann-Whitney test in (A, C and D) and non-parametric one-way ANOVA followed by FDR correction for multiple comparison in (B) (statistical significance detected between dTAG-13m treated groups with lower tumor burden at day 3 was determined using a non-parametric Mann-Whitney test). *, $p < 0.05$; **, $0.05 < p < 0.01$; ****, $p < 0.0001$. Data are indicated as the mean ± SD. Depending on the assay, 6 to 9 animals per condition were examined. Antibodies for CD8+T cell depletion were administered at 200 µg every 3-4 days, and dTAG-13m was given at 40 mg/kg daily during the first week and 4 days per week thereafter throughout the duration of the experiment.
