# Supplementary material for: Targeted KRASG12V Degradation in vivo Elicits Lung Adenocarcinoma Regression with Subsequent Relapse from Dysregulated Proteolysis
Source: Cancer Res. Author manuscript; Available in PMC 2026 Jun 13. (PMC7619155; doi:10.1158/0008-5472.CAN-25-5172)
Supplement: 4 [file EMS214174-supplement-4.pdf]

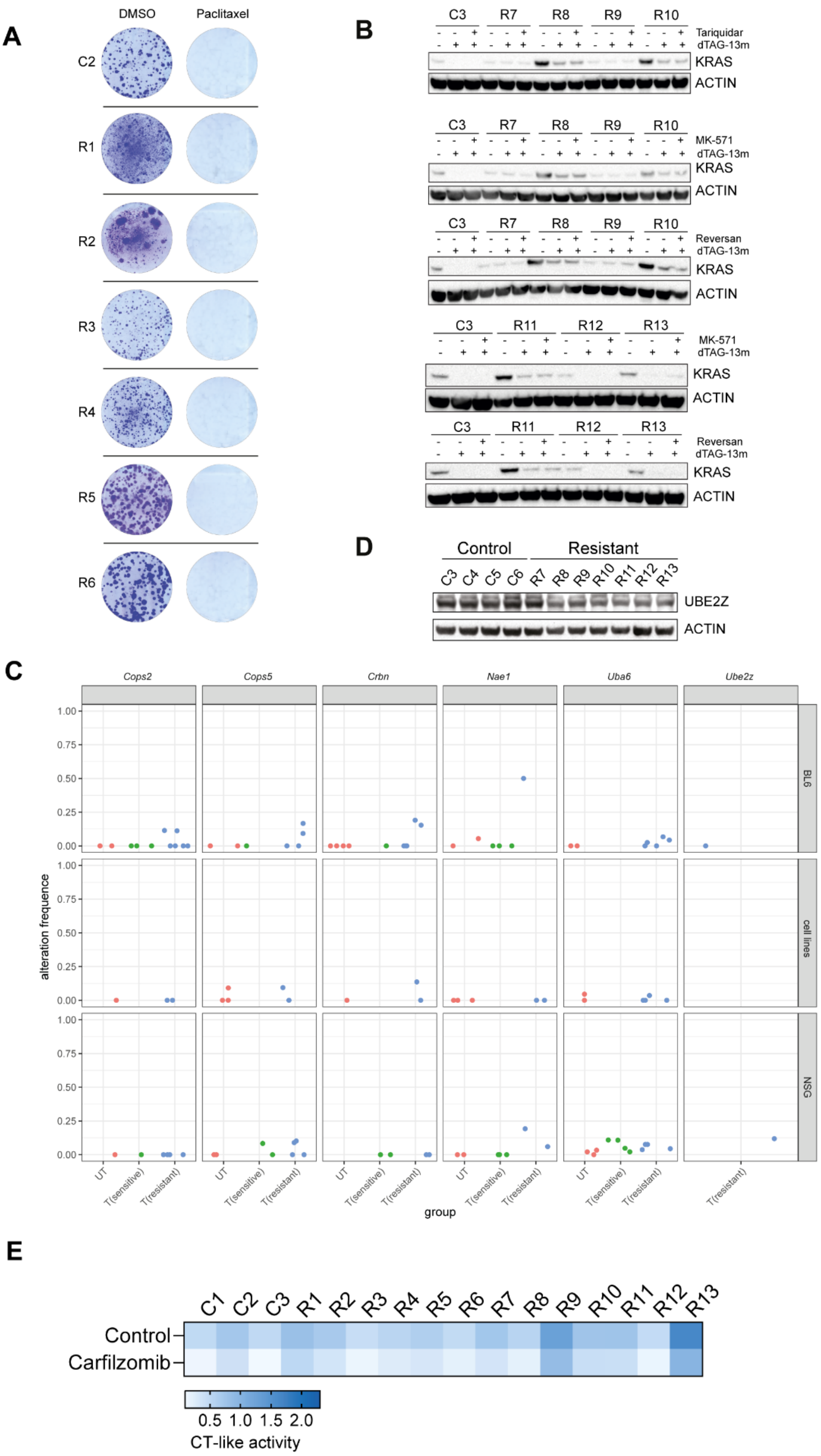


**Supplementary Fig. S4. LUAD resistance to targeted degradation *in vivo* is not mainly driven by efflux pump overactivation or point mutations in E3 machinery. A,** Colony forming assay in cells derived from dTAG-13m sensitive and resistant LUADs. Paclitaxel = 0.5 μM (minimum dose to affect control cells). **B,** Degradation of dTAG-KRAS$^{G12V}$ in tumor cells derived from dTAG-13m sensitive and resistant LUADs, treated with the indicated compounds. dTAG-13m = 0.3 μM, 24h; Tariquidar (ABCB1

inhibitor), MK-571 (ABCC1 inhibitor), Reversan (ABCB1 and ABCC1 inhibitor) = 1 μM, 24h. ACTIN is shown as loading control. **C,** Frequency of alternative allele in SNPs or INDELs predicted to have a high or moderate impact in protein function. SNP calling with GATK Haplotype caller and annotation with SnpEff (see Methods). BL6: tumors from immunocompetent mice (C57BL/6J). NSG: tumors from immunocompromised mice. UT: untreated (red). T(sensitive): dTAG-13m sensitive tumors (green). T(resistant): dTAG-13m resistant tumors or derived cell lines (blue). BL6 UT tumors n=4, sensitive n=4, resistant n=8. Cell lines UT n=3, resistant n=6 (R1-R6). NSG UT tumors n=4, sensitive n=4, resistant n=6. Only samples with variants with a minimum coverage of 10 reads are shown. **D,** UBE2Z levels in the indicated murine LUAD cell lines. ACTIN is shown as loading control. **E,** Chymotrypsin-like (CT-like) proteasome activity across murine LUAD cell lines. Heatmap of basal CT-like activity in control and carfilzomib-treated cells, showing that proteasome activity is present and can be effectively inhibited. Carfilzomib 1 μM 30 min. Color represents the mean; n=3 independent treatments.

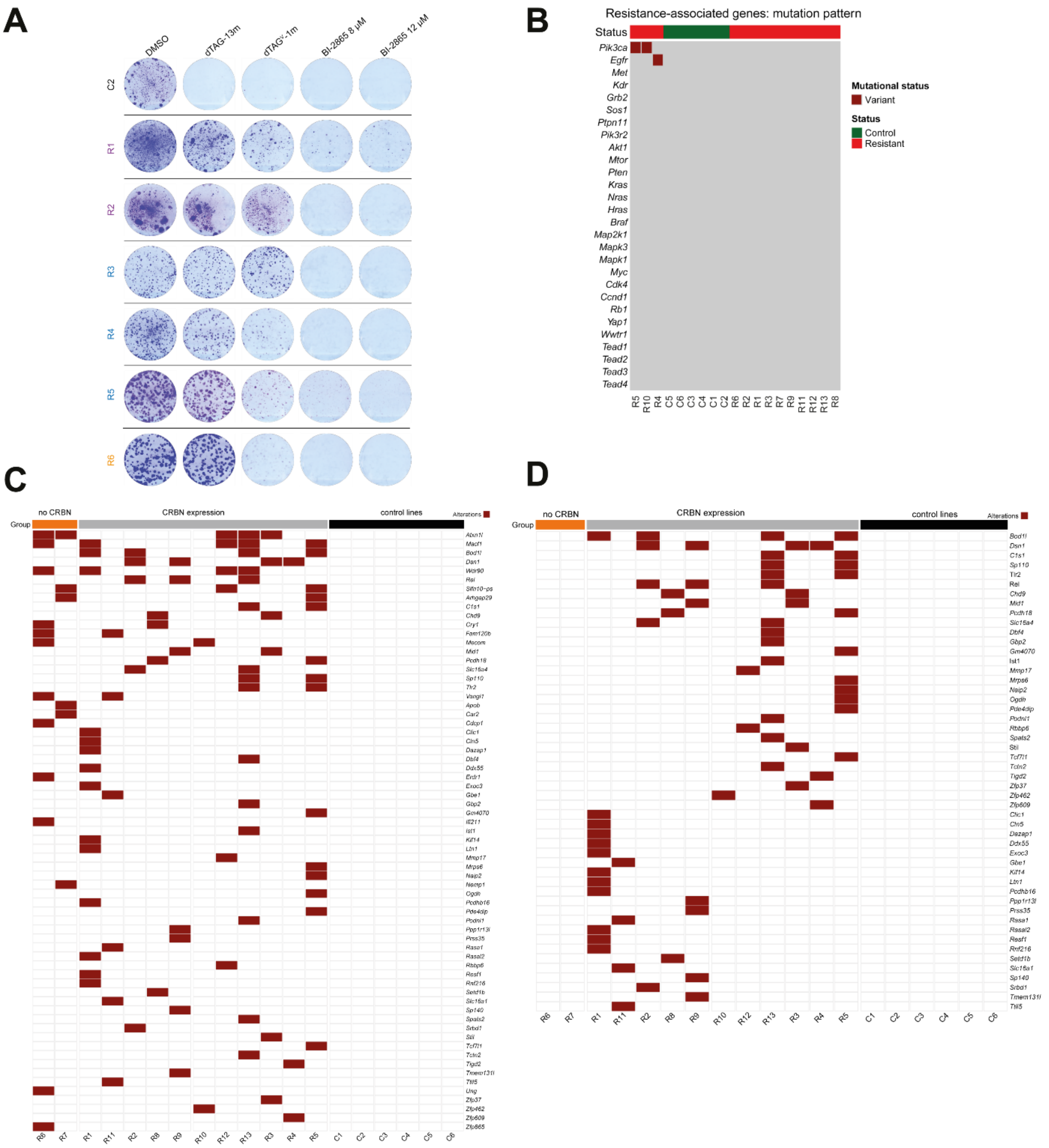


**Supplementary Fig. S5. LUAD tumor-derived cell lines resistant to PROTAC-induced KRAS$^{G12V}$ degradation are sensitive to KRAS inhibitors. A**, Colony forming assay in cells derived from dTAG-13m sensitive and resistant LUADs. dTAG-13m and dTAG$^{V}$-1m = 0.5 µM; Paclitaxel = 0.5 µM (minimum dose to affect control cells). BI-2865 (panKRAS inhibitor) = 8 µM and 12 µM. Based on the assessments in Fig. 6A-C, in purple are cell lines with impaired degradation upon dTAG-13m and dTAG$^{V}$-1m, in blue cell lines responsive to dTAG$^{V}$-1m-induced degradation, and in orange cell lines that are CRBN null and thus show lack of dTAG-13m-induced degradation. DMSOs are the same than in Supplementary S4D as paclitaxel was tested at the same time than the indicated compounds. **B**, Mutation pattern of typical bypass pathways related to KRAS inhibitors in LUAD lines resistant to KRAS$^{G12V}$ degradation. Pik3ca variants: R5 line (Resistant): c.328_330delGAA p.Glu110del, predicted gain of function; R10 line (Resistant): c.1037T>C p.Val346Ala, unknown significance. Egfr variant: R4 line (Resistant): c.2010G>T p.Met670Ile, unknown significance. Predicted impact of the variant according to oncokb.org. LUAD lines derived from resistant tumors (R1-R13). **C, D**, Mutation pattern in LUAD lines resistant to KRAS$^{G12V}$ degradation (beyond the resistance-associated set shown in B). SNPs or INDELs predicted to have a high or moderate impact in protein function. SNP calling with GATK Haplotype caller and annotation with SnpEff (see Methods). (C) Mutation pattern of the 13 resistant lines (genes with mutations in control

lines were filtered out). (D) Mutation pattern of the 11 lines in which CRBN is expressed (genes with mutations in control lines and lines R6 and R7 were filtered out).

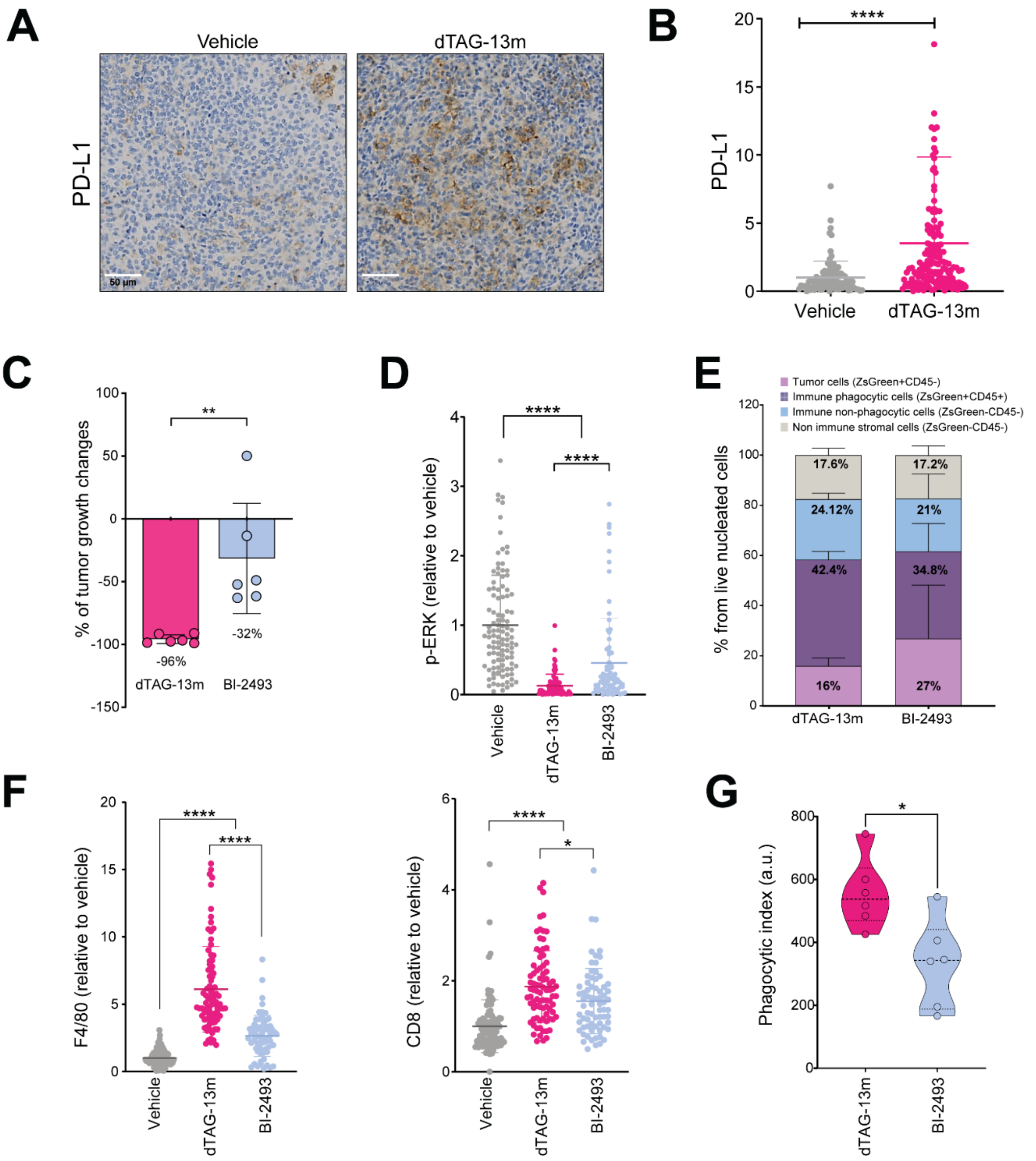


**Supplementary Fig. S6. KRAS degradation vs. inhibition. A**, Representative immunohistochemical staining of PD-L1 in lung tumors from vehicle and dTAG-13m-treated C57BL/6J mice are shown. **B**, Quantification analysis (H-score, see Methods) of the PD-L1 levels from vehicle and dTAG-13m-treated mice (n=3). **C**, Tumor growth change in C57BL/6J mice treated with dTAG-13m or BI-2493 (panKRAS inhibitor). **D**, Immunohistochemistry quantification of p-ERK (H-score: see Methods) from tumors of C57BL/6J mice treated with vehicle, dTAG-13m or BI-2493. **E**, Distribution of tumor, immune phagocytic, immune non-phagocytic and stromal cells among live lung cells after treatment with dTAG-13m or BI-2493. **F**, Immunohistochemistry quantification of F4/F80 and CD8 (H-score: see Methods) from tumors of C57BL/6J mice treated with vehicle, dTAG-13m, or BI-2493. **G**, Phagocytic index in the lung upon dTAG-13m or BI-2493 treatment. All the analysis were performed 6 days after treatment initiation, following daily (dTAG-13m; 40mg/kg) or twice daily (BI-2493; 60 mg/kg) dosing schedules. Statistical differences were analyzed using non-parametric Mann-Whitney test in A, C and E, and non-parametric One-way ANOVA followed by False Discovery Rate multiple comparison tests in the rest of the panels.

*, $0.05<p<0.01$; **, $0.01<p<0.001$; ***, $0.001<p<0.0001$; ****, $p<0.0001$. Data are indicated as the mean ± SD. In panels C-G 6 animals per condition were examined.
