# Supplementary material for: Targeted KRASG12V Degradation in vivo Elicits Lung Adenocarcinoma Regression with Subsequent Relapse from Dysregulated Proteolysis
Source: Cancer Res. Author manuscript; Available in PMC 2026 Jun 13. (PMC7619155; doi:10.1158/0008-5472.CAN-25-5172)
Supplement: 5 [file EMS214174-supplement-5.pdf]

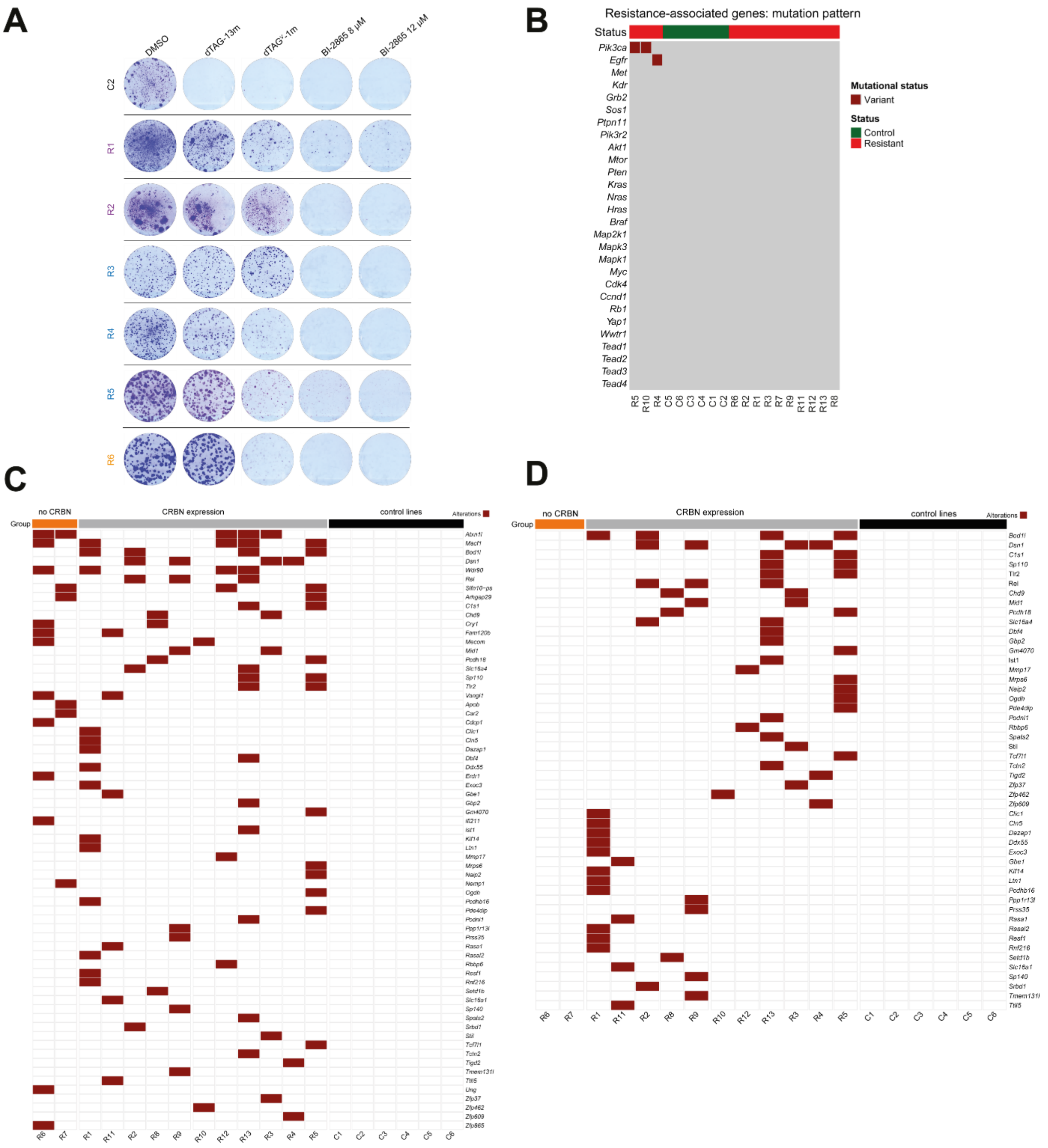

Supplementary Fig. S5. LUAD tumor-derived cell lines resistant to PROTAC-induced KRAS^{G12V} degradation are sensitive to KRAS inhibitors. A, Colony forming assay in cells derived from dTAG-13m sensitive and resistant LUADs. dTAG-13m and dTAG^{V}-1m = 0.5 μM; Paclitaxel = 0.5 μM (minimum dose to affect control cells). BI-2865 (panKRAS inhibitor) = 8 μM and 12 μM. Based on the assessments in Fig. 6A-C, in purple are cell lines with impaired degradation upon dTAG-13m and dTAG^{V}-1m, in blue cell lines responsive to dTAG^{V}-1m-induced degradation, and in orange cell lines that are CRBN null and thus show lack of dTAG-13m-induced degradation. DMSOs are the same than in Supplementary S4D as paclitaxel was tested at the same time than the indicated compounds. **B**, Mutation pattern of typical bypass pathways related to KRAS inhibitors in LUAD lines resistant to KRAS^{G12V} degradation. Pik3ca variants: R5 line (Resistant): c.328_330delGAA p.Glu110del, predicted gain of function; R10 line (Resistant): c.1037T>C p.Val346Ala, unknown significance. Egfr variant: R4 line (Resistant): c.2010G>T p.Met670Ile, unknown significance. Predicted impact of the variant according to oncokb.org. LUAD lines derived from resistant tumors (R1-R13). **C, D**, Mutation pattern in LUAD lines resistant to KRAS^{G12V} degradation (beyond the resistance-associated set shown in B). SNPs or INDELs predicted to have a high or moderate impact in protein function. SNP calling with GATK Haplotype caller and annotation with

SnpEff (see Methods). (C) Mutation pattern of the 13 resistant lines (genes with mutations in control lines were filtered out). (D) Mutation pattern of the 11 lines in which CRBN is expressed (genes with mutations in control lines and lines R6 and R7 were filtered out).
