# Supplementary material for: Targeted KRASG12V Degradation in vivo Elicits Lung Adenocarcinoma Regression with Subsequent Relapse from Dysregulated Proteolysis
Source: Cancer Res. Author manuscript; Available in PMC 2026 Jun 13. (PMC7619155; doi:10.1158/0008-5472.CAN-25-5172)
Supplement: 6 [file EMS214174-supplement-6.pdf]

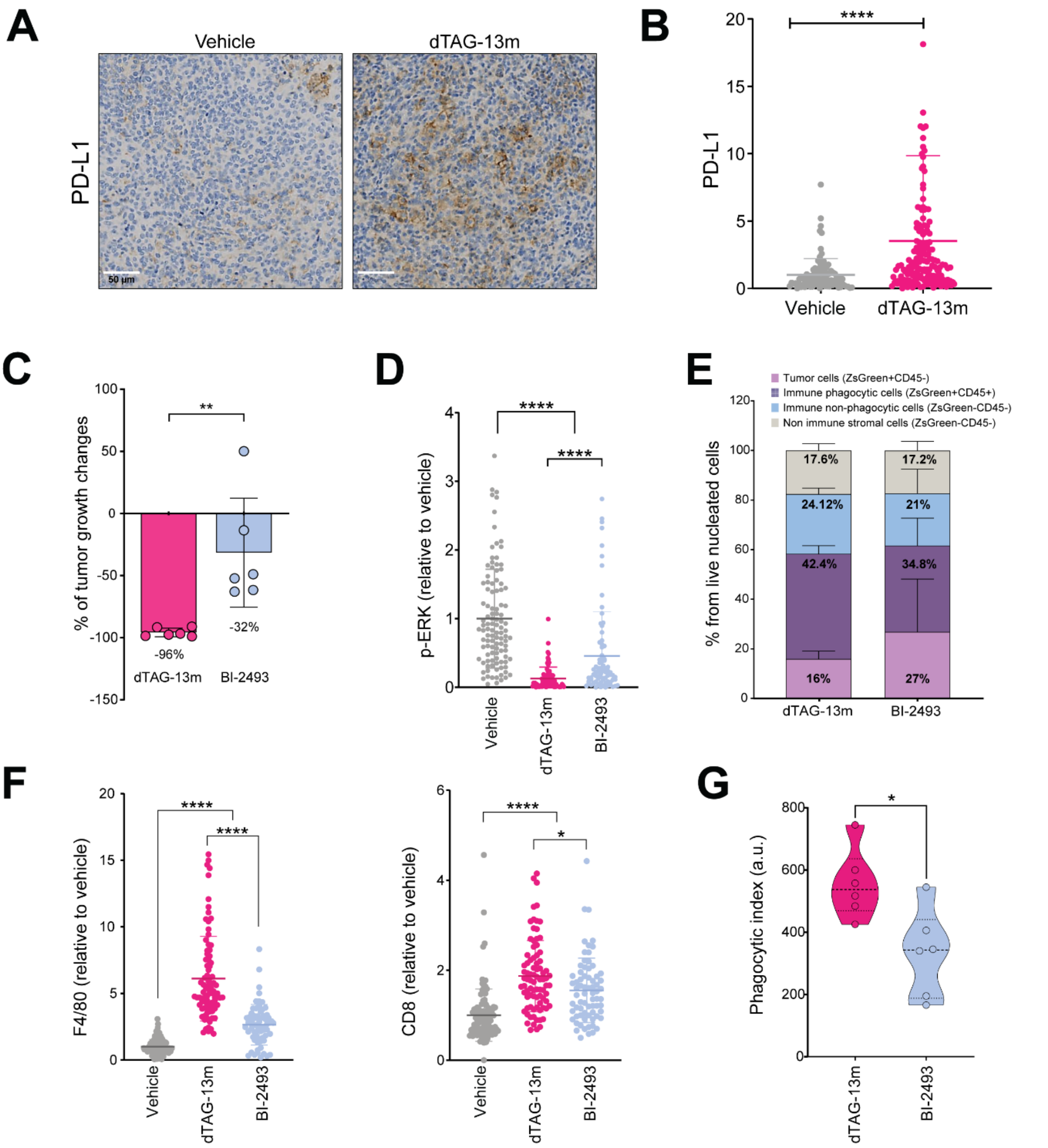

Supplementary Fig. S6. KRAS degradation vs. inhibition. A, Representative immunohistochemical staining of PD-L1 in lung tumors from vehicle and dTAG-13m-treated C57BL/6J mice are shown. **B**, Quantification analysis (H-score, see Methods) of the PD-L1 levels from vehicle and dTAG-13m-treated mice (n=3). **C**, Tumor growth change in C57BL/6J mice treated with dTAG-13m or BI-2493 (panKRAS inhibitor). **D**, Immunohistochemistry quantification of p-ERK (H-score: see Methods) from tumors of C57BL/6J mice treated with vehicle, dTAG-13m or BI-2493. **E**, Distribution of tumor, immune phagocytic, immune non-phagocytic and stromal cells among live lung cells after treatment with dTAG-13m or BI-2493. **F**, Immunohistochemistry quantification of F4/F80 and CD8 (H-score: see Methods) from tumors of C57BL/6J mice treated with vehicle, dTAG-13m, or BI-2493. **G**, Phagocytic index in the lung upon dTAG-13m or BI-2493 treatment. All the analysis were performed 6 days after treatment initiation, following daily (dTAG-13m; 40mg/kg) or twice daily (BI-2493; 60 mg/kg) dosing schedules. Statistical differences were analyzed using non-parametric Mann-Whitney test in A, C and E, and non-parametric One-way ANOVA followed by False Discovery Rate multiple comparison tests in the rest of the panels. *, $0.05<p<0.01$; **, $0.01<p<0.001$; ***, $0.001<p<0.0001$; ****, $p<0.0001$. Data are indicated as the mean ± SD. In panels C-G 6 animals per condition were examined.
