# Supplementary material for: Targeted KRASG12V Degradation in vivo Elicits Lung Adenocarcinoma Regression with Subsequent Relapse from Dysregulated Proteolysis
Source: Cancer Res. Author manuscript; Available in PMC 2026 Jun 13. (PMC7619155; doi:10.1158/0008-5472.CAN-25-5172)
Supplement: 7 [file EMS214174-supplement-7.pdf]

Supplementary Table 1. Antibodies and reagents employed in flow cytometry and cell sorting assays

Antibody/Reagent	Clone	Source	Identifier	Panel
CD3 BUV496	17A2	BD Biosciences	Cat#569671,RRID:AB_3668952	1,2
CD3 PE Fire700	17A2	BioLegend	Cat#100272, RRID:AB_2876394	7
CD4 BV605	GK1.5	BD Biosciences	Cat#743156, RRID:AB_2741309	2
CD8a BUV395	53-6.7	BD Biosciences	Cat#563786, RRID:AB_2732919	1,2
CD11b BV421	M1/70	BioLegend	Cat#101236, RRID:AB_11203704	1,2,7
CD11c BUV615	N418	BD Biosciences	Cat#751222, RRID:AB_2875243	1,2,7
CD19 RB545	1D3	BD Biosciences	Cat#569727, RRID: AB_3668953	1,2,7
CD25 BV480	PC61	BD Biosciences	Cat#566120, RRID:AB_2739522	2
CD27 BB700	LG.3A10	BD Biosciences	Cat#742135, RRID:AB_2871394	2
CD43 BV510	S7	BD Biosciences	Cat#563206, RRID:AB_2738069	1,2,7
CD44 APC Vio770	REA664	Miltenyi Biotec	Cat#130-118-695, RRID:AB_2733120	2
CD45 PerCP Cy5.5	I3/2.3	BioLegend	Cat#147705, RRID:AB_2563537	1
CD45 APC	I3/2.3	BioLegend	Cat#147708, RRID:AB_2563540	3,4,5
CD45.2 PerCP	104	BioLegend	Cat#109826, RRID:AB_893349	2,7
CD45R (B220) VioBlue	RA3-6B2	Miltenyi Biotec	Cat#130-118-321, RRID:AB_2733945	1,2
CD62L BUV805	MEL-14	BD Biosciences	Cat#569201, RRID:AB_3668954	2
CD64 PE	X54-5/7.1	BioLegend	Cat#139303, RRID:AB_10613467	1,2,5,7
CD103 PE Fire640	QA17A24	BioLegend	Cat#156917, RRID:AB_2924487	1,2,7
CD127 RB780	A7R34	BD Biosciences	Cat#569066, RRID:AB_3668957	2
CD304 BUV563	V46-1954	BD Biosciences	Cat#752460, RRID:AB_2917455	2
CD335 (NKp46) BV711	29A1.4	BioLegend	Cat#137621, RRID:AB_2563289	1,2,7
F4/80 Spark NIR685	BM8	BioLegend	Cat#123168, RRID:AB_2924461	1,2,5,7
I-A/I-E (MHC-II) BV650	M5/114.15.2	BD Biosciences	Cat#563415, RRID:AB_2738192	1,2,7
Ly6C PE Fire810	HK1.4	BioLegend	Cat#128061, RRID:AB_3083119	1,2,7
Ly6G R718	1A8	BD Biosciences	Cat#567039, RRID:AB_2916402	1,2,7
NK1.1 BV570	PK136	BioLegend	Cat#108733, RRID:AB_10896952	2
Siglec F AF647	E50-2440	BD Biosciences	Cat#562680, RRID:AB_2687570	1,2,7
TCRβ RB545	H57-597	BD Biosciences	Cat#756204, RRID:AB_3668961	2
TruStain FcX PLUS	S17011E	BioLegend	Cat#156604, RRID:AB_2783138	1,2,3,5,7
Brilliant Stain Buffer Plus	N/A	BD Biosciences	Cat#566385	1,2,5,7
True-Stain Monocyte Blocker	N/A	BioLegend	Cat#426102	1,2,5,7
BD FACS™ Lysing Solution 10X	N/A	BD Biosciences	Cat#347691	1,2,3,7
Zombie NIR™ Fixable Viability Kit	N/A	BioLegend	Cat#423105	1,2,3,5,7
Annexin V PE	N/A	Immunostep S.L.	Cat#ANXVPE	6
7-AAD	N/A	Immunostep S.L.	Cat#7AAD	6
Annexin V Binding Buffer 10X	N/A	Immunostep S.L.	Cat#BB10X	6
DAPI	N/A	Sigma-Aldrich	Cat#D9542	4

Panel: 1 Tumor CD45+ cells NSG; 2 Tumor CD45+ cells C57BL6; 3 pre-depleted tumor cell suspension; 4 cell sorting; 5 *In vitro* co-culture; 6 cell death evaluation; 7 clodronate depletion studies;

Abbreviations: AF, Alexa fluor; APC, allophycocyanin; BB, Brilliant Blue; BUV, Brilliant Ultraviolet; BV, Brilliant Violet; Cat#, catalog number; CF, Cyanin-based Fluorescent dye; Cy7, Cyanin7; DAPI, 4',6-diamidino-2-phenylindoledihydrochloride; N/A, not applicable; NIR, near-infrared; PE, Phycoerythrin; PerCP Cy5.5, Peridinin-chlorophyll-protein-cyanin 5.5; RB, RealBlue; RY, RealYellow; R, Red; RRID, Research Resource Identifier
