# Supplementary material for: Targeted KRASG12V Degradation in vivo Elicits Lung Adenocarcinoma Regression with Subsequent Relapse from Dysregulated Proteolysis
Source: Cancer Res. Author manuscript; Available in PMC 2026 Jun 13. (PMC7619155; doi:10.1158/0008-5472.CAN-25-5172)
Supplement: 8 [file EMS214174-supplement-8.pdf]

Supplementary table S2. Immunophenotypic profiles employed for population identification in *ex vivo* tumor-bearing lungs and *in cellulo* co-cultures.

Leukocyte population	Immunophenotype
Eosinophils	SSC^{hi}, Siglec F^{+}, $CD11c^{-/lo}$, $CD3^{-}$, $CD19^{-}$, $CD335^{-}$, $F4/80^{-}$, $Ly6G^{-}$
Neutrophils	SSC^{hi}, $Ly6G^{+}$, $CD11b^{+}$, $CD3^{-}$, $CD11c^{-}$, $CD19^{-}$, $CD335^{-}$, $F4/80^{-}$, Siglec F^{-}
Monocytes	$CD43^{-/+}$, $Ly6C^{-/+}$, $I\text{-}A/I\text{-}E^{-/lo}$, $CD3^{-}$, $CD19^{-}$, $CD335^{-}$, $F4/80^{-}$, $Ly6G^{-}$, Siglec F^{-}
Classical monocytes	$CD43^{-}$, $Ly6C^{+}$
Intermediate monocytes	$CD43^{+}$, $Ly6C^{+}$
Non-classical monocytes	$CD43^{+}$, $Ly6C^{-}$
Macrophages [ex vivo]	SSC^{hi}, $CD11b^{-/+}$, $CD11c^{-/+}$ $CD64^{+}$, $F4/80^{lo/+}$, $I\text{-}A/I\text{-}E^{+}$, Siglec $F^{-/+}$, $CD3^{-}$, $CD19^{-}$, $CD335^{-}$, $Ly6G^{-}$
Alveolar macrophages	$CD11b^{-/+}$ $CD11c^{+}$ $CD64^{+}$, $F4/80^{lo}$, $I\text{-}A/I\text{-}E^{+}$, Siglec F^{+}
Interstitial macrophages	$CD11b^{+}$ $CD11c^{-/+}$ $CD64^{+}$, $F4/80^{+}$, $I\text{-}A/I\text{-}E^{+}$, Siglec F^{-}
Macrophages [in vitro co-cultures]	SSC^{hi}, $CD45^{+}$, $CD64^{+}$, $F4/80^{+}$
Myeloid dendritic cells (DC)	$B220^{-}$, $CD11b^{-/+}$ $CD11c^{+}$, $CD8a^{-/+}$, $CD103^{-/+}$, $I\text{-}A/I\text{-}E^{++}$, $CD3^{-}$, $CD19^{-}$, $CD335^{-}$, $F4/80^{-}$, $Ly6G^{-}$, Siglec F^{-}
Conventional type 2 DC (cDC2)	$CD11b^{+}$, $CD8a^{-}$, $CD103^{-}$
$CD103^{+}$ DC	$CD11b^{-/lo}$, $CD8a^{-/+}$, $CD103^{+}$
Total lymphocytes	$SSC^{lo/int}$, $CD45^{hi}$ ($B220^{-/+}$ and/or $CD3^{-/+}$ and/or $CD19^{-/+}$ and/or $CD335^{-/+}$ and/or $NK1.1^{-/+}$)
NK cells	SSC^{lo}, $CD45^{hi}$, CD335+, $NK1.1^{+}$, $B220^{-}$, $CD3^{-}$, $CD19^{-}$
Mature NK cells	$CD11b^{+}$, $CD27^{lo}$
T cells	SSC^{lo}, $CD45^{hi}$, $CD3^{+}$, $TCR\beta^{-/+}$, $CD19^{-}$, $CD335^{-}$, $NK1.1^{-}$
$CD4^{+}$ $CD8^{-}$ T cells	$CD4^{+}$, $CD8^{-}$, $TCR\beta^{+}$
Regulatory T cells (Treg)	$CD4^{+}$, $CD8^{-}$, $CD127^{lo}$, ($CD25^{+}$ and/or $CD304^{+}$), $TCR\beta^{+}$
$CD25^{+}$ Treg	$CD4^{+}$, $CD8^{-}$, $CD25^{+}$, $CD127^{lo}$, $CD304^{-/+}$, $TCR\beta^{+}$
$CD25^{+}$ Naïve/Central Treg	$CD44^{-/lo}$, $CD62L^{+}$
$CD25^{+}$ Effector Treg	$CD44^{+}$, $CD62L^{-/lo}$
$CD25^{-}$ Treg	$CD4^{+}$, $CD8^{-}$, $CD25^{-}$, $CD127^{lo}$, $CD304^{+}$, $TCR\beta^{+}$
$CD25^{-}$ Naïve/Central Treg	$CD44^{-/lo}$, $CD62L^{+}$
$CD25^{-}$ Effector Treg	$CD44^{+}$, $CD62L^{-/lo}$
T helper (Th) $CD4^{+}$ T cells	$CD4^{+}$, $CD8^{-}$, $TCR\beta^{+}$, NOT Treg
$CD4^{-}$ $CD8^{+}$ T cells	$CD4^{-}$, $CD8^{+}$, $TCR\beta^{+}$
$CD4^{+}$ $CD8^{+}$ T cells	$CD4^{+}$, $CD8^{+}$, $TCR\beta^{+}$
$CD4^{-}$ $CD8^{-/lo}$ $TCR\alpha\beta$+ T cells	$CD4^{-}$, $CD8^{-/lo}$, $TCR\beta^{+}$
$CD4^{-}$ $CD8^{-/lo}$ $TCR\gamma\delta$+ T cells	$CD4^{-}$, $CD8^{-/lo}$, $TCR\beta^{-}$
B cells	$SSC^{lo/int}$, $CD45^{hi}$, $CD19^{+}$, $CD3^{-}$, $CD335^{-}$, $NK1.1^{-}$

Abbreviations: *hi, high; int, intermediate; lo, low; SSC, side scatter.*
