# Supplementary material for: Targeted KRASG12V Degradation in vivo Elicits Lung Adenocarcinoma Regression with Subsequent Relapse from Dysregulated Proteolysis
Source: Cancer Res. Author manuscript; Available in PMC 2026 Jun 13. (PMC7619155; doi:10.1158/0008-5472.CAN-25-5172)
Supplement: 9 [file EMS214174-supplement-9.pdf]

Supplementary Table S3. PRM

PG.ProteinAccessions	FG.Precursor	m/z	z	t start (min)	t stop (min)	HCD Collision Energy (%)
Biognosys st	LGGNEQVT	487,2567	2	4,9	10,9	28
Biognosys st	GAGSSEPV	644,8226	2	16,8	22,8	28
Biognosys st	VEATFGVD	683,8279	2	23,5	29,5	28
Biognosys st	YILAGVENS	547,298	2	28,5	34,5	28
Biognosys st	TPVISGGP	669,8381	2	31,6	37,6	28
Biognosys st	TPVITGAP	683,8537	2	34,9	40,9	28
Biognosys st	DGLDAASY	699,3384	2	44,8	50,8	28
Biognosys st	ADVTPADF	726,8357	2	50,5	56,5	28
Biognosys st	GTFIIDPGG	622,8535	2	62,1	68,1	28
Biognosys st	GTFIIDPAA	636,8692	2	69,7	75,7	28
Biognosys st	LFLQFGAQ	776,9298	2	73,8	79,8	28
sp|A2A432|	LITYLDQTT	662,3614	2	33,5	39,5	28
sp|A2A432|	GLNSLLDE	565,7936	2	39,7	45,7	28
sp|A2A432|	AFGSTIVIN	638,3508	2	41	47	28
sp|A2A432|	TLSHNLLVS	620,0071	3	59,2	65,2	28
sp|O35864|	ISALALLK	414,7813	2	54,9	60,9	28
sp|O35864|	LLELLWNK	514,8106	2	67,3	73,3	28
sp|O35864|	LEQSEAQL	565,7936	2	11	17	28
sp|O88543|	SGELLAK	359,2107	2	10,9	16,9	28
sp|O88543|	YTSQIVGR	462,2509	2	11,5	17,5	28
sp|O88543|	VQLSGPQE	593,3091	2	12,7	18,7	28
sp|O88544|	AIQLSGTEC	750,9171	2	50	56	28
sp|O88544|	NAAQVLVG	819,4647	2	60	66	28
sp|O88544|	FIEAAQR	417,7271	2	10,1	16,1	28
sp|O88544|	ATTADGSS	603,8017	2	21,4	27,4	28
sp|O88545|	LILEYVK	439,2733	2	43,7	49,7	28
sp|O88545|	ASEAGEVP	556,9495	3	36,1	42,1	28
sp|O88545|	FNVLYDR	463,7402	2	35,5	41,5	28
sp|P61082|	DINELNLPK	528,2902	2	41,2	47,2	28
sp|P61082|	VGQGYPH	597,7988	2	5,4	11,4	28
sp|P61082|	LFEQNVQR	517,2749	2	16,4	22,4	28
sp|P61082|	GGYIGSTY	625,296	2	38,9	44,9	28
sp|P61202|	SINSILDYIS	720,8827	2	71	77	28
sp|P61202|	ALYEQSLHI	601,3324	2	25,3	31,3	28
sp|P61202|	IDQVNQLL	564,9687	3	47,9	53,9	28
sp|P61202|	WTNQLNS	851,4496	2	44,4	50,4	28
sp|P62254|	NPVEGFSA	997,9763	2	65,6	71,6	28
sp|P62254|	WEVLIIGPP	1067,067	2	83,2	88	28
sp|P62254|	FITEIWHP	500,2611	3	48,2	54,2	28

sp|P62878|F	QVCPLDNR	501,2453	2	11,7	17,7	28
sp|P62878|F	EWEFQK	433,7058	2	27,3	33,3	28
sp|Q02053|	YDGQVAV	845,3914	2	49,8	55,8	28
sp|Q02053|	LAGTQPLE	812,4568	2	65,6	71,6	28
sp|Q02053|	AENYDISP	625,786	2	18	24	28
sp|Q3TCH7|	LPDNYTQD	704,8282	2	32,1	38,1	28
sp|Q3TCH7|	AIQSSTSIR	481,7669	2	7,8	13,8	28
sp|Q3TCH7|	FIFNADFK	501,2582	2	57,6	63,6	28
sp|Q3TCH7|	TLGHNLLV	614,6755	3	65,1	71,1	28
sp|Q3U1J4|	YLAIAPPIIK	549,8497	2	59,1	65,1	28
sp|Q3U1J4|	LVFSNVNL	517,3057	2	43,8	49,8	28
sp|Q3U1J4|	TVPLYESPR	531,2849	2	23,2	29,2	28
sp|Q3UE37|	TAPQCLLR	479,7606	2	22,4	28,4	28
sp|Q3UE37|	FNPNFYR	479,2325	2	29,8	35,8	28
sp|Q6ZQ38|	TVSPALIAR	464,2847	2	26,2	32,2	28
sp|Q6ZQ38|	ALTLIAGSP	542,3422	2	52,3	58,3	28
sp|Q6ZQ38|	AVAALLTIP	663,3874	2	56,9	62,9	28
sp|Q8BV13|	ESLPELSVA	721,8779	2	36,3	42,3	28
sp|Q8BV13|	TQQQVEA	737,3808	2	24,7	30,7	28
sp|Q8C7D2|	DDSLPENP	834,381	2	61,9	67,9	30
sp|Q8C7D2|	SALLPTIPE	977,9964	2	60,9	66,9	30
sp|Q8C7R4|	LETGQFLT	606,3246	2	57,3	63,3	28
sp|Q8C7R4|	DGSLFWQ	582,7878	2	52	58	28
sp|Q8C7R4|	FDLNEPLH	644,0071	3	70,6	76,6	28
sp|Q8C7R4|	NAVFQLEE	775,3965	2	64,5	70,5	28
sp|Q8C878|	NLALSGFR	439,2481	2	38,3	44,3	28
sp|Q8C878|	AEVAAEFL	617,8068	2	41,4	47,4	28
sp|Q8C878|	SPAITATLE	544,3033	2	28,9	34,9	28
sp|Q8VBV7|	SANSELGG	780,8919	2	53,1	59,1	28
sp|Q8VBV7|	GVLEQGW	774,3761	2	32,6	38,6	28
sp|Q8VBW6	DAAAVGN	526,7778	2	3,1	9,1	28
sp|Q8VBW6	LLQSVGQA	793,4252	2	30,7	36,7	28
sp|Q8VBW6	SLAEEYGLD	719,8566	2	39,1	45,1	28
sp|Q8VBW6	YGAAEPHT	762,4023	3	66,3	72,3	28
sp|Q99LD4|	LQFIADR	431,7427	2	30,9	36,9	28
sp|Q99LD4|	AESTPEIAE	615,8017	2	12,5	18,5	28
sp|Q99LD4|	LFLELEPQV	622,3559	2	62,1	68,1	28
sp|Q9D9Z5|	GLPVYNK	395,7265	2	16,4	22,4	28
sp|Q9D9Z5|	FHADSVCK	482,2213	2	3,9	9,9	28
sp|Q9D9Z5|	EYPSEQIIV	718,3694	2	34,4	40,4	28
sp|Q9QZ73|	TAVSCLSQ	704,8299	2	29,8	35,8	28
sp|Q9QZ73|	LDVATDNF	1028,013	2	75,6	81,6	28
sp|Q9QZ73|	FLDLWNK	468,2529	2	59,8	65,8	28
sp|Q9QZ73|	FLLEHHK	462,2585	2	9,3	15,3	28
