# Supplementary material for: Targeted KRASG12V Degradation in vivo Elicits Lung Adenocarcinoma Regression with Subsequent Relapse from Dysregulated Proteolysis
Source: Cancer Res. Author manuscript; Available in PMC 2026 Jun 13. (PMC7619155; doi:10.1158/0008-5472.CAN-25-5172)
Supplement: 10 [file EMS214174-supplement-10.pdf]

Supplementary Table S4

Genomic DNA sequencing by Sanger of dTAG-2xHA-KRAS^{G12V}

Control cell lines

C1

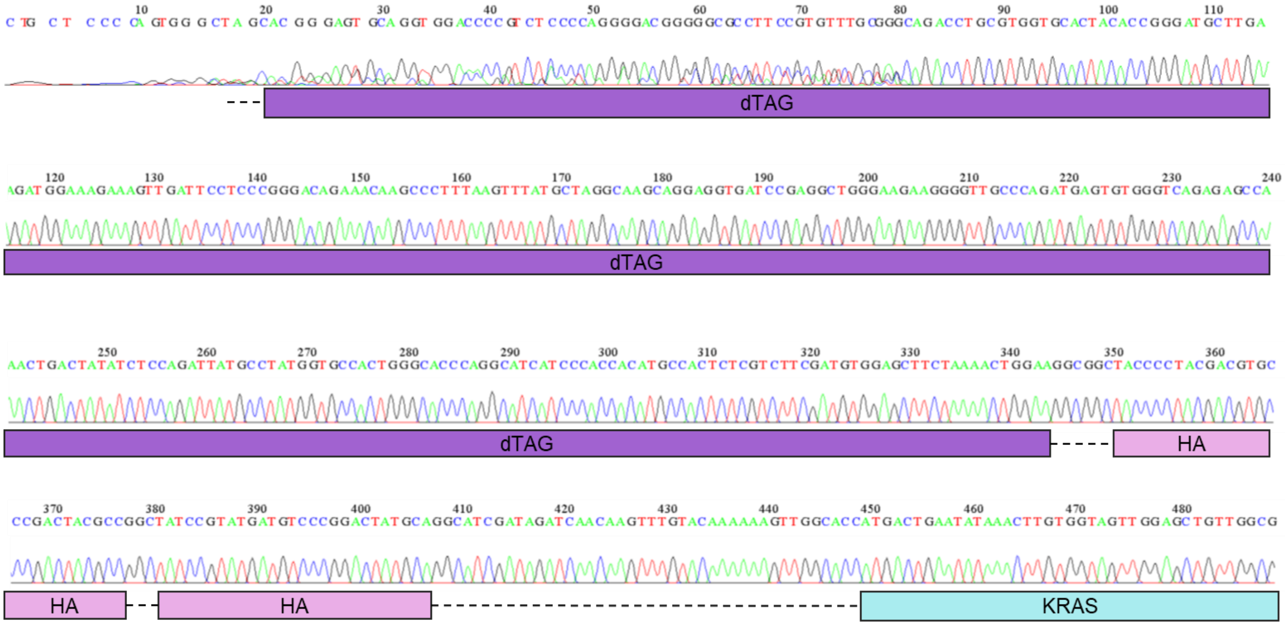

C2

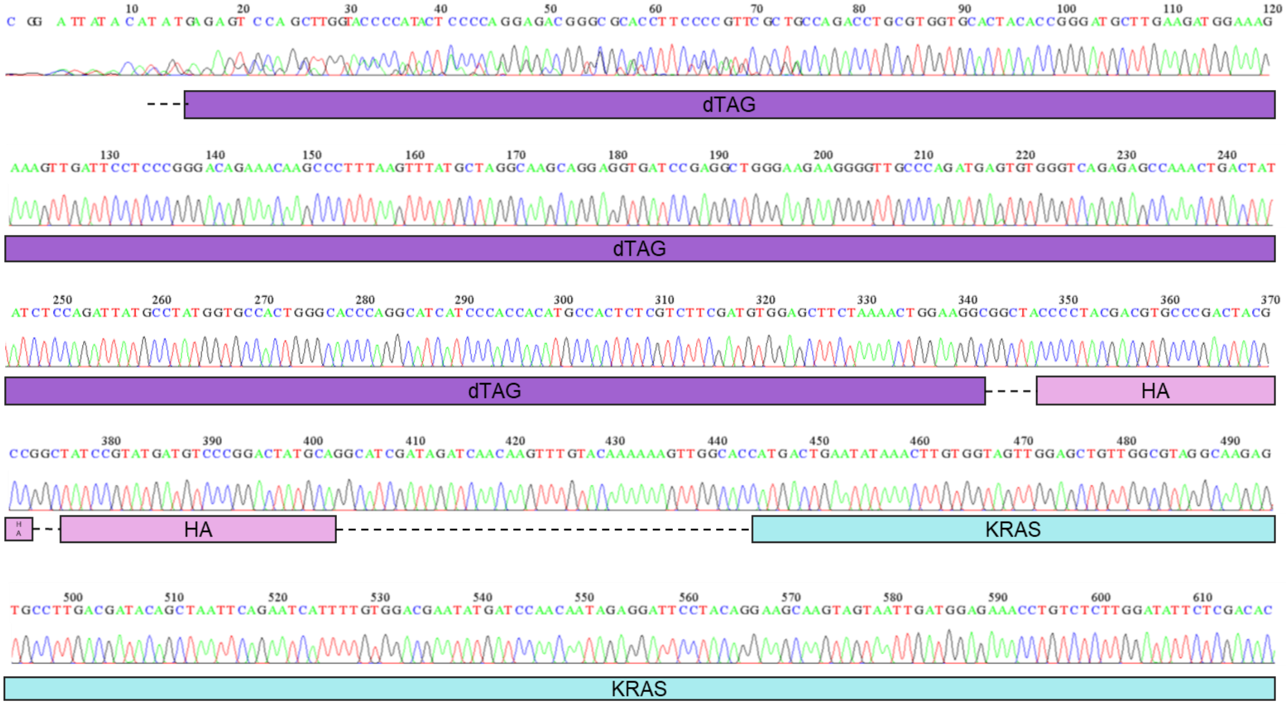

C3

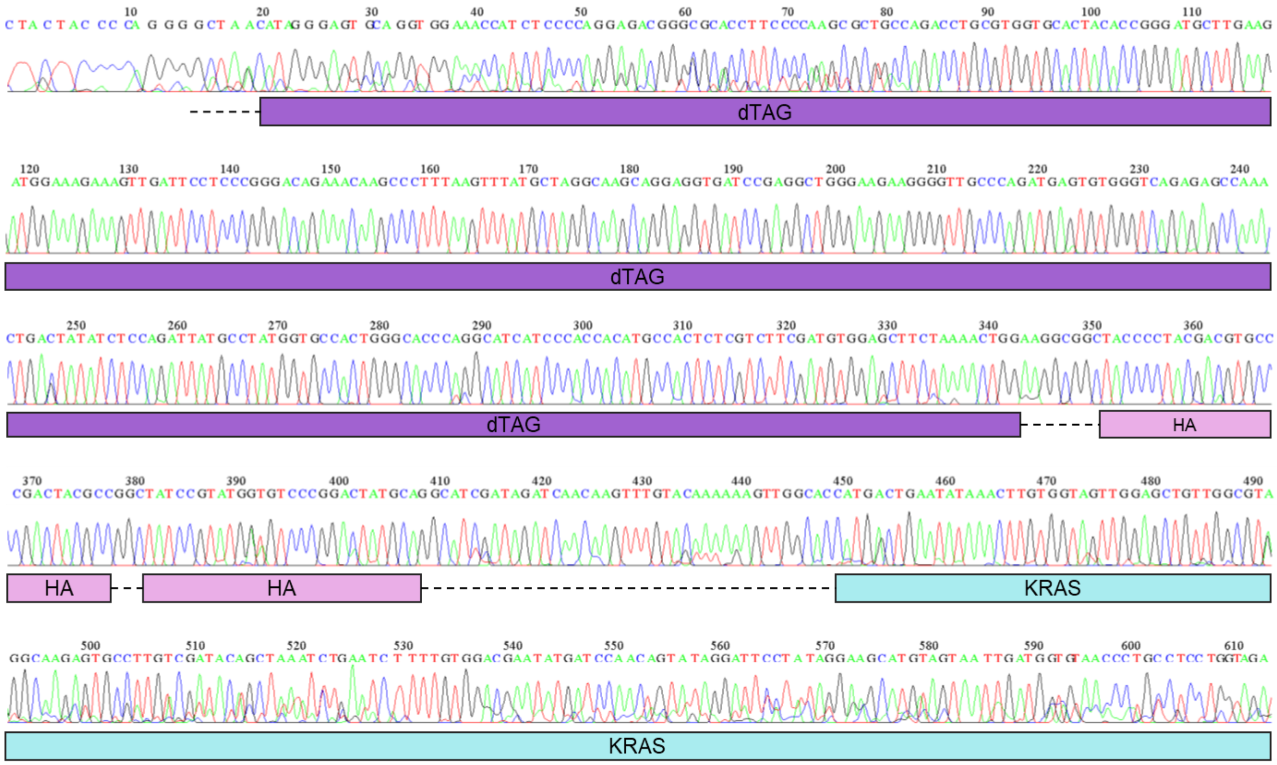
dTAG
dTAG
dTAG
HA
HA
HA
KRAS
KRAS

C4

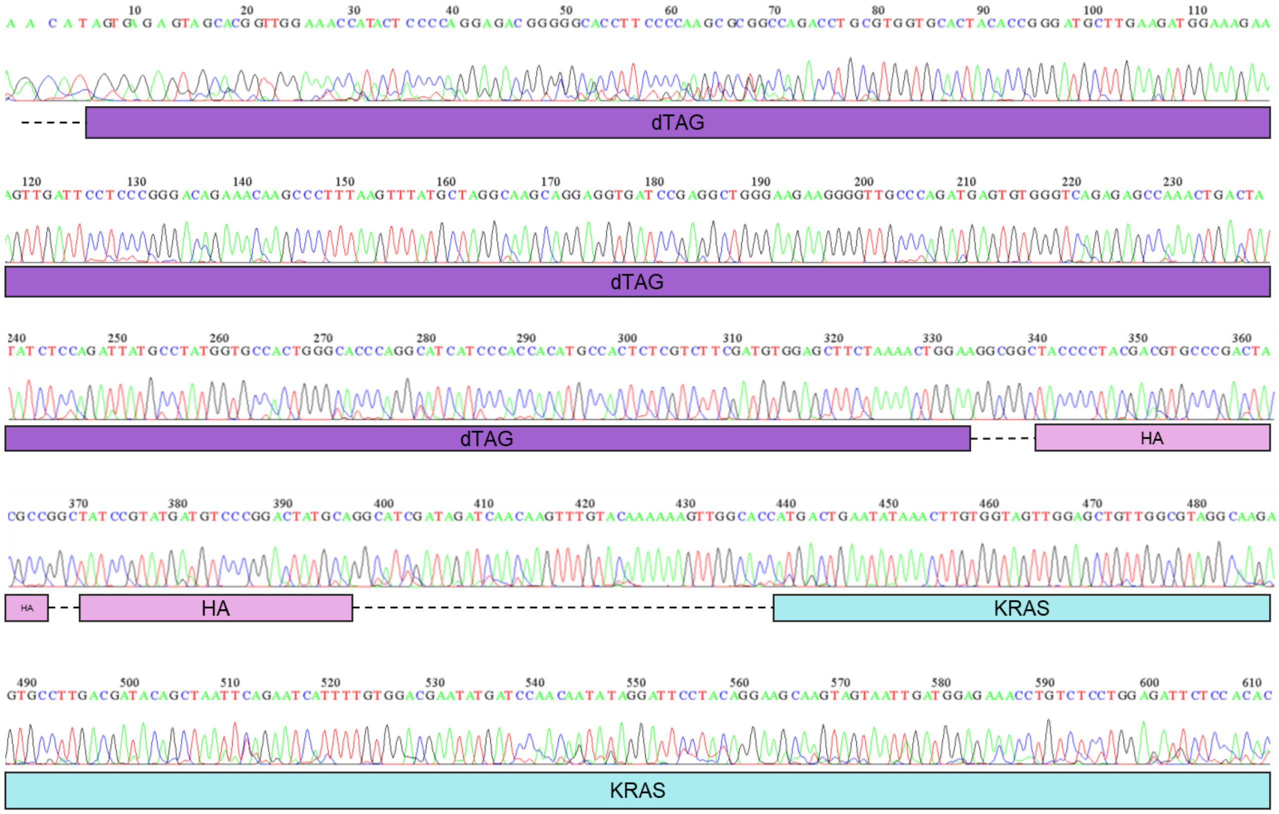
dTAG
dTAG
dTAG
HA
HA
HA
KRAS
KRAS

C5

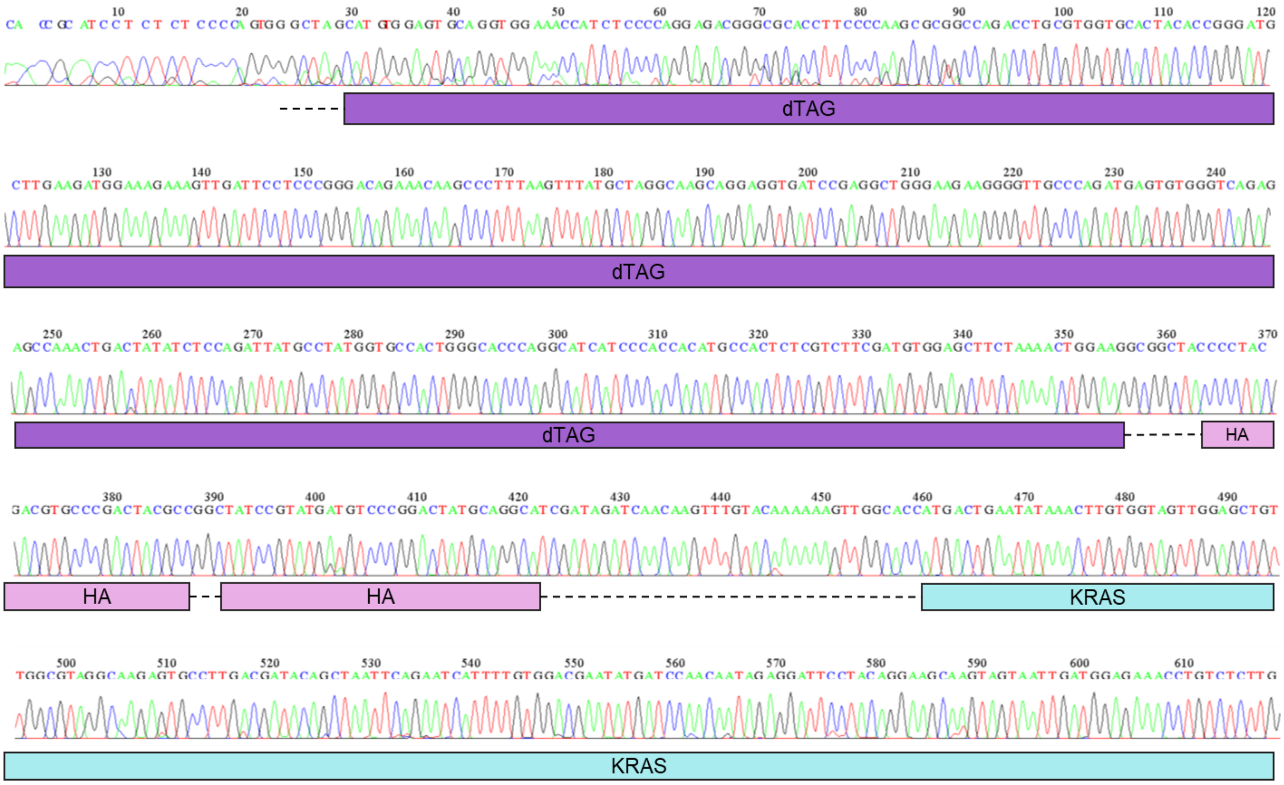
dTAG
dTAG
dTAG
HA
HA
HA
KRAS
KRAS

C6

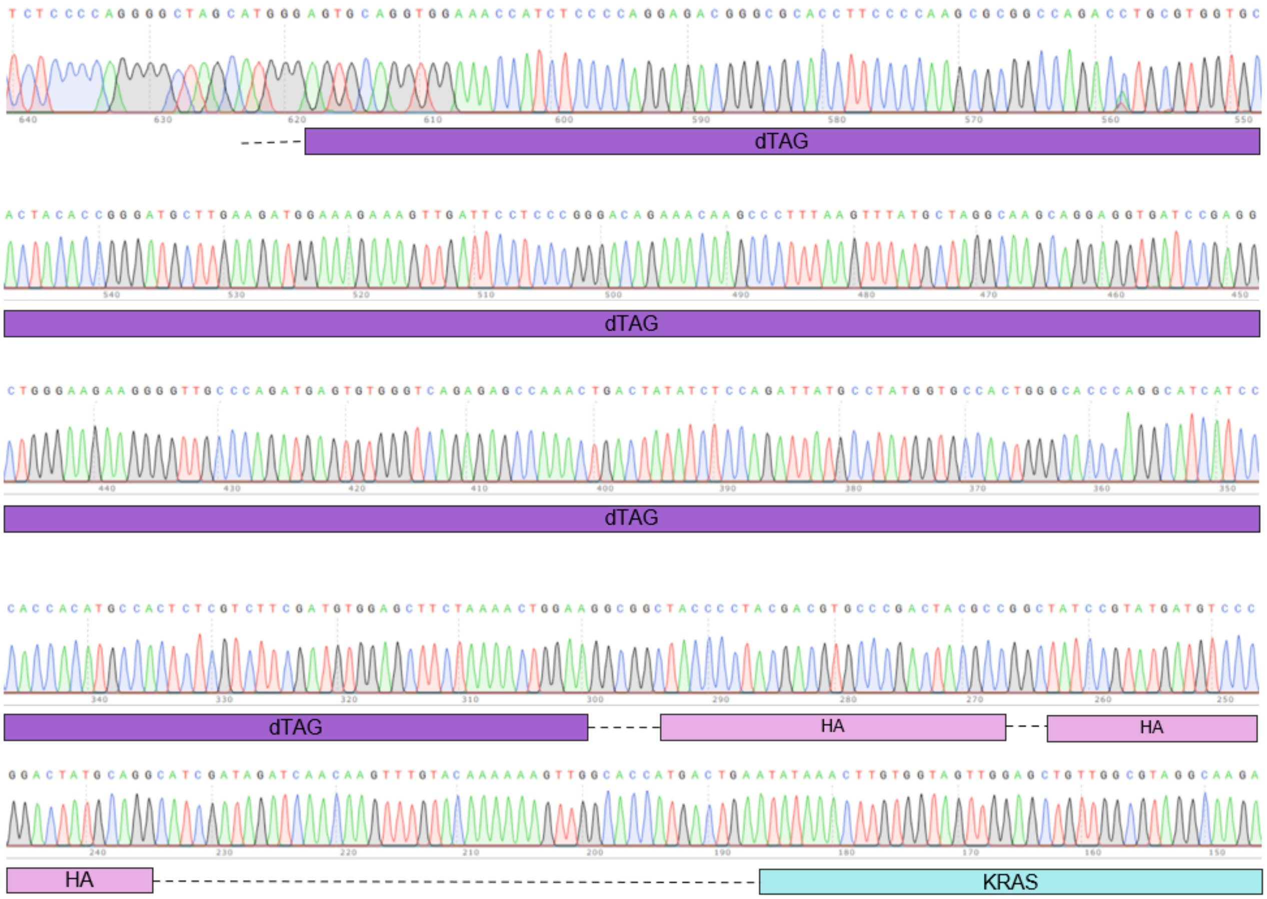
dTAG
dTAG
dTAG
dTAG
HA
HA
HA
KRAS

Resistant cell lines

R1

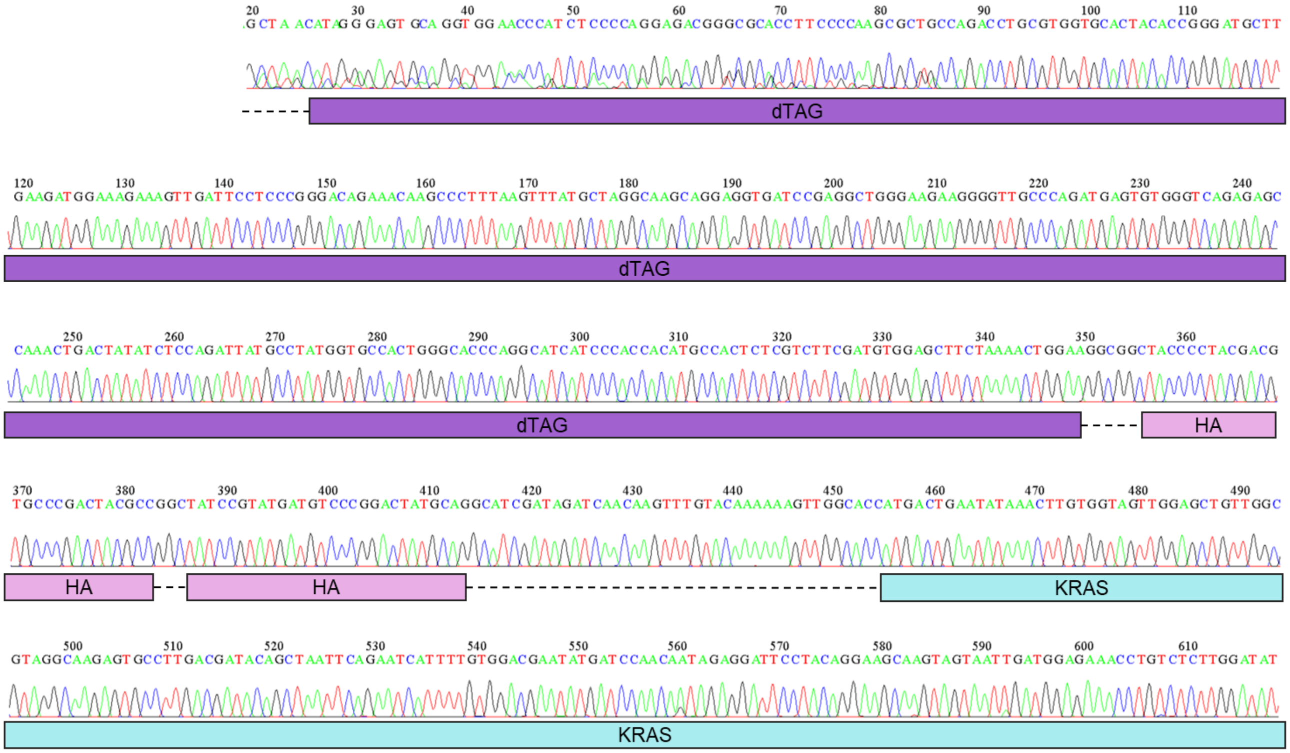

R2

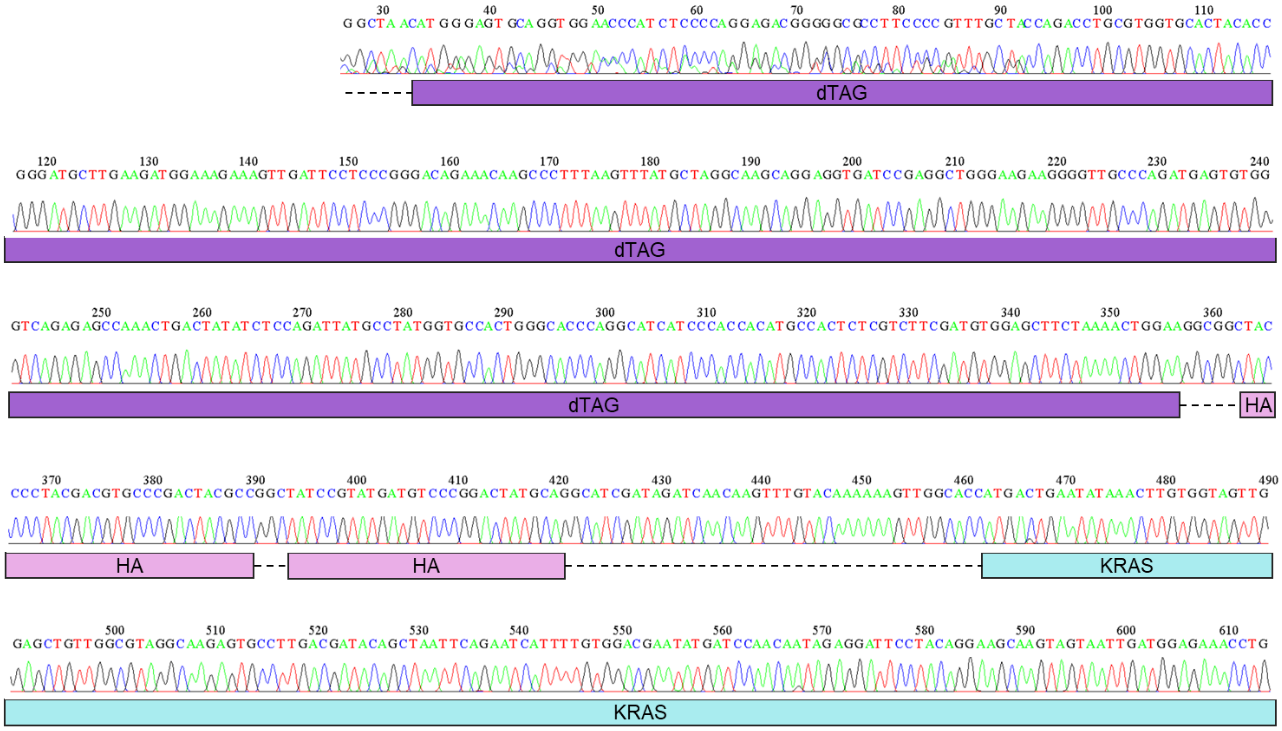

R4

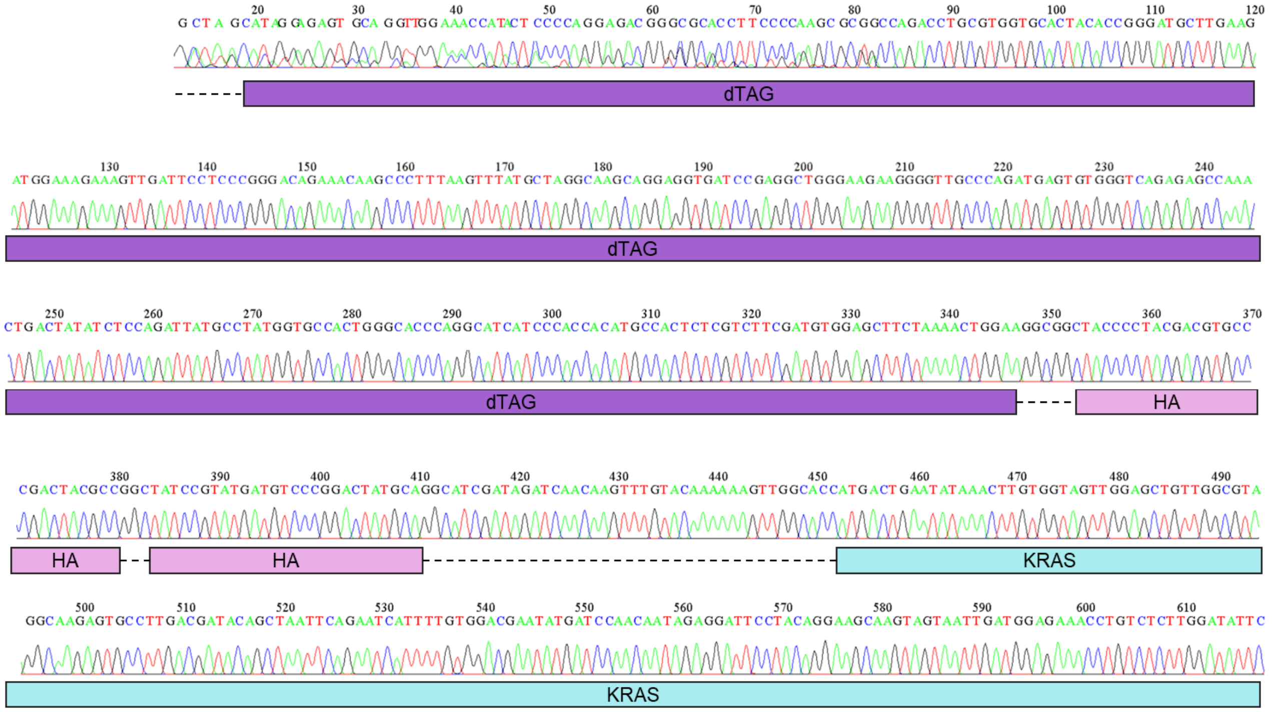
dTAG
dTAG
dTAG
HA
HA
HA
KRAS
KRAS

R5

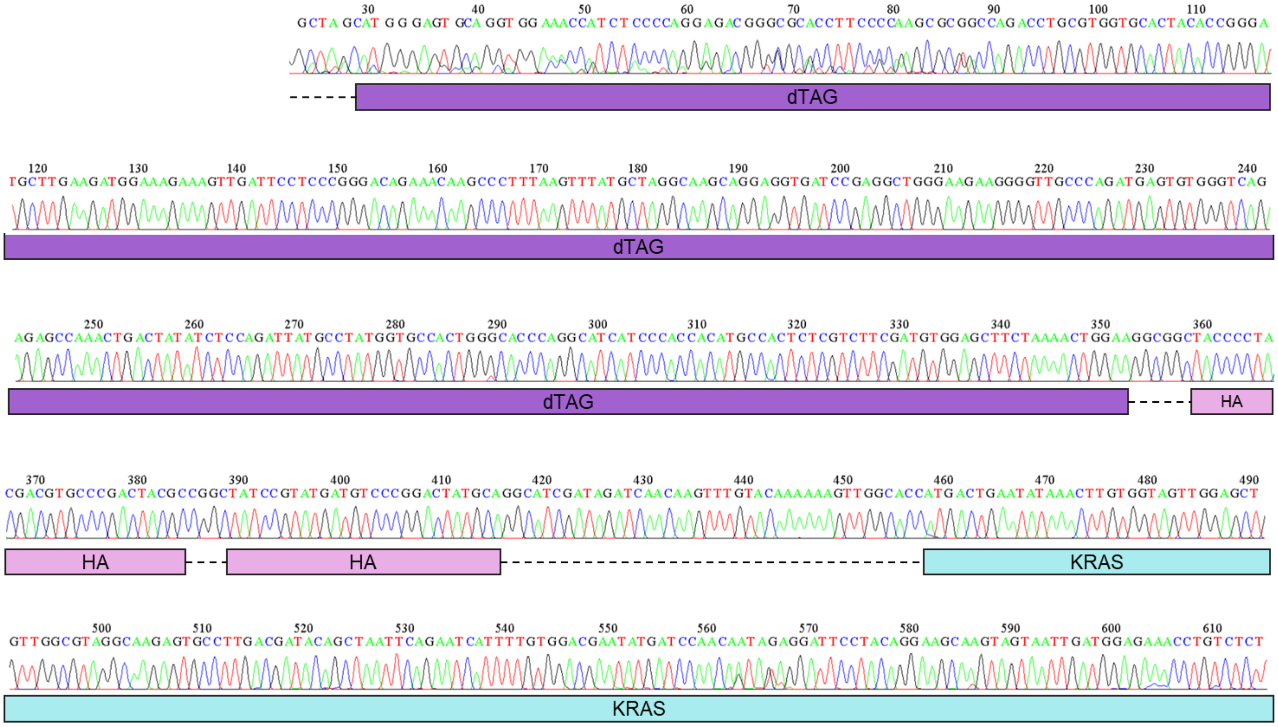
dTAG
dTAG
dTAG
HA
HA
HA
KRAS
KRAS

R6

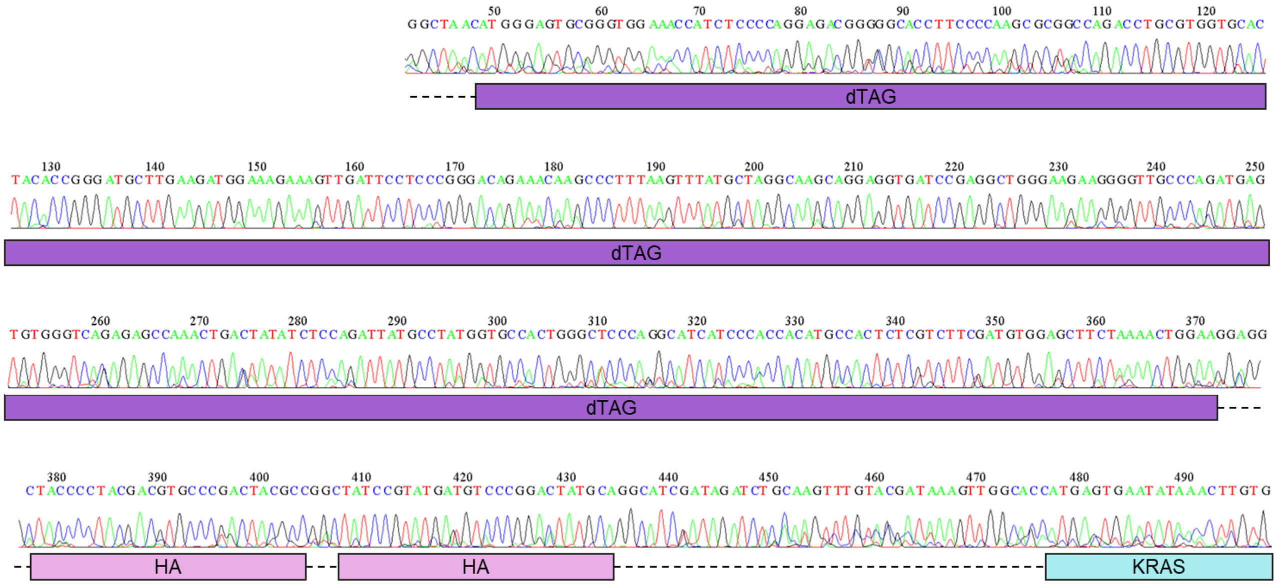

R7

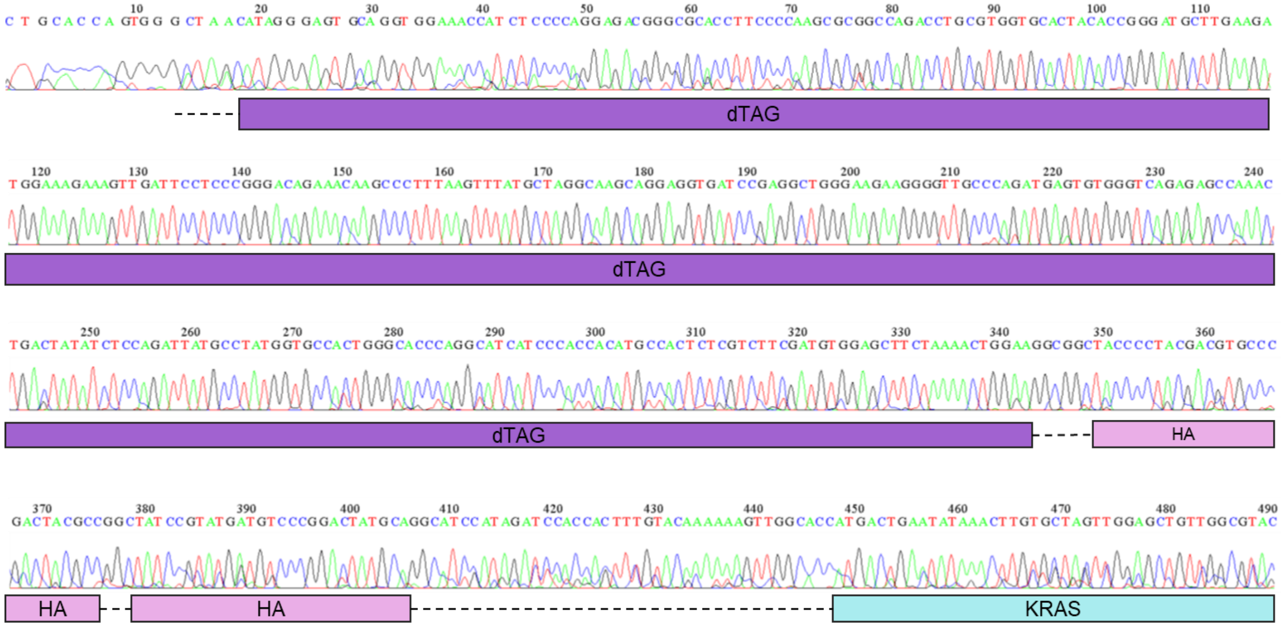

R8

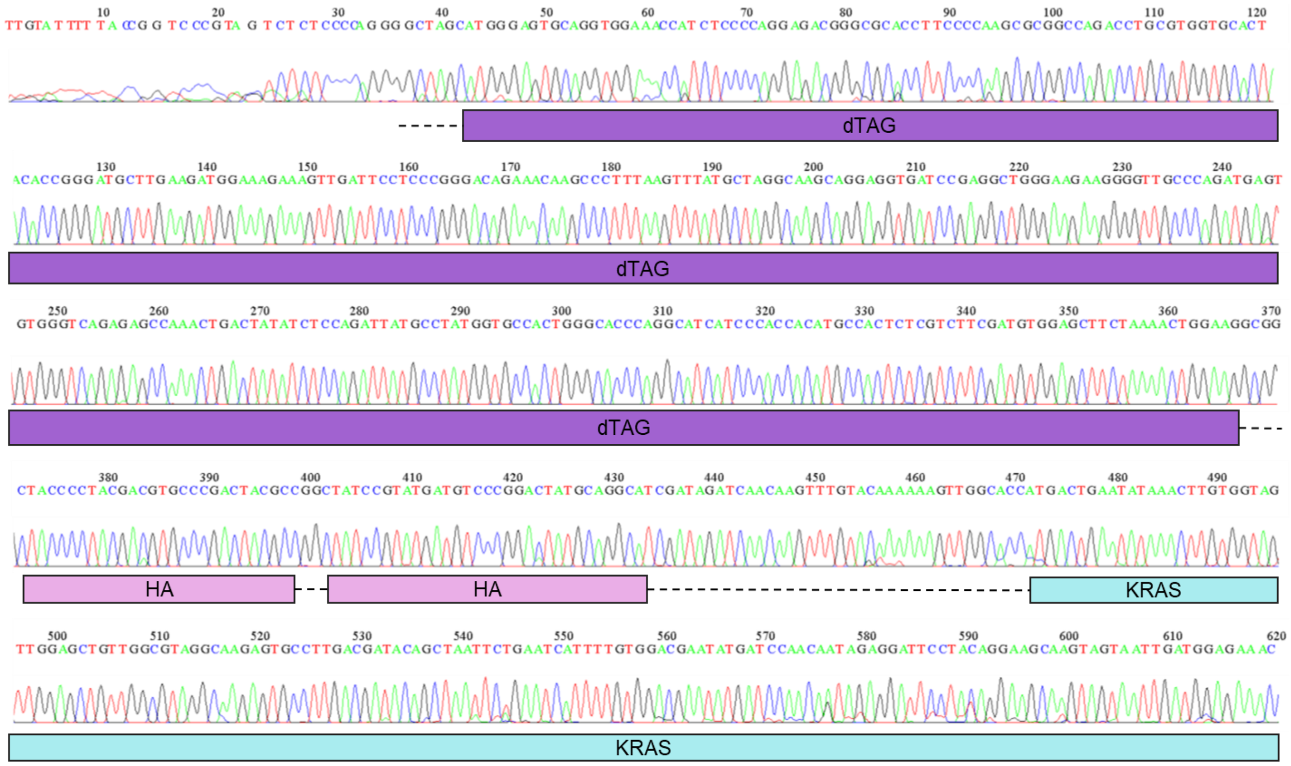
dTAG
dTAG
dTAG
HA
HA
KRAS
KRAS

R9

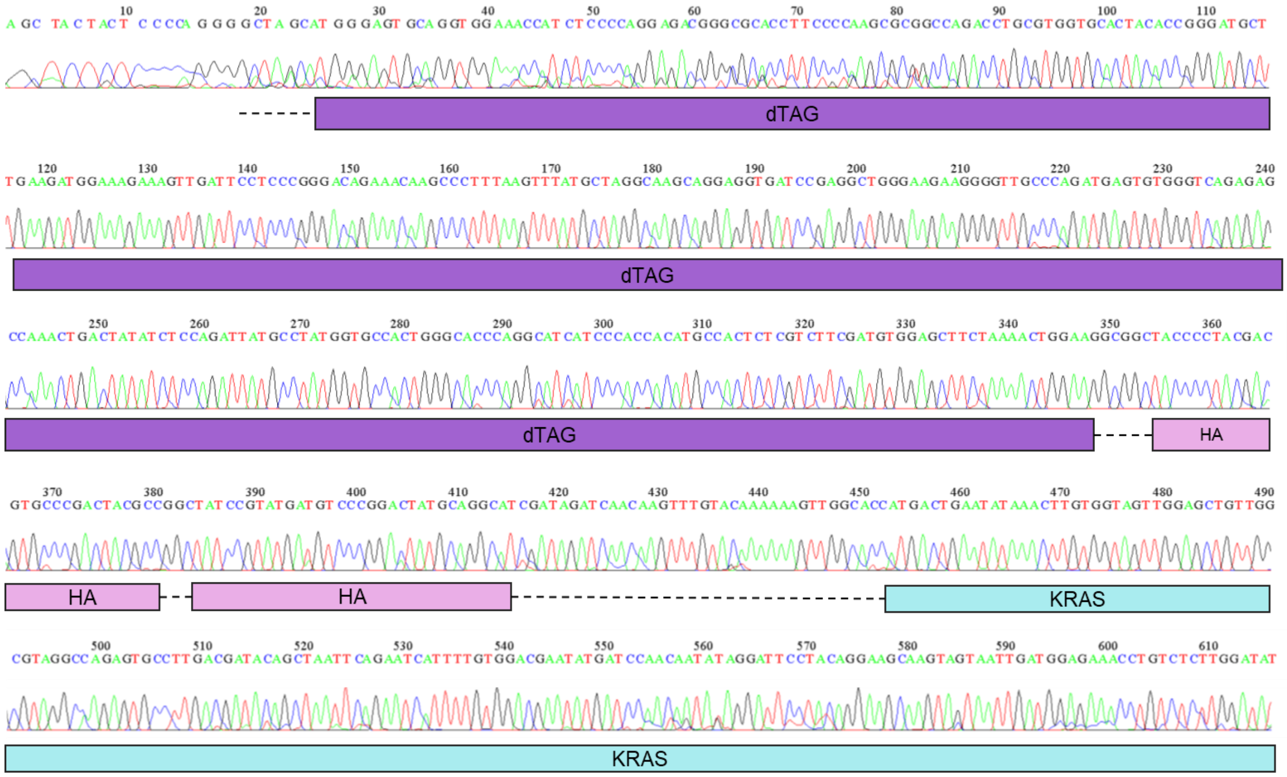
dTAG
dTAG
dTAG
HA
HA
HA
KRAS
KRAS

R10

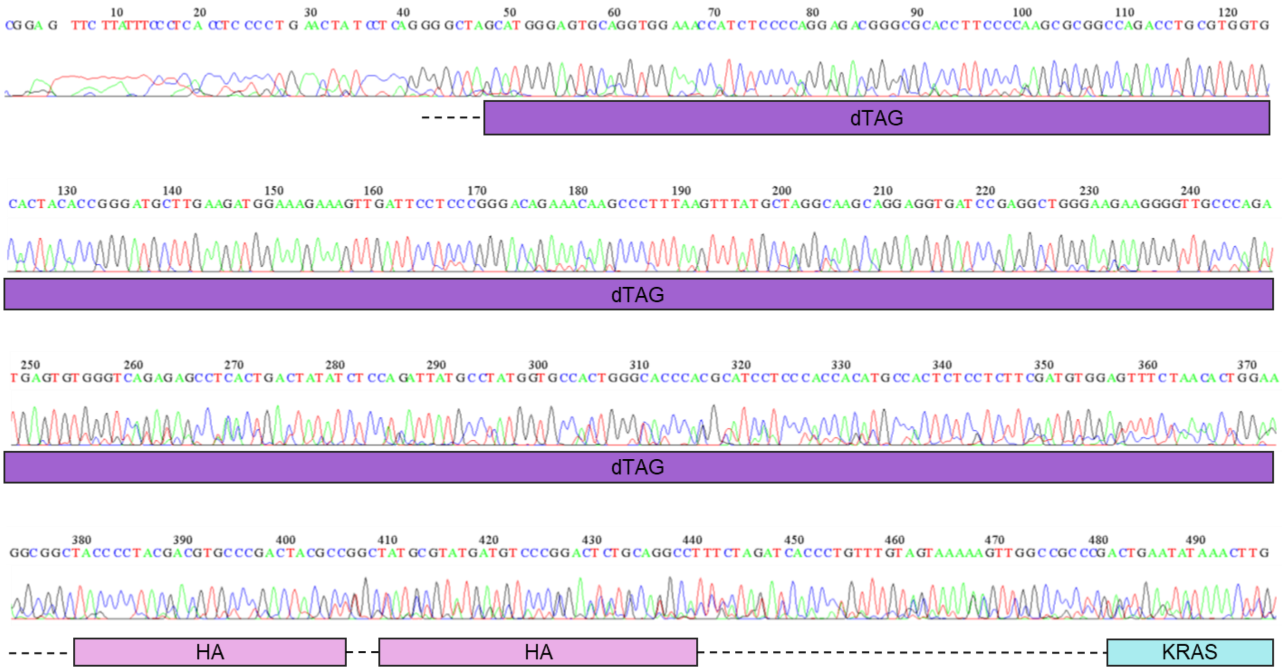

R11

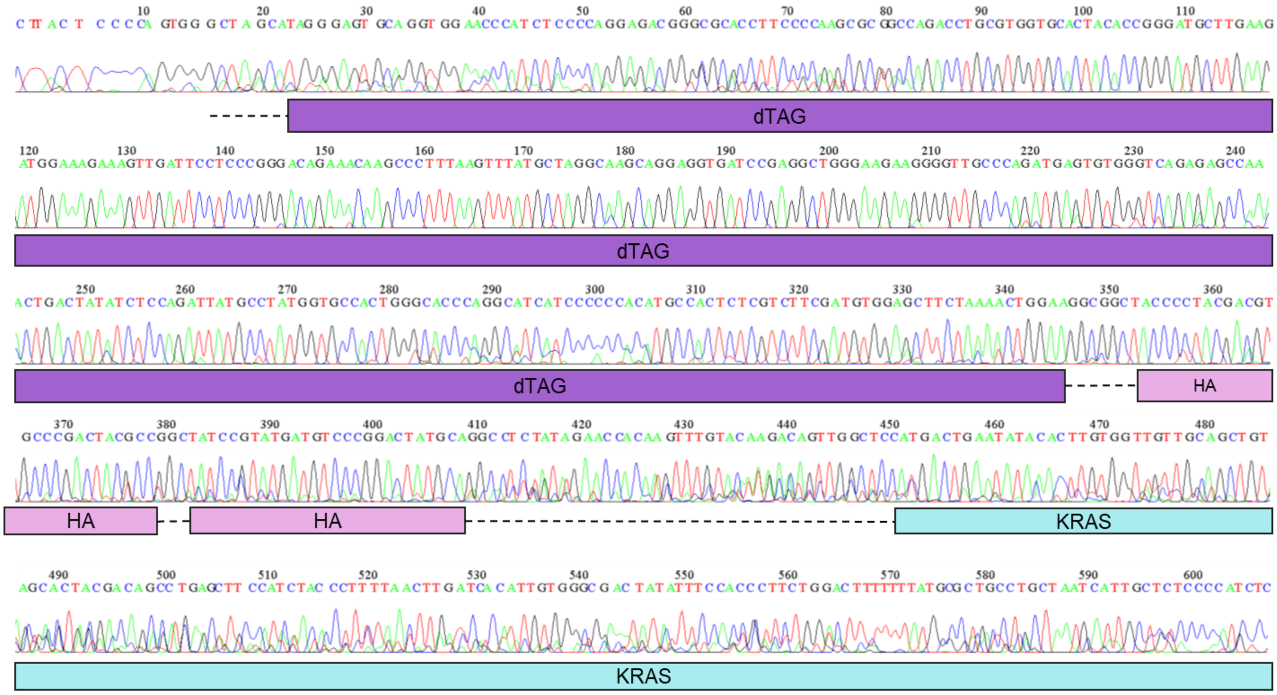

R12

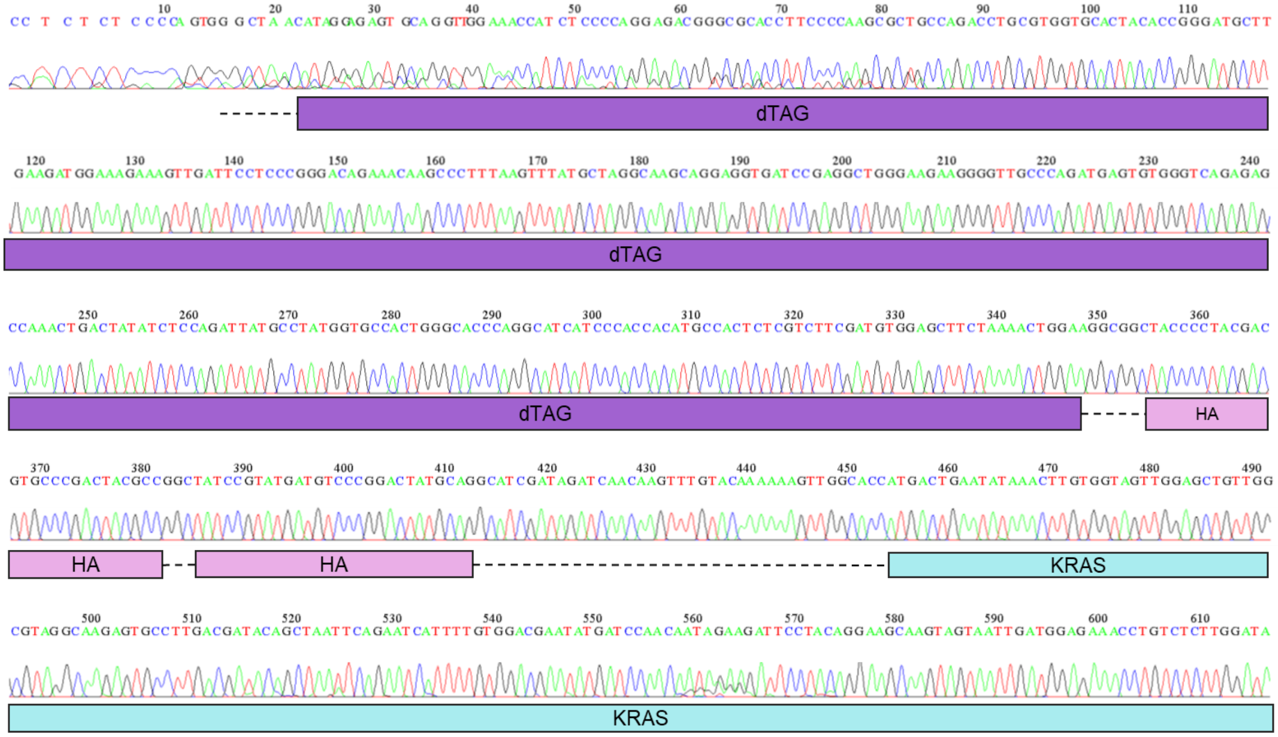
dTAG
dTAG
dTAG
HA
HA
HA
KRAS
KRAS

R13

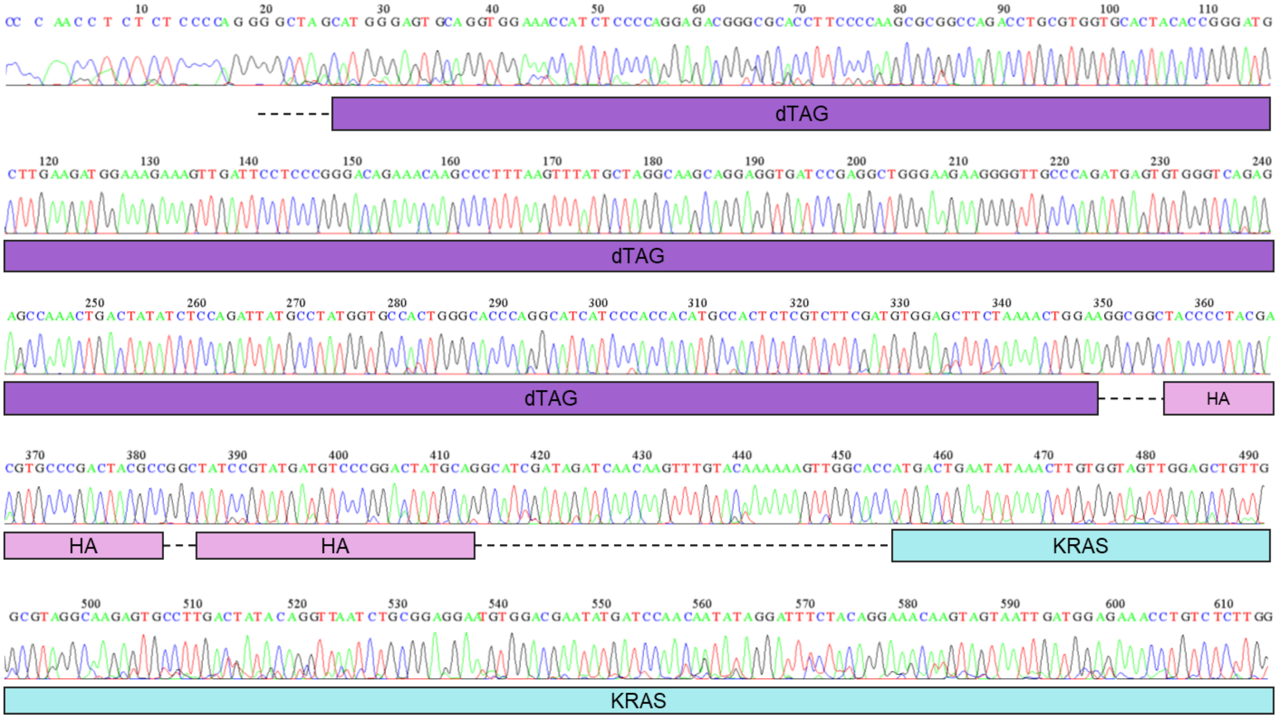
dTAG
dTAG
dTAG
HA
HA
HA
KRAS
KRAS
