# Supplementary material for: Targeted KRASG12V Degradation in vivo Elicits Lung Adenocarcinoma Regression with Subsequent Relapse from Dysregulated Proteolysis
Source: Cancer Res. Author manuscript; Available in PMC 2026 Jun 13. (PMC7619155; doi:10.1158/0008-5472.CAN-25-5172)

Supplementary Material: representative IVIS images

Targeted KRAS^{G12V} Degradation *in vivo* Elicits Lung Adenocarcinoma Regression with Subsequent Relapse from Dysregulated Proteolysis

Alberto Martín[1,#,*], Inés M. García-Pérez[2,#], Sonia San José[1], Pep Rojo[2], Carlos Riego-Mejías[2], Cristina Teodosio[3], Bárbara MG. Barbosa[2], Carolina Sánchez-Zarzalejo[2], Ignasi Folch-I-Casanovas[2], Antonia Odena Caballol[2], Sònia Jarió[2], Sara Hijazo-Pechero[2], Silvia M. Rodríguez-López[1], Rodrigo Entrialgo-Cadierno[4], Marie-Julie Nokin[5], José M. Muñoz-Félix[6], Diana Loa[7], Elizabeth Guruceaga[4], Camille Stephan-Otto Attolini[2], Chiara Ambrogio[8], Alberto Villanueva[9], Silvestre Vicent[4,10,11,12], Antoni Riera[2,13], David Santamaría[1,*] & Cristina Mayor-Ruiz[2,*]

[1] Molecular Mechanisms of Cancer Program, Centro de Investigación del Cáncer (CIC), CSIC-Universidad de Salamanca, Salamanca, Spain
[2] Institute for Research in Biomedicine (IRB Barcelona), the Barcelona Institute of Science and Technology (BIST), Barcelona, Spain
[3] Translational and Clinical Research Program, Cancer Research Center (IBMCC, CSIC – University of Salamanca); Cytometry Service, NUCLEUS; Department of Medicine, University of Salamanca (Universidad de Salamanca), Salamanca, Spain; Institute of Biomedical Research of Salamanca (IBSAL), Salamanca, Spain; Biomedical Research Networking Centre Consortium of Oncology (CIBERONC), Instituto de Salud Carlos III, Madrid, Spain.
[4] University of Navarra, Center for Applied Medical Research, Program in Solid Tumors, Pamplona, Spain
[5] Laboratory of Biology of Tumor and Development (LBTD), GIGA-Cancer, University of Liege, Liege, Belgium
[6] Departamento de Bioquímica y Biología Molecular, Universidad de Salamanca, Instituto de Investigación Biomédica de Salamanca (IBSAL), Salamanca, Spain.
[7] Servicio de Experimentación Animal, Universidad de Salamanca, Salamanca, Spain
[8] Department of Molecular Biotechnology and Health Sciences, Molecular Biotechnology Center, University of Torino, Torino, Italy
[9] Procure Program, Catalan Institute of Oncology (ICO), L'Hospitalet de Llobregat, Barcelona, Spain
[10] Centro de Investigación Biomédica en Red de Cáncer (CIBERONC), Madrid, Spain.
[11] Navarra Health Institute (IDISNA), Pamplona, Spain.
[12] University of Navarra, Department of Pathology, Anatomy and Physiology, Pamplona, Spain.
[13] Departament de Química Inorgànica i Orgànica, Universitat de Barcelona, Barcelona, Spain

# Co-first authors
* Co-corresponding authors: almmartin@usal.es, d.santamaria@usal.es, & cristina.mayor-ruiz@irbbarcelona.org

IVIS images showing tumor regression upon induction of KRAS^{G12V} degradation in NSG mice

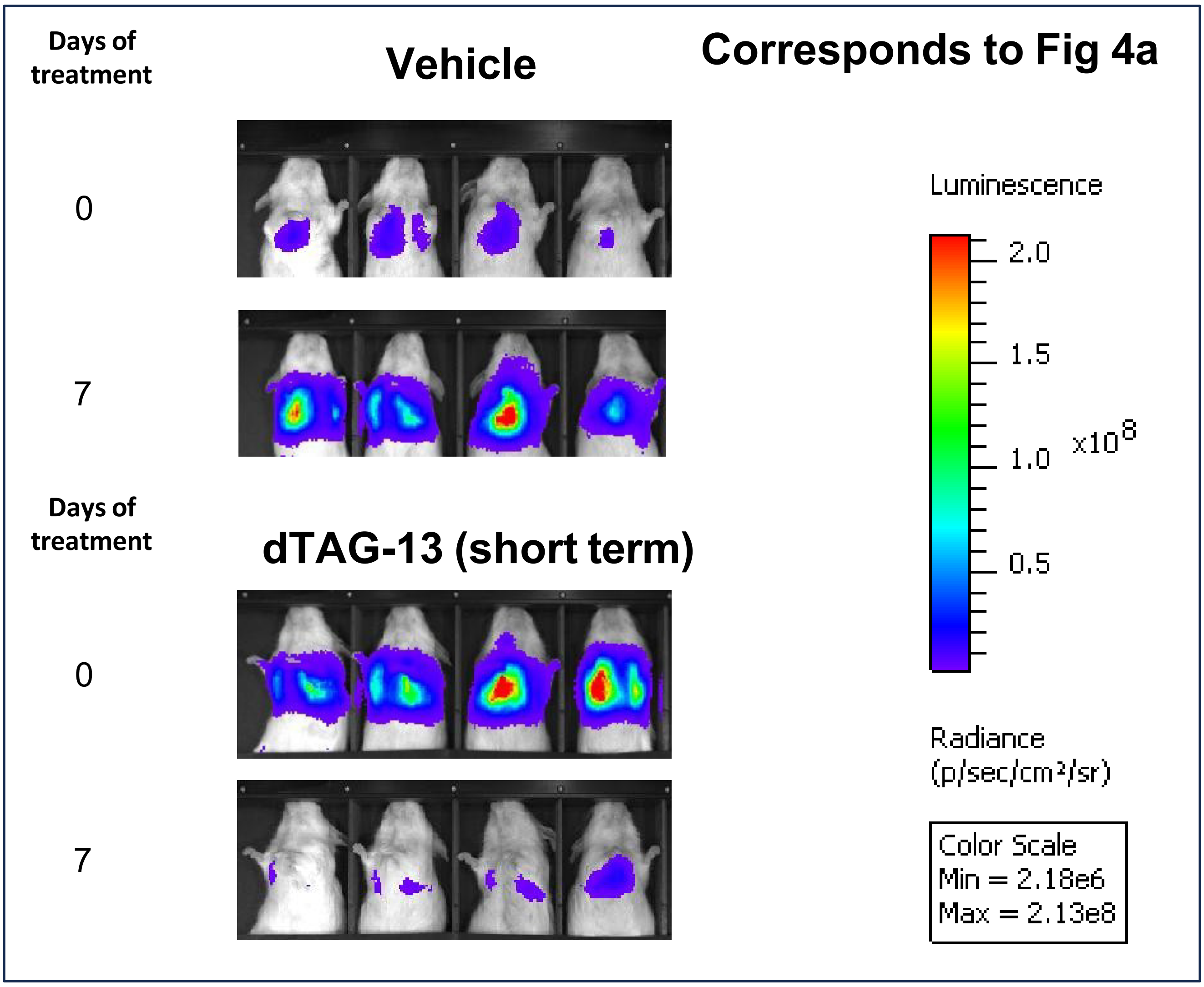

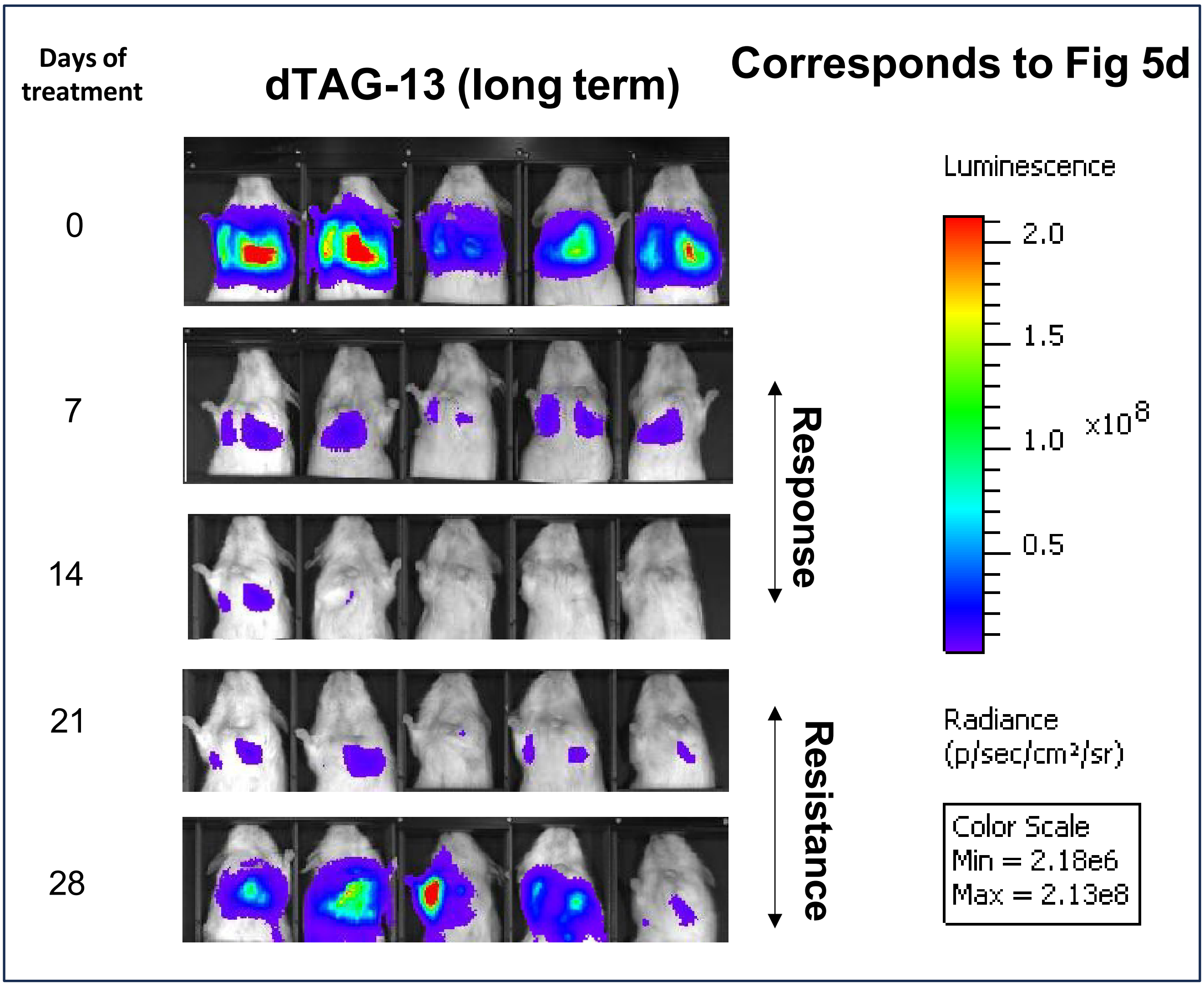

IVIS images showing tumor regression upon induction of KRAS^{G12V} degradation in C57BL/6J mice

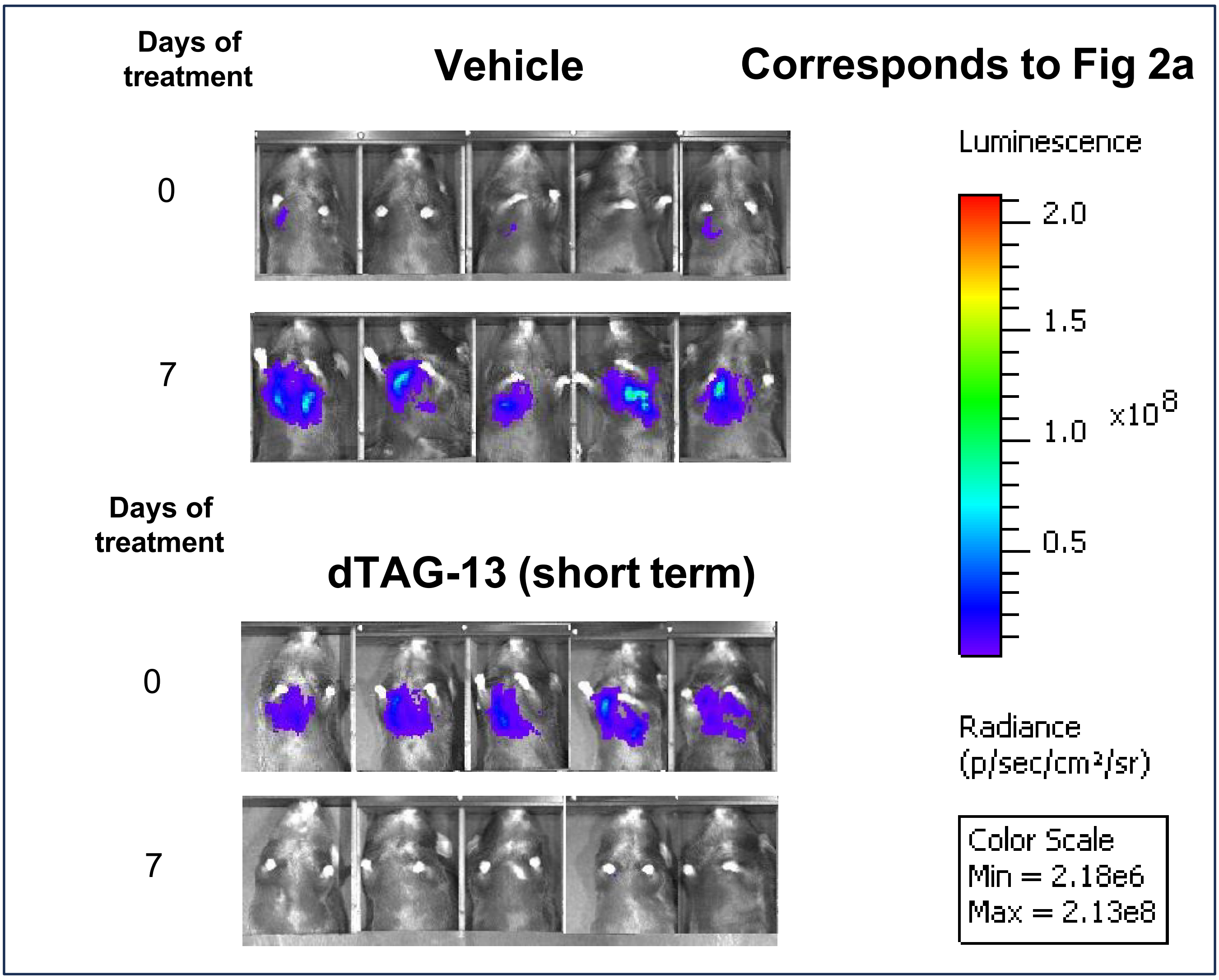

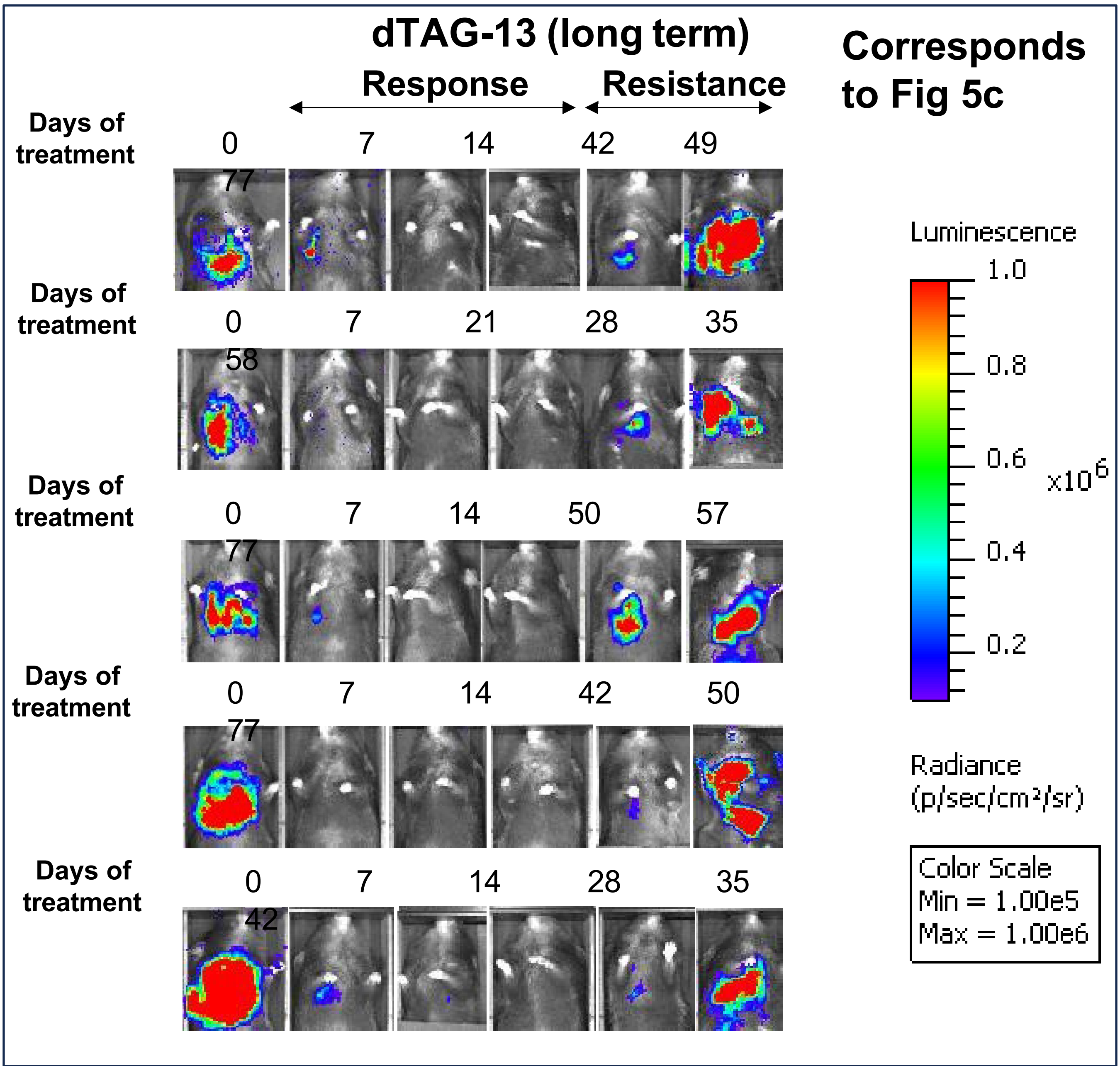

IVIS images showing limited short-term impact of CD8 immunodepletion on tumor regression in response to KRAS^{G12V} degradation

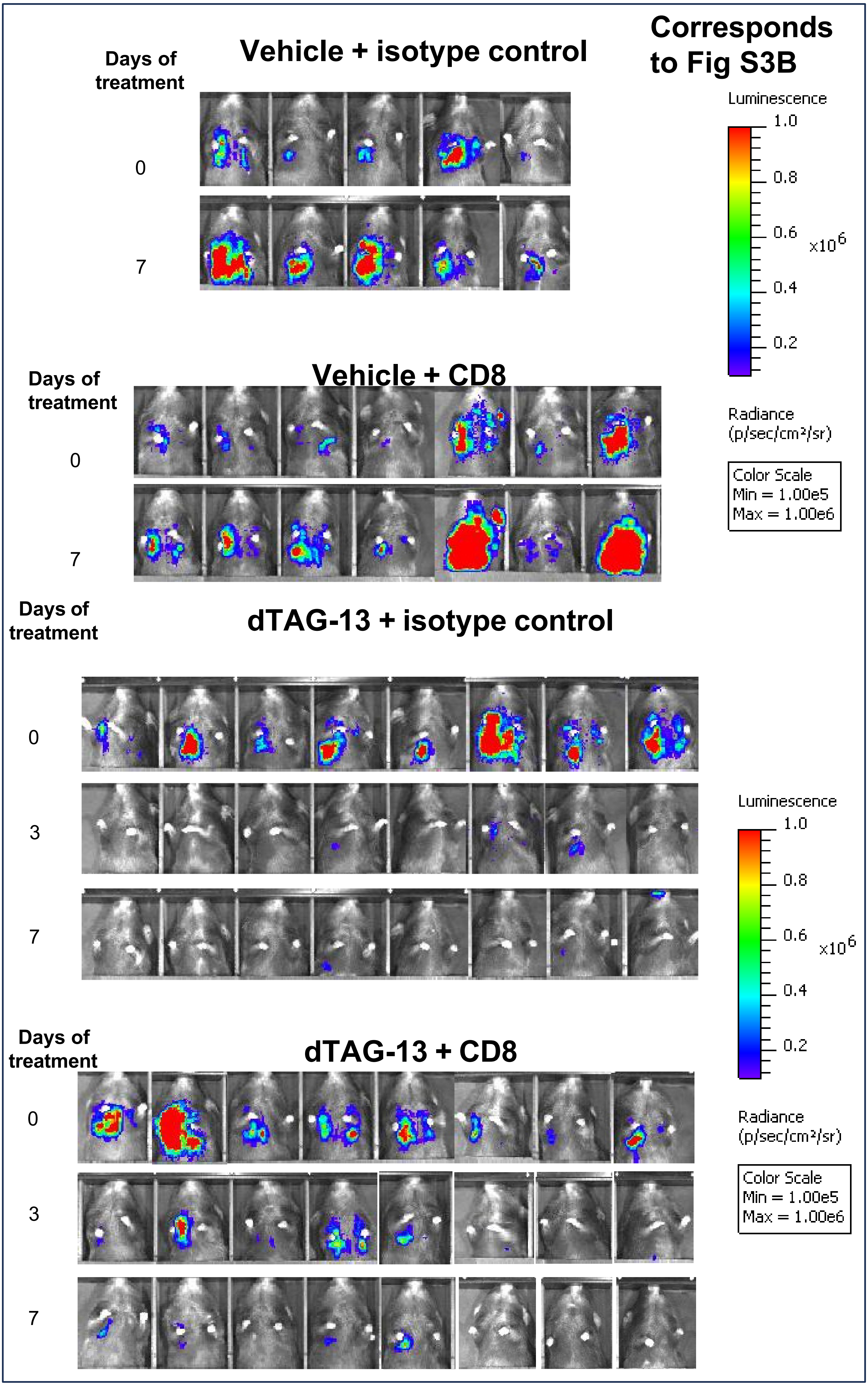

IVIS images showing the absence of long-term impact upon CD8 immunodepletion on tumor regression in response to KRAS^{G12V} degradation

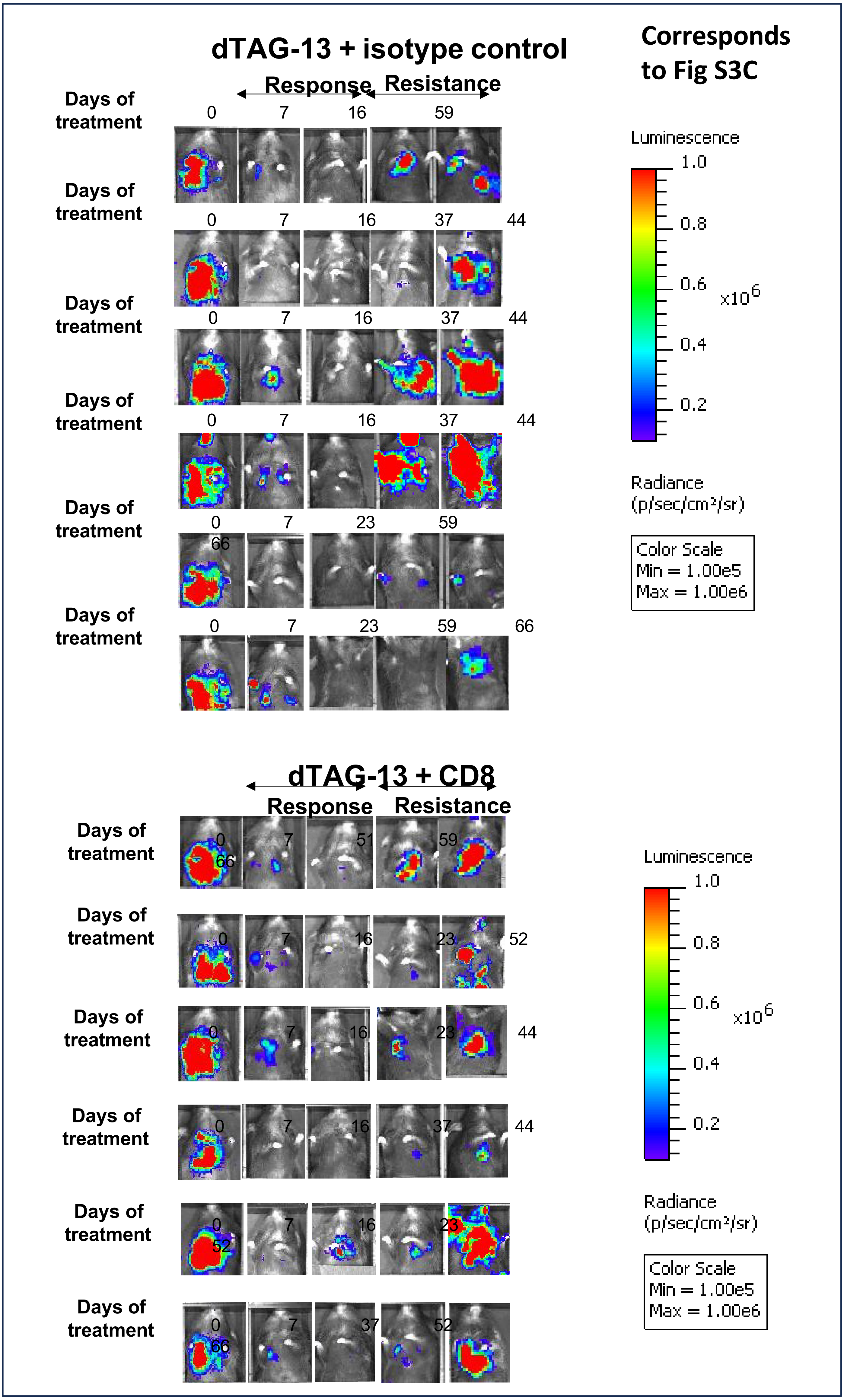

IVIS images depicting comparative antitumor response between KRAS^{G12V} inhibition and KRAS^{G12V} degradation (A) as well as the lack of effect of macrophage depletion on tumor regression upon degrader treatment (B)

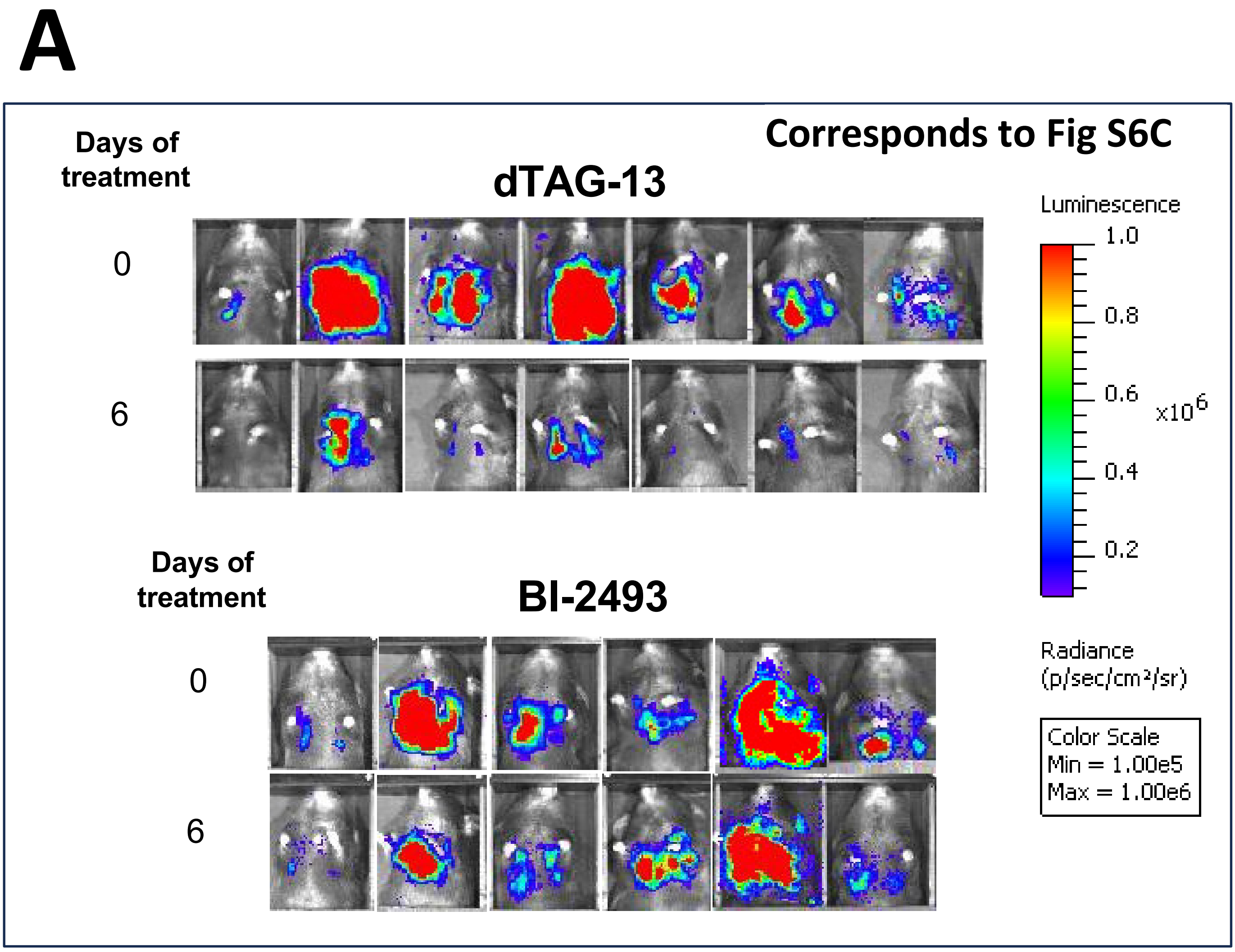

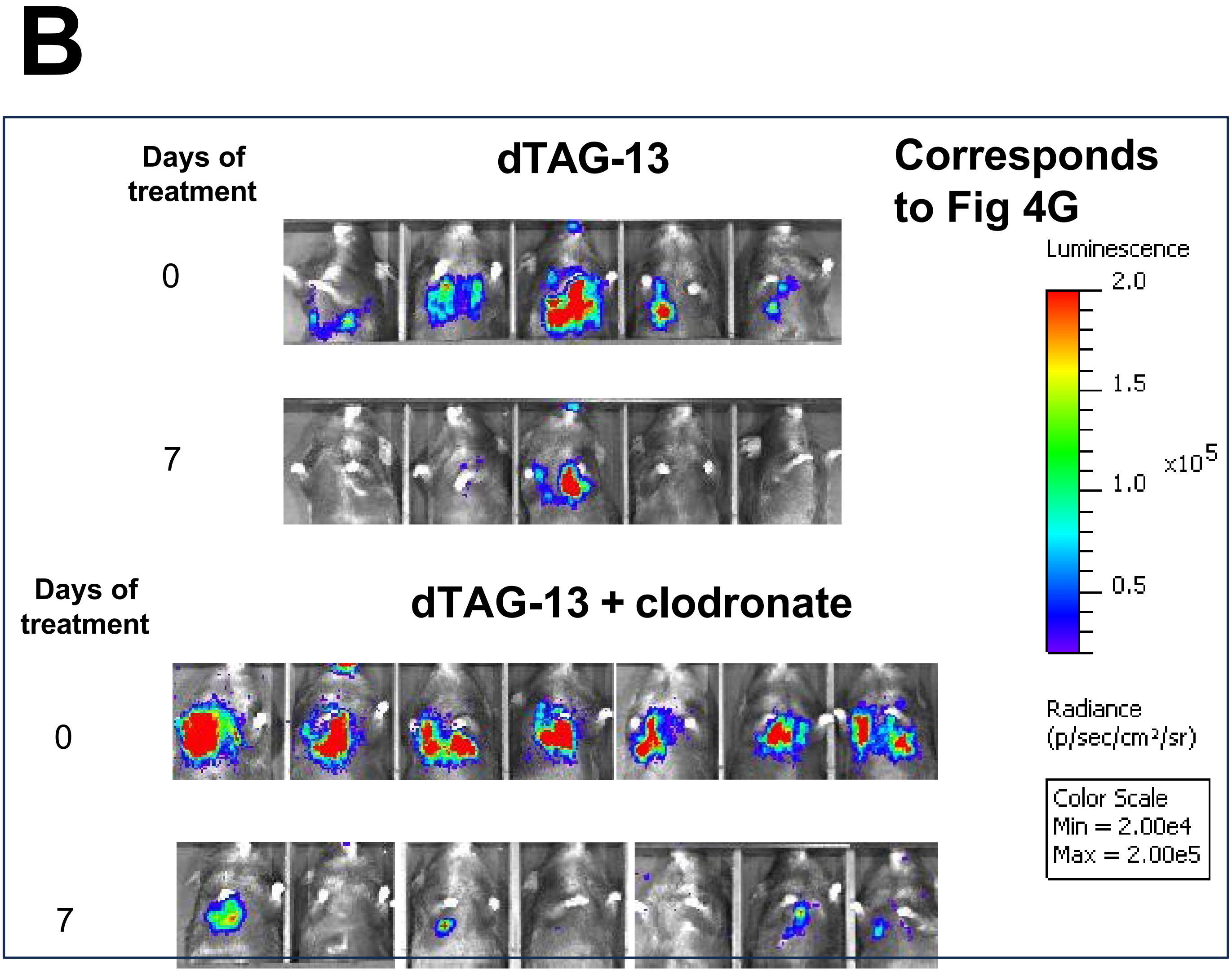

Supplement: 13 [file EMS214174-supplement-13.pdf]
